# Plant-Derived Flavonoids Ameliorate Diabetic Nephropathy: Molecular Mechanisms and Therapeutic Potential

**DOI:** 10.1016/j.advnut.2026.100741

**Published:** 2026-09-30

**Authors:** Enhui Tang, Shuyang Huang, Huangmin Zhou, Wenzhen Liao, Zheqing Zhang

**Affiliations:** Department of Nutrition and Food Hygiene, Guangdong Provincial Key Laboratory of Tropical Disease Research, School of Public Health, Southern Medical University, Guangzhou, China

**Keywords:** diabetic kidney disease, flavonoid metabolism, inflammation, oxidative stress, fibrosis, autophagy, ferroptosis

## Abstract

Diabetic nephropathy (DN) is a major microvascular complication of diabetes mellitus and a leading cause of chronic kidney disease and end-stage renal disease. Although current therapeutic strategies can delay disease progression, they remain insufficient to reverse established renal injury. Plant-derived flavonoids, a class of natural polyphenolic compounds, have attracted increasing attention because of their diverse pharmacologic properties. This structured narrative review summarizes the major molecular mechanisms underlying the protective effects of plant-derived flavonoids against DN. A comprehensive literature search was conducted in PubMed, Web of Science, and MEDLINE from database inception through 25 December 2025. A total of 251 relevant studies were included (54 in vitro only, 107 in vivo only, and 90 involving both). The evidence indicates that plant-derived flavonoids may exert renoprotective effects through diverse mechanisms, including the modulation of inflammation, oxidative stress, fibrosis, autophagy, apoptosis, ferroptosis, and microRNA-mediated regulatory pathways. Collectively, these flavonoids, such as kaempferol, quercetin, puerarin, and (-)-epigallocatechin-3-gallate, demonstrate multitarget therapeutic potential against DN and may serve as promising adjunctive agents for disease management. However, the current evidence is predominantly derived from preclinical studies, and well-designed randomized controlled trials are required to further validate their efficacy and safety and to facilitate clinical translation.

## Graphical abstract

**Figure:**
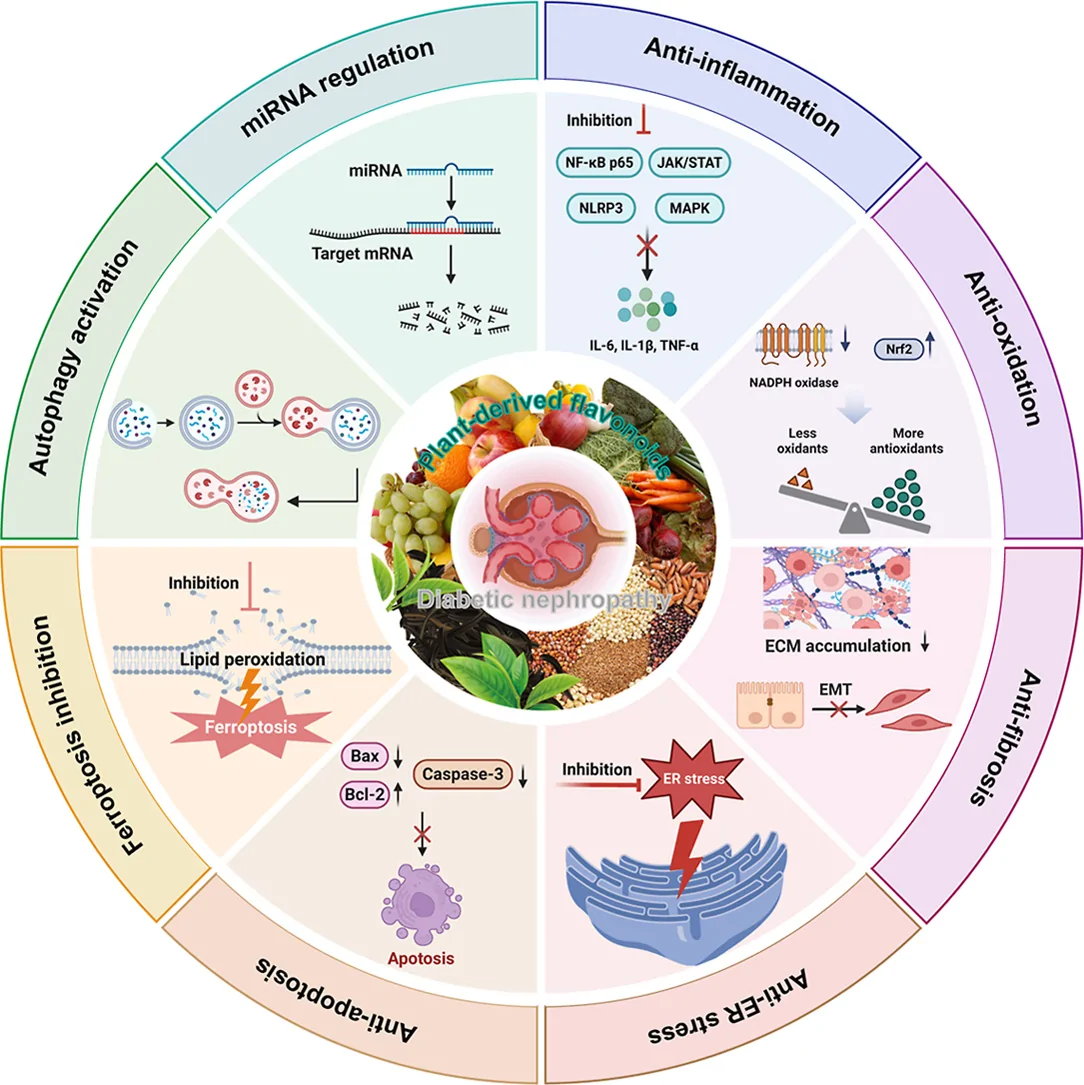

## Introduction

**Statement of Significance**

This structured narrative review, informed by a comprehensive literature search, summarizes preclinical evidence on plant-derived flavonoids in diabetic nephropathy, highlighting their diverse protective mechanisms, including regulation of inflammation, oxidative stress, fibrosis, autophagy, apoptosis, ferroptosis, and microRNA-mediated pathways.

Diabetic nephropathy (DN) is one of the most common microvascular complications of diabetes and a leading cause of chronic kidney disease and end-stage renal disease (ESRD) [1]. Epidemiological data indicate that ∼40% of patients with diabetes eventually develop DN [2,3]. With the continuous rise in the global prevalence of diabetes, the number of affected individuals is projected to reach 643 million and 783 million by 2030 and 2045, respectively, which will further exacerbate the burden of DN [4,5]. Therefore, DN remains a major global public health challenge [6]. The pathogenesis of DN is complex, and its characteristic renal structural changes include glomerular basement membrane thickening, mesangial matrix expansion, podocyte loss, glomerular hypertrophy, and tubulointerstitial fibrosis [[7], [8], [9]]. DN is primarily manifested as persistent increases in urinary albumin excretion and/or a decline in glomerular filtration rate [10]. The development and progression of DN are closely associated with multiple risk factors, including hyperglycemia, hypertension, dyslipidemia, genetic susceptibility, and lifestyle factors such as smoking and alcohol consumption [11]. Clinically, the management of DN is primarily based on glycemic control and blood pressure control, together with lifestyle interventions [12]. In addition, current kidney-protective pharmacotherapy mainly includes 4 major drug classes: renin-angiotensin-aldosterone system (RAAS) inhibitors, sodium-glucose cotransporter 2 (SGLT2) inhibitors, nonsteroidal mineralocorticoid receptor antagonists, and glucagon-like peptide-1 receptor agonists [13,14]. In patients with ESRD, renal replacement therapy, including dialysis and kidney transplantation, is required. Despite substantial advances in the treatment of DN, residual risk of kidney disease progression remains, highlighting the need for additional therapeutic strategies [15]. In recent years, accumulating evidence has demonstrated that various natural compounds possess potential value in the prevention and treatment of DN [[16], [17], [18], [19]]. Among them, plant-derived flavonoids have attracted considerable attention for their antioxidant, anti-inflammatory, and multitarget regulatory properties, showing promising therapeutic potential in the management of DN [20]. Several plant-derived flavonoids, such as kaempferol, quercetin, wogonin, (-)-epigallocatechin-3-gallate (EGCG), and puerarin, have shown renoprotective effects in experimental models of DN through diverse mechanisms, including anti-inflammatory, antioxidant, antifibrotic, and anti-apoptotic effects [[21], [22], [23], [24], [25]]. Previous reviews have summarized the therapeutic effects and certain molecular mechanisms of natural compounds (including flavonoids) in DN [[26], [27], [28]]; however, most of these reviews take a broad view across various components (e.g., polysaccharides, alkaloids) rather than focusing specifically on flavonoids. Therefore, a focused synthesis specifically addressing plant-derived flavonoids in DN remains limited. Accordingly, this review provides a comprehensive overview of the main molecular mechanisms by which flavonoids ameliorate DN, aiming to inform future research and clinical translation. Notably, in this review, “flavonoids” refers specifically to plant-derived flavonoids.

## Literature Search and Study Selection

This study was designed as a structured narrative review based on a comprehensive literature search to synthesize the major mechanistic evidence regarding flavonoids in DN. A systematic search of PubMed, Web of Science, and Medline was conducted from database inception through 25 December 2025 using MeSH terms and keywords related to “flavonoids” and (“diabetic nephropathy” or “diabetic kidney disease”). Only original experimental studies investigating the mechanisms by which pure flavonoid compounds ameliorate DN were included. Two reviewers independently screened titles and abstracts and assessed full-text articles, with disagreements resolved through discussion or consultation with a third reviewer. The overall literature search and study-selection process is summarized in Figure 1.

**FIGURE 1:**
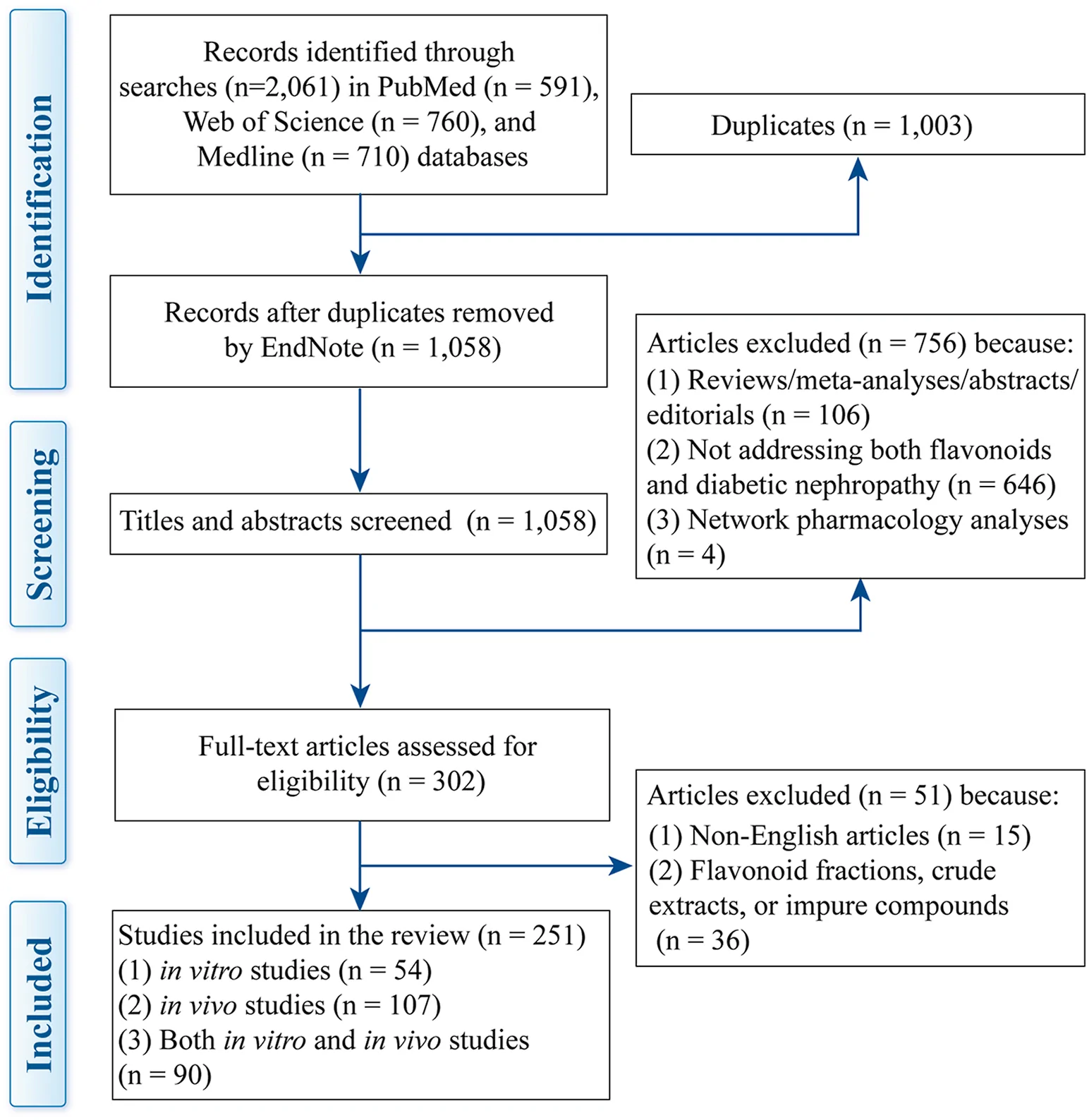
Flow diagram of the literature search and study selection process.

Database searches yielded 2061 records. After duplicates were removed using EndNote, 1058 articles remained. After screening of titles and abstracts, 756 records were excluded, including reviews, meta-analyses, editorials, abstracts, network pharmacology studies, and studies that did not address both flavonoids and DN. Of the 302 full-text articles assessed for eligibility, 51 were excluded because they were non-English or involved flavonoid fractions, crude extracts, or impure compounds. Ultimately, 251 original experimental studies met the eligibility criteria and were included in this review. Among these studies, 54 involved only in vitro experiments, 107 involved only in vivo experiments, and 90 included both in vitro and in vivo experiments. Each eligible publication was counted as one study and assigned to one mutually exclusive category; studies reporting both in vitro and in vivo data were classified as combined and counted only once. Because the primary aim of this review was to provide a structured synthesis of major mechanistic evidence rather than quantitatively estimate intervention effects or formally assess therapeutic efficacy, no formal study-level risk-of-bias assessment or certainty-of-evidence grading was performed. Therefore, the findings should be interpreted as a structured synthesis of the available mechanistic evidence. Detailed study-level information for all 251 eligible studies is provided in Supplemental Table 1.

## Flavonoids and Diabetic Nephropathy

### Pathophysiology of diabetic nephropathy

DN is a severe complication of diabetes mellitus, and its pathogenesis involves complex interactions among multiple mechanisms, with hyperglycemia serving as a central driving factor. Persistent hyperglycemia promotes the generation of reactive oxygen species (ROS) and advanced glycation end products and activates protein kinase C. These factors trigger downstream signaling pathways that mediate inflammation, fibrosis, and other pathogenic processes, ultimately leading to renal injury and accelerating the progression of DN [29]. Early pathophysiological features of DN include glomerular hyperfiltration, which is closely associated with elevated glomerular capillary pressure and may lead to the subsequent development of albuminuria and renal injury [30,31]. Hyperglycemia not only contributes to dilation of the renal afferent arteriole but also activates the RAAS, promoting local angiotensin II (Ang II) production in the kidney [[32], [33], [34]]. Increased Ang II preferentially constricts the efferent arteriole, thereby increasing intraglomerular pressure and further contributing to glomerular hyperfiltration [35,36]. In addition, dysregulated lipid metabolism and insulin resistance [37], epigenetic modifications [38], endoplasmic reticulum (ER) stress [39], and mitochondrial dysfunction and mitophagy [40] also play important roles in the progression of DN.

### Structures of flavonoids

Flavonoids, a prominent class of plant polyphenolic secondary metabolites, are widely distributed in a variety of plant-derived foods, including fruits, vegetables, cereals, and tea [41]. They possess a typical C6–C3–C6 skeleton, comprising 15 carbon atoms, including 2 aromatic rings (A and B rings) and an oxygen-containing heterocyclic ring (C ring) formed by a three-carbon bridge. On the basis of the degree of oxidation of the C ring and the linkage pattern between the B and C rings, flavonoids can be classified into subgroups, including flavones, flavanones, flavanols, flavonols, isoflavones, and anthocyanidins [42]. The hydroxyl groups on the A and B rings provide the basis for a variety of chemical modifications, including glycosylation, acylation, and methylation. Chalcones, as important precursors in flavonoid biosynthesis, are also frequently involved in studies of the broader flavonoid family [43]. In nature, most flavonoids occur as glycosides, whereas some exist in the aglycone form. As widely distributed natural products, flavonoids generally exhibit relatively low toxicity and good biocompatibility; they also possess diverse biologic activities and potential therapeutic value [44]. Numerous studies have shown that plant-derived flavonoids play an important role in the prevention and treatment of DN (Supplemental Table 1). This review thus summarizes relevant studies, with a particular focus on the major molecular mechanisms by which flavonoids ameliorate DN, aiming to provide a reference for the development of flavonoids as potential therapeutic agents and for their clinical translation. Representative experimental studies supporting the major molecular mechanisms discussed here are summarized in Table 1. The structures of the flavonoids discussed in this review are shown in Figure 2. It should be noted that the current evidence supporting the renoprotective effects and molecular mechanisms of flavonoids in DN is mainly derived from in vitro and animal studies, whereas clinical evidence remains limited.

**TABLE 1:** Flavonoids and diabetic nephropathy

Subclass	Compounds	Model	Treatment methods	Molecular mechanisms	Reference
Flavonol	Kaempferol	STZ-induced diabetic C57BL/6J mice	10 mg/kg/d (i.g.) for 14 wk	Downregulated TFR6 expression and inhibited the NF-κB pathway	[45]
		High glucose-induced NRK-52E cells	2.5 μM for 1 h		
		*db/db* mice	50, 100 mg/kg/d (i.g.) for 12 wk	Inhibited apoptosis and promoted autophagy through the AMPK/mTOR pathway	[46]
		Unilateral nephrectomy + STZ-induced DN in SD rats	50, 100, 200 mg/kg/d (i.g.) for 12 wk	Activated PINK1/Parkin-mediated mitophagy	[47]
	Quercetin	*db/db* mice	25, 100 mg/kg/d, (i.g.) for 12 wk	Activated the Nrf2/HO-1 pathway to inhibit ferroptosis	[48]
		High glucose-induced HK-2 cells	25 μM for 48 h		
		STZ-induced diabetic SD rats	100 mg/kg/d (i.g.) for 12 wk	Activated the Nrf2 pathway to inhibit ferroptosis	[49]
		High glucose-induced HK-2 cells	25 μM for 48 h		
		High glucose-induced NRK-52E cells	10, 20, 40 μM for 24 h	Inhibited the USP22–Snail1 signaling axis to block EMT	[50]
		HFD+STZ-induced diabetic C57BL/6J mice	150 mg/kg/d (i.g.) for 4 wk	Inhibited renal tubular epithelial cell apoptosis via the PI3K/AKT pathway	[51]
		High glucose-induced HK-2 cells	25 μM for 48 h		
		*db/db* mice	50, 100, 150 mg/kg/d (i.g.) for 8 wk	Attenuates podocyte apoptosis through targeting the EGFR pathway	[52]
		High glucose-induced conditionally immortalized mouse podocytes	10, 20, 40 μM for 24 h		
		High glucose-induced HMCs	10, 20, 40 μM for 48 h	Attenuated proliferation, inflammation, and oxidative stress by regulating the miR-485-5p/YAP1 pathway	[53]
	Formononetin	*db/db* mice	30, 60 mg/kg/d (i.g.) for 12 wk	Activated autophagy by inhibiting the PI3K/AKT/mTOR pathway	[54]
Flavonol	Fisetin	STZ-induced eNOS^-/-^ C57BL/6J mice	5, 10, 20 mg/kg/d (i.g.) for 8 wk	Activated the Nrf2/HO-1 pathway to inhibit ferroptosis	[55]
Flavonol glycoside	Hyperoside	HFD+STZ-induced diabetic C57BL/6J mice	30 mg/kg/d (i.g.) for 4 wk	Targeted the miR-499-5p/APC axis	[56]
	Icariin	High glucose-induced conditionally immortalized mouse podocytes	10 μM for 48 h	Activated GPER to inhibit apoptosis	[57]
		HFHSD+STZ-induced diabetic SD rats	20, 40, 80 mg/kg/d (i.g.) for 12 wk	Restored autophagy via regulation of the miR-192-5p/GLP-1R pathway	[58]
		High glucose-induced HK-2 and NRK-49F cells	2, 10, 50 μM for 48 h		
	Isoquercitrin	db/db mice	40, 80, 120 mg/kg/d (i.g.) for 12 wk	Inhibited STAT3 phosphorylation and dimerization	[59]
		High glucose- or IL-6-induced HUVECs and IL-6-induced HK-2 cells	5, 10, 20 μM		
	Rutin	*db/db* mice	100, 200 mg/kg/d (i.g.) for 8 wk	Activated autophagy by inhibiting the PI3K/AKT/mTOR pathway	[60]
Flavanol	(+)-Catechin	HFD+STZ-induced diabetic C57BL/6J mice	50, 100 mg/kg/d (i.g.) for 8 wk	Inhibited ER stress-related NLRP3 inflammasome activation	[61]
		High glucose-induced HK-2 cells	5, 10, 20 μM for 48 h	Inhibited EMT	[62]
	EGCG	HFD+STZ-induced DN in Wistar rats	100 mg/kg/d (i.g.) for 30 d	Activated the Keap-1/Nrf2/ARE pathway, inhibited NOX2 activity, and thereby alleviated oxidative stress	[63]
		PA-induced HK-2 cells	50, 75, 100 μM for 24 h		
		HFD+STZ-induced DN in Wistar rats	100 mg/kg/d (i.g.) for 4 wk	Inhibited apoptosis	[64]
	(-)-Epicatechin	High glucose + LPS-induced NRK-52E cells	1, 5, 10 μM for 24 h	Inhibited NOX4/MAPK-mediated inflammation	[65]
Flavone	Apigenin	STZ-induced diabetic Wistar rats	5, 10, 20 mg/kg/d (i.g.) for 8 months	Reduced oxidative stress, alleviated renal fibrosis, and inhibited MAPK-mediated inflammation	[66]
	Chrysin	*db/db* mice	10 mg/kg/d (i.g.) for 10 wk	Inhibited the PERK-eIF2α-ATF-CHOP pathway	[67]
	Luteolin	STZ-induced DN in Nrf2+/+ and Nrf2−/− C57BL/6 mice	25, 50 mg/kg/d (i.g.) for 10 wk	Activated Nrf2 signaling to reduce oxidative stress, inflammation, and TGF-β1-mediated ECM deposition	[68]
		High glucose-induced SV40-MES13 mouse mesangial cells	62.5, 125, 250 nM for 24 h		
	Tangeretin	*db*/*db* mice	10 mg/kg/d (i.g.) for 10 wk	Inhibited EMT	[69]
		High glucose-induced conditionally immortalized mouse podocytes	1, 10, 20 μM for 4 d		
	Wogonin	*db*/*db* mice	40 mg/kg/every other day (i.g.) for 12 wk	Upregulated SOCS3 to alleviate renal injury by inhibiting the TLR4-mediated JAK/STAT/AIM2 pathway	[70]
		STZ-induced diabetic C57BL/6J mice	10, 20, 40 mg/kg/every other day (i.g.) for 16 wk	Activated autophagy by inhibiting the PI3K/AKT/mTOR pathway	[71]
		STZ-induced diabetic C57BL/6J mice	10, 20, 40 mg/kg/every other day (i.g.) for 12 wk	Inhibited NF-κB and TGF-β/Smad signaling pathways to ameliorate renal inflammation and fibrosis.	[72]
		High glucose-induced SV-40 cells	1.5825, 3.125, 6.25 μg/mL for 24 h		
		STZ-induced diabetic C57BL/6J mice	10, 20, 40 mg/kg/every other day (i.g.) for 12 wk	Targeted Bcl-2-mediated autophagy and apoptosis	[73]
Flavone glycoside	Baicalin	High glucose + TNF-α + AGEs-induced HK-2 cells	5, 10 μM for 72 h or 96 h	Inhibited the NF-κB and JAK/STAT pathways	[74]
		*db/db* mice	400 mg/kg/d (i.g.) for 8 wk	Activated the Nrf2 pathway to reduce oxidative stress and inhibited the MAPK-mediated inflammation	[75]
Flavone glycoside	Diosmin	High glucose-induced HK-2 cells	10 μg/mL for 48 h	Regulated the PTEN PI3K/AKT axis to attenuate ER stress	[76]
	Scutellarin	STZ-induced diabetic C57BL/6J mice	10, 40 mg/kg/d (i.g.) for 4 wk	Blocked the TGF-β/Smad pathway	[77]
	Vitexin	HFD/STZ-induced DN in SD rats	10, 20 mg/kg/d (i.p.) for 4 wk	Upregulated GPX4-mediated antioxidant defense	[78]
		High glucose-induced HK-2 cells	10, 20 μM for 24 h		
	Wogonoside	HFD+STZ-induced diabetic C57BL/6J mice	12.5, 25, 50 mg/kg/d (i.g.) for 10 wk	modulated the HNF4A-NRF2 axis to alleviate oxidative stress	[79]
Flavanone	Eriodictyol	High glucose-induced HMCs	12.5, 25 μM for 24 h	Attenuate oxidative stress, inflammation, and extracellular matrix accumulation	[80]
	Naringenin	High glucose-induced HK-2 cells	50, 100, 200 μM for 48 h	Activated the SIRT1/FOXO3a axis and Nrf2 pathway to inhibit ferroptosis	[81]
Isoflavone	Calycosin	db/db mice	10, 20 mg/kg/d (i.p.) for 4 wk	Restored the GSH–GPX4 antioxidant defense, downregulated NCOA4 expression, and attenuated lipid peroxidation	[82]
		High glucose-induced HK-2 cells	10, 20, 40 μM for 24 h		
	Formononetin	db/db mice	25, 50 mg/kg/d, (i.g.) for 8 wk	Activated the Nrf2/ARE pathway via Sirt1	[83]
Isoflavone glycoside	Puerarin	High glucose-induced HBZY1 cells	1, 10 μM for 24 h	Inhibited ferroptosis	[84]
Isoflavone glycoside	Puerarin	STZ-induced DN in C57BL/6J mice	10, 20, 40 mg/kg/d, (i.g.) for 12 wk	Modulated the SIRT1/NLRP3/caspase-1 pathway	[85]
		High glucose-induced MPC-5 cells	0.8 mM for 24 h		
Chalcone	Isoliquiritigenin	HFD+STZ-induced diabetic C57BL/6J mice	10, 30 mg/kg/d (i.g.) for 10 wk	Reduced oxidative stress and inhibited the TLR4/NF-κB/NLRP3 pathway	[86]
		High glucose-induced NRK-52E cells	20 μM for 24 h		
		STZ-induced diabetic C57BL/6J mice	10, 20 mg/kg/every 2 days (i.g.) for 12 wk	Reduced oxidative stress and inhibited MAPK-mediated inflammation	[87]
		High glucose-induced NRK-52E cells	10, 20 μM for 1 h		
		High glucose-induced HMCs	1, 10, 20 μM for 72 h	Blocked the TGF-β/Smad pathway to reduce mesangial matrix accumulation	[88]
Flavanonol	Astilbin	High glucose-induced HBZY-1 cells	10, 20 μg/mL for 24 h	Suppressed the TLR4/MyD88/NF-κB pathway	[89]
	Dihydromyricetin	High glucose-induced HMCs	10,20 μM for 24 h	Activated the Nrf2/HO-1 pathway, inhibited NOX2/NOX4 activity, and thereby alleviated oxidative stress	[90]
		Uninephrectomy + STZ-induced DN in SD rats	100 mg/kg/d (s.c.) for 10 wk	Promoted autophagy and attenuated renal interstitial fibrosis by regulating miR-155-5p/PTEN pathway	[91]
		High glucose-induced NRK-52E cells	1 μM for 24 or 48 h		
	Taxifolin	HFD/STZ-induced DN in SD rats	25, 50, 100 mg/kg/d for 12 wk	Suppressed ROS generation and NLRP3 inflammasome activation	[16]
		High glucose-induced HBZY-1 cells and HK-2 cells	5, 10, 20 μM for 72 h		
Anthocyanin	Cyanidin-3-O-glucoside	STZ-induced DN in SD rats	10, 20 mg/kg/d (i.g.) for 8 wk	Attenuated oxidative stress and inflammation and downregulated TGF-β1/Smad2/3 signaling	[92]

Table 1 presents representative experimental studies that directly illustrate the major molecular mechanisms discussed in the main text and does not constitute the complete list of eligible studies. Detailed study-level information for all 251 eligible experimental studies is provided in Supplemental Table 1.

**FIGURE 2:**
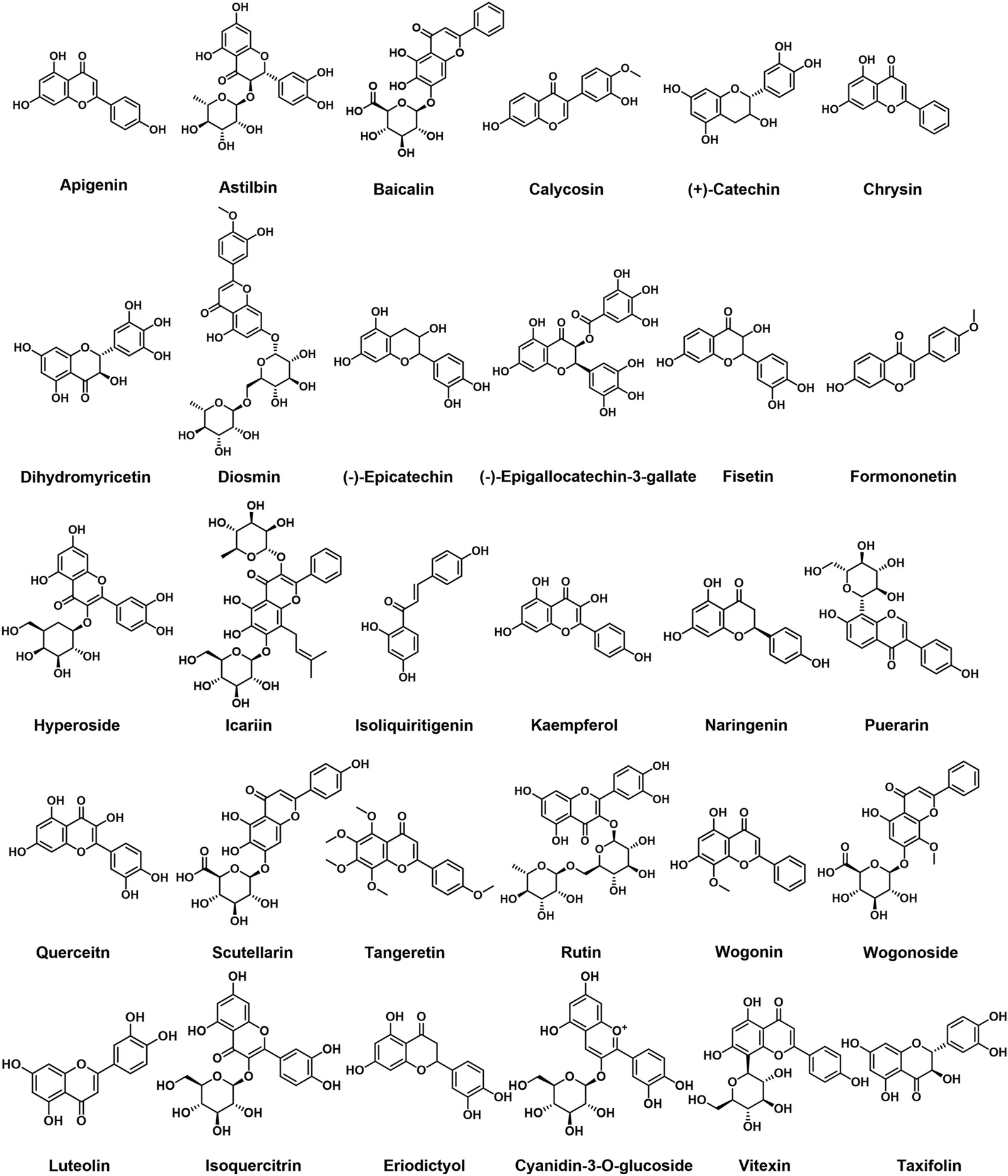
The chemical structures of flavonoids.

### Molecular mechanisms by which flavonoids ameliorate diabetic nephropathy

#### Effects of flavonoids on inflammatory processes in diabetic nephropathy

The inflammatory response is recognized as an important driver of the onset and progression of DN [93]. Accumulating evidence indicates that various flavonoids modulate key inflammatory signaling pathways, leading to the suppression of downstream proinflammatory cytokines (such as IL-6, TNF-α, IL-1β, and MCP-1), thereby exerting anti-inflammatory and protective effects on the kidney (Figure 3) [16,45,59,61,65,66,70,74,75,[85], [86], [87],^89^]. Astilbin and kaempferol individually inhibit the assembly of the toll-like receptor 4 (TLR4)/myeloid differentiation primary response 88 signaling complex and the activation of tumor necrosis factor receptor-associated factor 6, thereby blocking nuclear translocation of nuclear factor-κB (NF-κB) p65 and reducing the production of proinflammatory cytokines IL-6 and TNF-α [45,89]. (+)-Catechin, puerarin, and taxifolin suppress NOD-like receptor family pyrin domain containing 3 (NLRP3) inflammasome activation, reducing the maturation and release of IL-1β and IL-18, and thus alleviating inflammatory responses [16,61,85]. Notably, puerarin suppresses NLRP3 inflammasome activation and downstream caspase-1/gasdermin D–mediated pyroptosis by upregulating sirtuin 1 (SIRT1). The involvement of SIRT1 was further supported by pharmacologic inhibition with EX527 in streptozotocin (STZ)-induced DN mice and by SIRT1 silencing and overexpression in high glucose-induced MPC-5 podocytes. Moreover, Isoquercitrin directly binds to the SH2 domain of signal transducer and activator of transcription 3 (STAT3), inhibiting its phosphorylation and dimerization and reducing IL-6, IL-1β, TNF-α, and MCP-1 expression [59]. A subset of flavonoids can simultaneously regulate multiple signaling pathways, exerting convergent inhibition on inflammatory responses. For example, isoliquiritigenin suppresses TLR4/NF-κB signaling and reduces ROS accumulation, thereby attenuating the priming and activation of the NLRP3 inflammasome [86]. Wogonin upregulates suppressor of cytokine signaling 3 (SOCS3) to suppress TLR4 expression, thereby inhibiting the Janus kinase 2 (JAK2)/STAT3 signaling pathway and absent in melanoma 2 inflammasome activation. This SOCS3/TLR4-dependent mechanism was further supported by complementary overexpression and knockdown experiments in high glucose-treated HK-2 cells and by coknockdown rescue in *db/db* mice [70]. Baicalin inhibits JAK2 phosphorylation and STAT3 activation, while also suppressing the NF-κB signaling pathway through the inhibition of inhibitor of nuclear factor-κB alpha degradation and NF-κB nuclear translocation, leading to the downregulation of proinflammatory cytokines, including IL-1β, IL-6, and MCP-1 [74]. In addition, broad inhibition of mitogen-activated protein kinase (MAPK) phosphorylation (extracellular signal-regulated kinase 1/2 [ERK1/2], c-Jun N-terminal kinase, and p38) represents a common anti-inflammatory mechanism shared by several flavonoids, including isoliquiritigenin, baicalin, apigenin, and (-)-epicatechin [65,66,75,87]. Collectively, these studies demonstrate that certain flavonoids exert anti-inflammatory effects in DN by inhibiting several key inflammatory signaling pathways, including NF-κB, the NLRP3 inflammasome, JAK2/STAT3, and MAPK.

**FIGURE 3:**
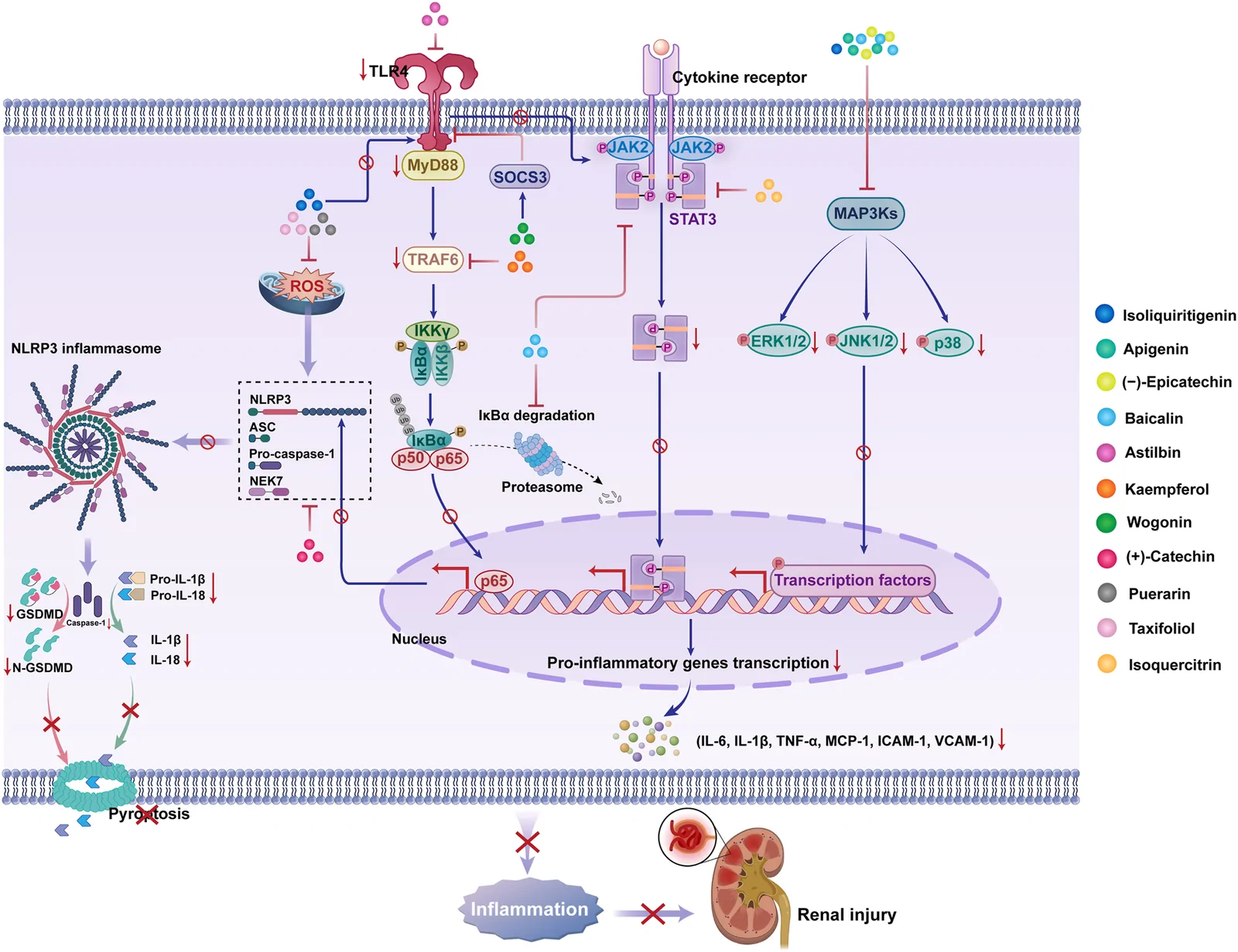
Anti-inflammatory mechanisms of flavonoids in DN. Flavonoids attenuate renal inflammation in DN by regulating NF-κB, NLRP3 inflammasome, JAK2/STAT3, and MAPK signaling pathways. ASC, apoptosis-associated speck-like protein containing a CARD; ERK1/2, extracellular signal-regulated kinase 1/2; GSDMD, gasdermin D; IKKβ, inhibitor of NF-κB kinase subunit β; IKKγ (NEMO), inhibitor of NF-κB kinase subunit γ; IκBα, inhibitor of NF-κB alpha; JAK2, Janus kinase 2; JNK1/2, c-Jun N-terminal kinase 1/2; MAP3Ks, mitogen-activated protein kinase kinase kinases; MyD88, myeloid differentiation primary response 88; N-GSDMD, N-terminal fragment of gasdermin D; NLRP3, NOD-like receptor family pyrin domain containing 3; ROS, reactive oxygen species; SIRT1, sirtuin 1; SOCS3, suppressor of cytokine signaling 3; STAT3, signal transducer and activator of transcription 3; TLR4, Toll-like receptor 4; TRAF6, tumor necrosis factor receptor-associated factor 6.

#### Effects of flavonoids on oxidative stress in diabetic nephropathy

Oxidative stress is widely considered a central mechanism mediating hyperglycemia-induced diabetic microvascular complications, particularly DN [94]. Flavonoids have been widely reported to exert renoprotective effects in cellular and animal models of DN via multitarget modulation of redox homeostasis. Their mechanisms of action are not limited to activating downstream antioxidant defense systems; certain compounds can also directly interfere with the sources of ROS generation, reflecting coordinated regulation of oxidative stress at both upstream and downstream levels (Figure 4) [63,68,79,80,83,90]. For example, EGCG exerts coordinated protective effects against oxidative stress in DN [63]. On the one hand, EGCG suppresses the expression of NADPH oxidase isoform 2 (NOX2), thereby reducing the production of superoxide anion (O_2_·^-^), the primary ROS generated by NOX2. On the other hand, EGCG downregulates Kelch-like ECH-associated protein 1 expression, promotes the nuclear translocation of nuclear factor erythroid 2-related factor 2 (Nrf2), and enhances its binding activity to antioxidant response elements. These effects subsequently lead to the upregulation of downstream antioxidant proteins, including heme oxygenase-1 (HO-1), glutamate-cysteine ligase catalytic subunit, and NAD(P)H quinone dehydrogenase 1. Consequently, EGCG increases the activities of superoxide dismutase, catalase, and glutathione peroxidase (GSH-Px), as well as the levels of reduced glutathione (GSH), collectively alleviating oxidative stress-mediated renal injury. Similarly, both dihydromyricetin and eriodictyol inhibit NOX2 and NADPH oxidase isoform 4 expression, thereby reducing ROS accumulation and malondialdehyde (MDA) levels in high glucose-stimulated mesangial cells, whereas dihydromyricetin additionally activates Nrf2/HO-1 signaling [80,90]. Notably, wogonoside and formononetin have been shown to regulate upstream modulators of Nrf2 [83,84]. Wogonoside upregulates hepatocyte nuclear factor 4 alpha, whereas formononetin activates SIRT1, thereby enhancing Nrf2 signaling and upregulating its downstream antioxidant targets, ultimately alleviating renal injury in DN. Further supporting the central role of Nrf2-dependent antioxidant defense, luteolin increased Nrf2 and its downstream antioxidant proteins and reduced renal oxidative damage in STZ-induced diabetic mice, whereas these effects were largely absent in Nrf2^-^/^-^ mice, supporting an Nrf2-dependent mechanism [68]. Collectively, current evidence indicates that reducing excessive ROS production and restoring redox homeostasis in DN represents an important mechanism through which flavonoids exert renoprotective effects. Among them, representative compounds such as EGCG and dihydromyricetin achieve a coordinated modulation of oxidative stress by simultaneously inhibiting NADPH oxidase (NOX)-derived ROS generation and activating Nrf2-associated antioxidant responses. These findings provide important mechanistic insights into the multitarget properties of flavonoid-based natural products and support their potential as therapeutic candidates for DN.

**FIGURE 4:**
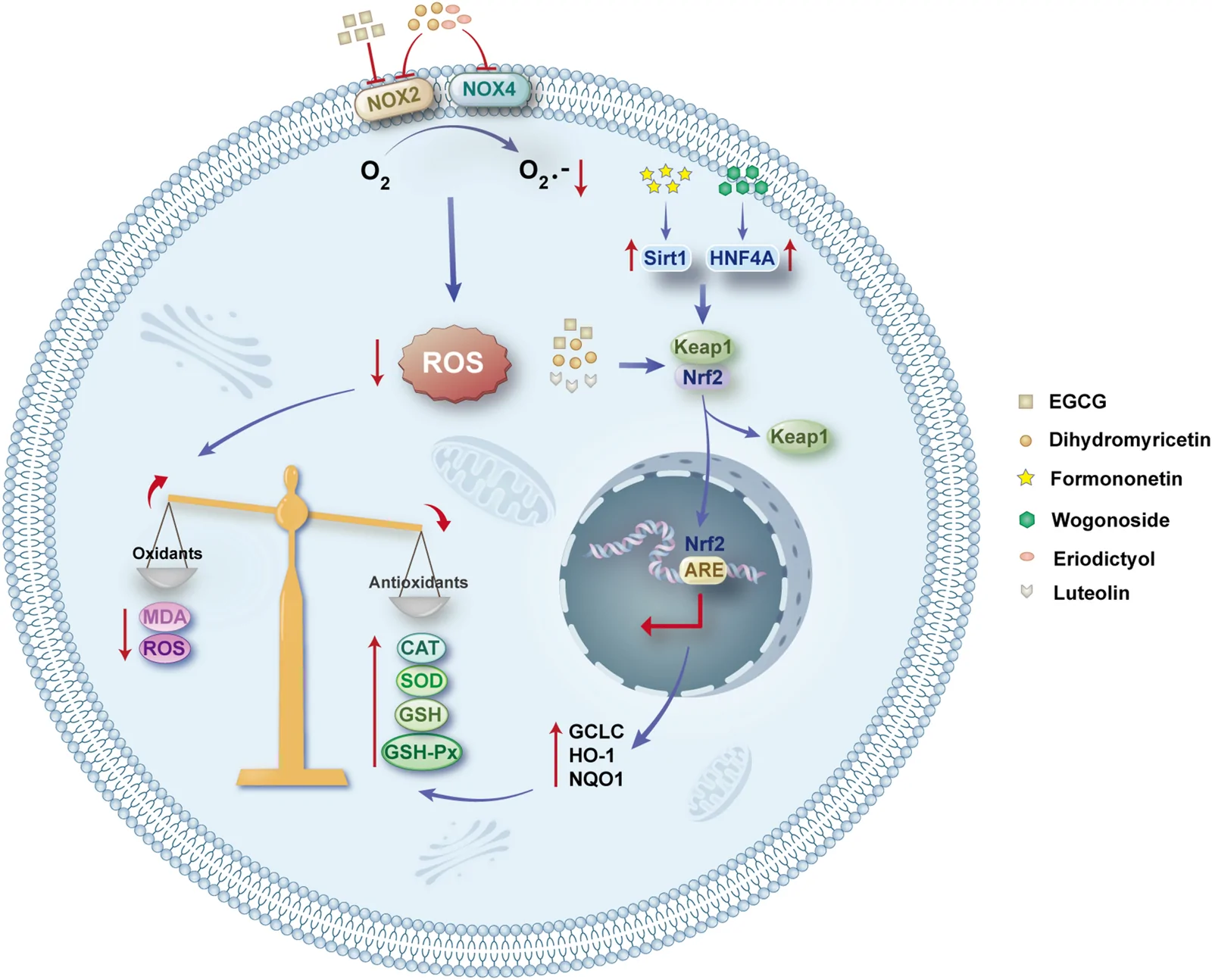
Antioxidant mechanisms of flavonoids in DN. Flavonoids exert renoprotective effects in DN by inhibiting NOX-derived ROS production and activating the Nrf2 signaling pathway, thereby enhancing antioxidant defenses, suppressing oxidative stress, and attenuating renal injury. ARE, antioxidant response element; CAT, catalase; EGCG, (-)-epigallocatechin-3-gallate; GCLC, glutamate-cysteine ligase catalytic subunit; GSH, glutathione; GSH-Px, glutathione peroxidase; hepatocyte nuclear factor 4 alpha; HO-1, heme oxygenase-1; Keap1, Kelch-like ECH-associated protein 1; MDA, malondialdehyde; NOX2, NADPH oxidase isoform 2; NOX4, NADPH oxidase isoform 4; NQO1, NAD(P)H quinone dehydrogenase 1; Nrf2, nuclear factor erythroid 2–related factor 2; ROS, reactive oxygen species; SIRT1, sirtuin 1; SOD, superoxide dismutase.

#### Effects of flavonoids on renal fibrosis in diabetic nephropathy

The hallmark structural lesion of DN is renal fibrosis, characterized primarily by excessive deposition of extracellular matrix (ECM) [95]. Several studies have demonstrated that multiple flavonoids exert antifibrotic effects in DN by inhibiting the transforming growth factor-β (TGF-β)/Smad signaling pathway (Figure 5) [72,77,88,92]. Overactivation of this pathway serves as a central driver of renal fibrosis by promoting ECM accumulation and myofibroblast activation [96]. In high glucose-stimulated human renal mesangial cells, isoliquiritigenin attenuates TGF-β1/TGF-β receptor I/II (TGF-βRI/II)-mediated signaling, as evidenced by reduced Smad2 phosphorylation, downregulation of Smad4, and upregulation of inhibitory Smad7 [88]. In parallel, a shift toward reduced ECM accumulation and enhanced ECM turnover was observed, as reflected by decreased expression of ECM-related molecules, including connective tissue growth factor and collagen type IV, alongside increased membrane type-1 matrix metalloproteinase and decreased tissue inhibitor of metalloproteinase-2. These findings suggest that isoliquiritigenin may exert renoprotective effects by modulating the TGF-β/Smad signaling pathway to regulate ECM homeostasis. In STZ-induced DN mice, scutellarin attenuates renal fibrosis, and related pathological alterations, as evidenced by decreased expression of collagen type III alpha 1 and α-smooth muscle actin (α-SMA) [77]. These effects are associated with decreased TGF-β1 expression, reduced Smad2/3 phosphorylation, and downregulation of Smad4. Similarly, cyanidin-3-O-glucoside downregulated TGF-β1 expression and inhibited Smad2/3 phosphorylation while restoring Smad7 expression in STZ-induced DN rats, accompanied by attenuation of glomerular sclerosis and interstitial fibrosis [92]. In addition, in STZ-induced DN mice, wogonin suppresses TGF-β1/Smad3 signaling and downregulates ECM-related proteins and myofibroblast-associated markers [72]. Similar effects are observed in high glucose-stimulated mesangial cells. Collectively, current evidence indicates that flavonoids exert antifibrotic effects in DN by suppressing the TGF-β/Smad signaling axis, thereby regulating ECM synthesis and degradation. However, compound-specific differences in molecular targets (eg, TGF-β1, its receptors, and distinct Smad isoforms) and experimental systems (cellular compared with animal models) require further investigation to strengthen the robustness and generalizability of these mechanistic insights.

**FIGURE 5:**
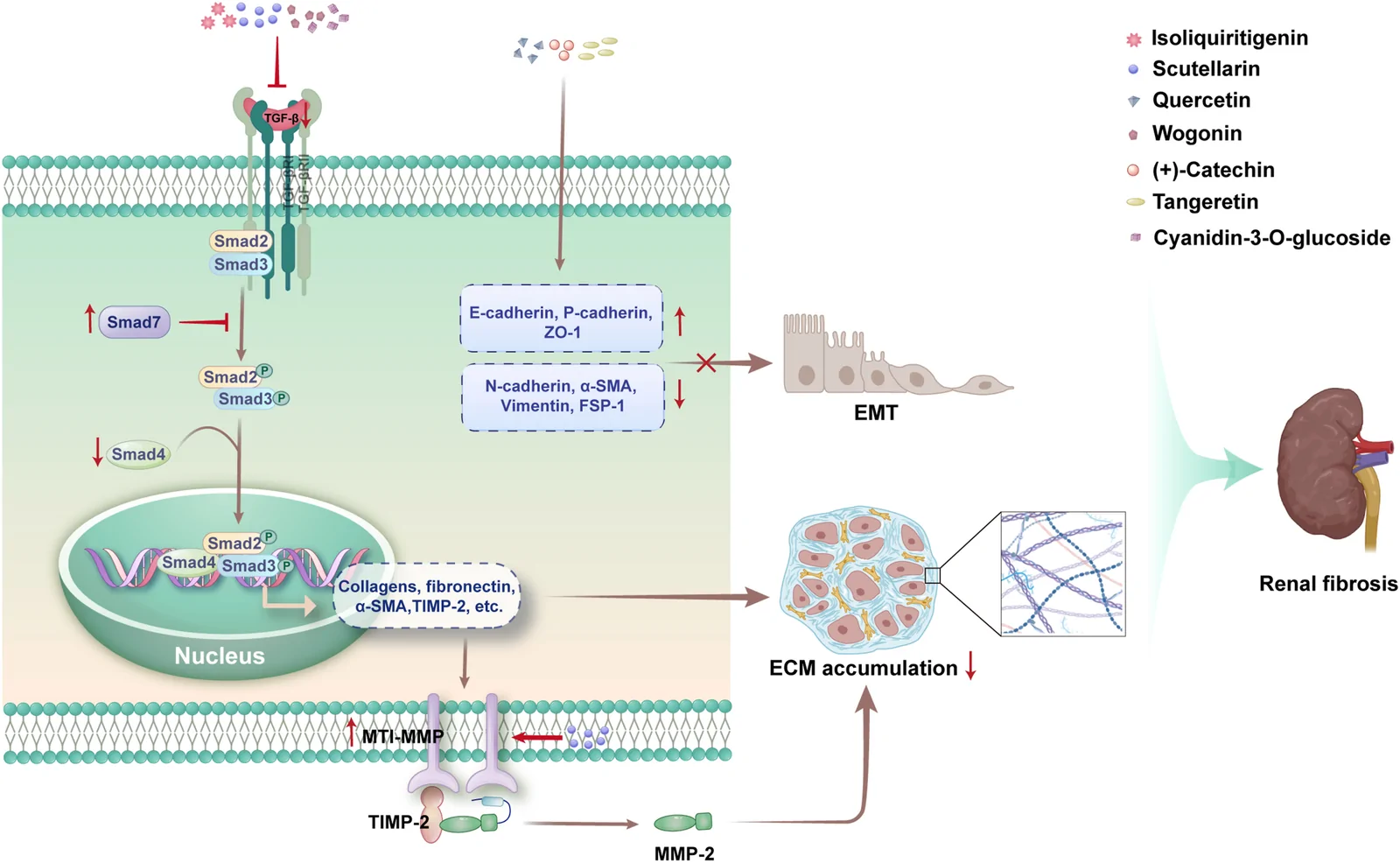
Antifibrotic mechanisms of flavonoids in DN. Flavonoids attenuate renal fibrosis in DN by suppressing TGF-β/Smad-mediated ECM accumulation, inhibiting EMT in renal tubular epithelial cells, and mitigating EMT-like phenotypic changes in podocytes. ECM, extracellular matrix; EMT, epithelial-mesenchymal transition; FSP-1, fibroblast-specific protein 1; MMP2, matrix metalloproteinase 2; MT1-MMP, membrane type 1 matrix metalloproteinase; TGF-β, transforming growth factor-β; TGF-βRI/II, transforming growth factor-β receptor I/II; TIMP-2, tissue inhibitor of metalloproteinase-2; α-SMA, α-smooth muscle actin.

Epithelial-mesenchymal transition (EMT) is a process by which epithelial cells progressively acquire mesenchymal phenotypic characteristics and is recognized as an important contributor to fibrosis development [97,98]. In DN, both partial EMT in renal tubular epithelial cells and podocyte dedifferentiation or EMT-like phenotypic transition have been implicated in the progression of tubulointerstitial fibrosis and glomerulosclerosis. Emerging evidence suggests that multiple flavonoids exert renoprotective effects by suppressing EMT-associated processes (Figure 5) [50,62,69]. In high glucose-induced HK-2 cells, (+)-catechin attenuates EMT-associated phenotypic changes, as evidenced by increased E-cadherin expression and reduced α-SMA levels [72]. Similarly, in *db*/*db* mice and high glucose-stimulated conditionally immortalized mouse podocytes, tangeretin mitigates EMT-like phenotypic alterations, characterized by upregulation of E-cadherin and P-cadherin, downregulation of N-cadherin, and suppression of α-SMA and fibroblast-specific protein 1, thereby alleviating podocyte injury and fibrotic responses [69]. Additionally, in *db*/*db* mice and high glucose-stimulated renal tubular epithelial cells (NRK-52E), quercetin attenuates EMT-associated alterations and reduces ECM accumulation [50]. Mechanistically, these effects are attributed, at least in part, to suppression of the ubiquitin-specific protease 22-Snail1 axis, leading to enhanced ubiquitination and degradation of the transcription factor Snail1. Taken together, flavonoids attenuate EMT or EMT-like phenotypic changes in DN-related cellular and animal models, thereby alleviating podocyte and tubular injury and suppressing fibrotic progression.

#### Effects of flavonoids on apoptosis in diabetic nephropathy

During the progression of DN, apoptosis induced by hyperglycemia and metabolic disturbances, particularly via the mitochondrial pathway, is considered a key pathologic mechanism underlying irreversible podocyte loss and renal dysfunction. Recent studies have indicated that various flavonoids can alleviate renal injury via regulation of this apoptotic pathway (Figure 6) [46,51,52,57,64,73]. Specifically, although flavonoids such as EGCG, quercetin, wogonin, and kaempferol differ in chemical structure, they consistently upregulate the antiapoptotic protein B-cell lymphoma 2 (Bcl-2), downregulate the pro-apoptotic protein Bcl-2-associated X protein, and inhibit the activation of caspase-3, thereby exerting anti-apoptotic effects [46,51,52,57,64]. For instance, quercetin acts by suppressing the epidermal growth factor receptor-ERK1/2 signaling axis [52] and inhibiting aberrant activation of phosphoinositide 3-kinase (PI3K)/protein kinase B (AKT) signaling [51], whereas wogonin has been reported to directly bind to and stabilize Bcl-2 [73]. In addition, icariin activates G protein–coupled estrogen receptor (GPER), promotes mitochondrial localization of Bcl-2, maintains mitochondrial membrane potential, and reduces the release of cytochrome c and apoptosis-inducing factor, thereby inhibiting podocyte apoptosis [57]. Overall, these findings suggest that flavonoids modulate multiple upstream targets and ultimately converge on the canonical mitochondrial apoptotic pathway, thereby alleviating diabetic renal injury.

**FIGURE 6:**
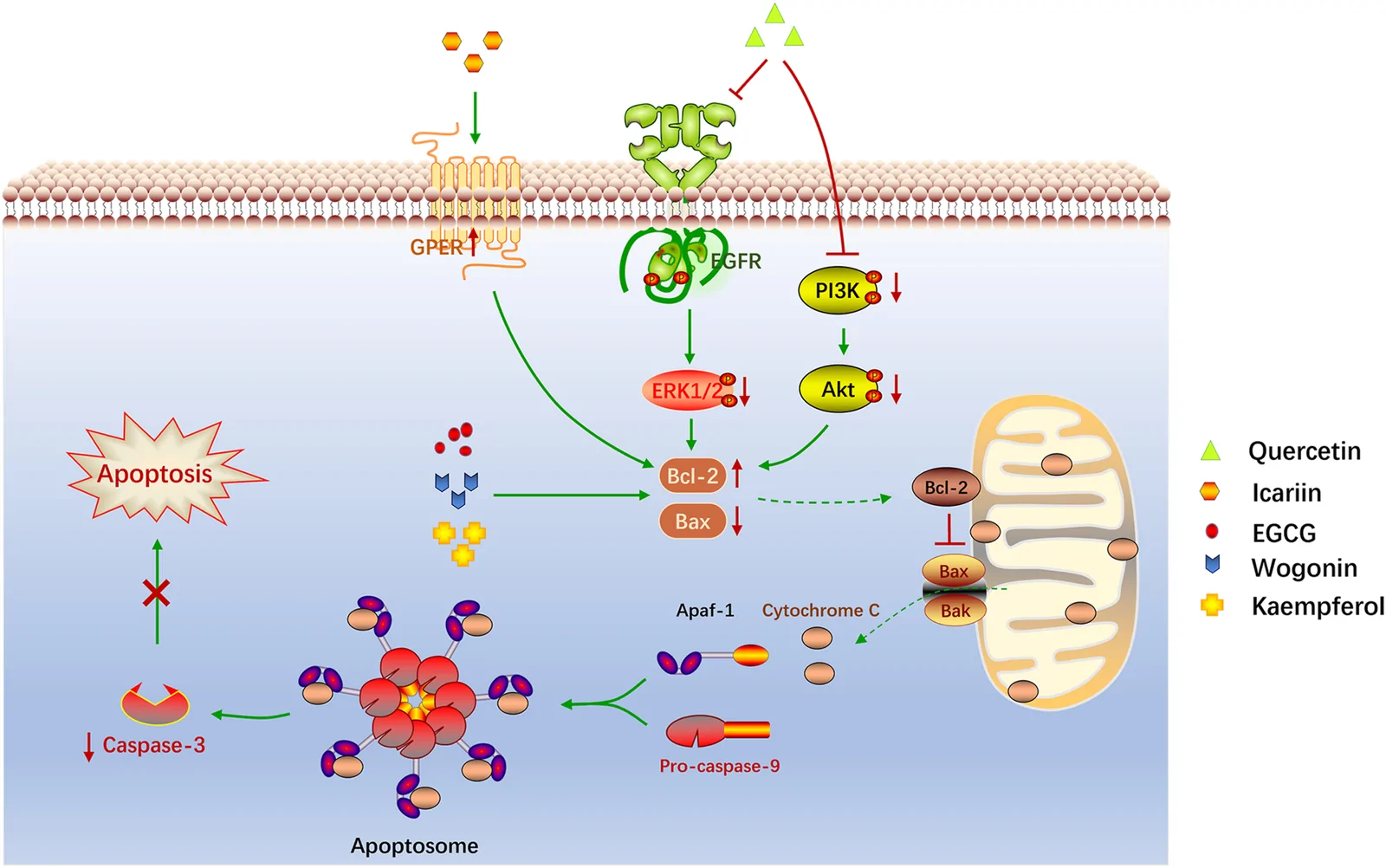
Antiapoptotic mechanisms of flavonoids in DN. Flavonoids alleviate DN-associated renal injury by inhibiting apoptotic processes, primarily through the mitochondrial apoptotic pathway. Despite structural diversity, they act on multiple upstream targets and ultimately converge on Bcl-2/Bax-mediated regulation of mitochondrial membrane permeability, thereby suppressing caspase-3 activation and exerting anti-apoptotic effects. AKT, protein kinase B; Bax, Bcl-2-associated X protein; Bcl-2, B-cell lymphoma 2; EGFR, epidermal growth factor receptor; GPER, G protein-coupled estrogen receptor; PI3K, phosphoinositide 3-kinase.

#### Effects of flavonoids on endoplasmic reticulum stress in diabetic nephropathy

The ER is a highly dynamic organelle that plays essential roles in various fundamental cellular processes, including protein folding and modification, protein quality control, and lipid synthesis [99]. However, the accumulation of misfolded or unfolded proteins within the ER lumen under diverse pathologic conditions triggers a specific cellular stress response, referred to as ER stress [100]. In DN, ER stress and its downstream unfolded protein response (UPR), which consists of 3 major signaling branches, exhibit dual roles: the early adaptive UPR confers cytoprotective effects, whereas sustained ER stress triggers proapoptotic UPR signaling that drives the progression of renal injury [101]. Studies have shown that the protective effects of various flavonoids against diabetic renal injury are associated with the inhibition of ER stress (Figure 7) [61,67,76,102]. Kang et al. [67] demonstrated that chrysin exerts renoprotective effects in *db*/*db* mice and high glucose-stimulated podocytes by suppressing the protein kinase RNA-like endoplasmic reticulum kinase (PERK)-eukaryotic initiation factor 2α (eIF2α)-activating transcription factor 4 (ATF4)-C/EBP homologous protein (CHOP) signaling axis, a key branch of the ER stress-related UPR. Specifically, chrysin reduces the phosphorylation levels of PERK and eIF2α, downregulates ATF4 and CHOP expression, thereby alleviating ER stress-mediated podocyte apoptosis. Consequently, it restores the expression of slit diaphragm proteins nephrin and podocin, ameliorates podocyte foot process effacement, and ultimately reduces proteinuria while improving glomerular filtration barrier dysfunction. Zhang et al. [61] found that (+)-catechin downregulates glucose-regulated protein 78 (GRP78; also known as binding immunoglobulin protein) and inhibits ER stress-related signaling pathways, including the PERK-CHOP axis and the ATF6 pathway, thereby alleviating ER dilation and injury in renal tubular epithelial cells. This study further showed that suppression of ER stress attenuates NLRP3 inflammasome-mediated inflammatory responses, ultimately improving renal injury. Deng et al. [76] showed that diosmin reduces the expression of GRP78 and CHOP in high glucose-stimulated HK-2 cells, which in turn attenuates ER stress-associated apoptosis. The protective mechanism may involve modulation of the phosphatase and tensin homolog (PTEN)-PI3K/AKT signaling axis: diosmin restores PTEN levels and normalizes aberrant PI3K/AKT phosphorylation, thereby suppressing ER stress. Notably, icariin is reported to enhance GPER expression and reduce inositol-requiring enzyme 1 and spliced X-box binding protein 1, thereby attenuating ER stress in high glucose-stimulated rat renal tubular epithelial cells [102]. In summary, the ER stress/UPR signaling pathway represents an important mechanism by which flavonoids ameliorate diabetic renal injury.

**FIGURE 7:**
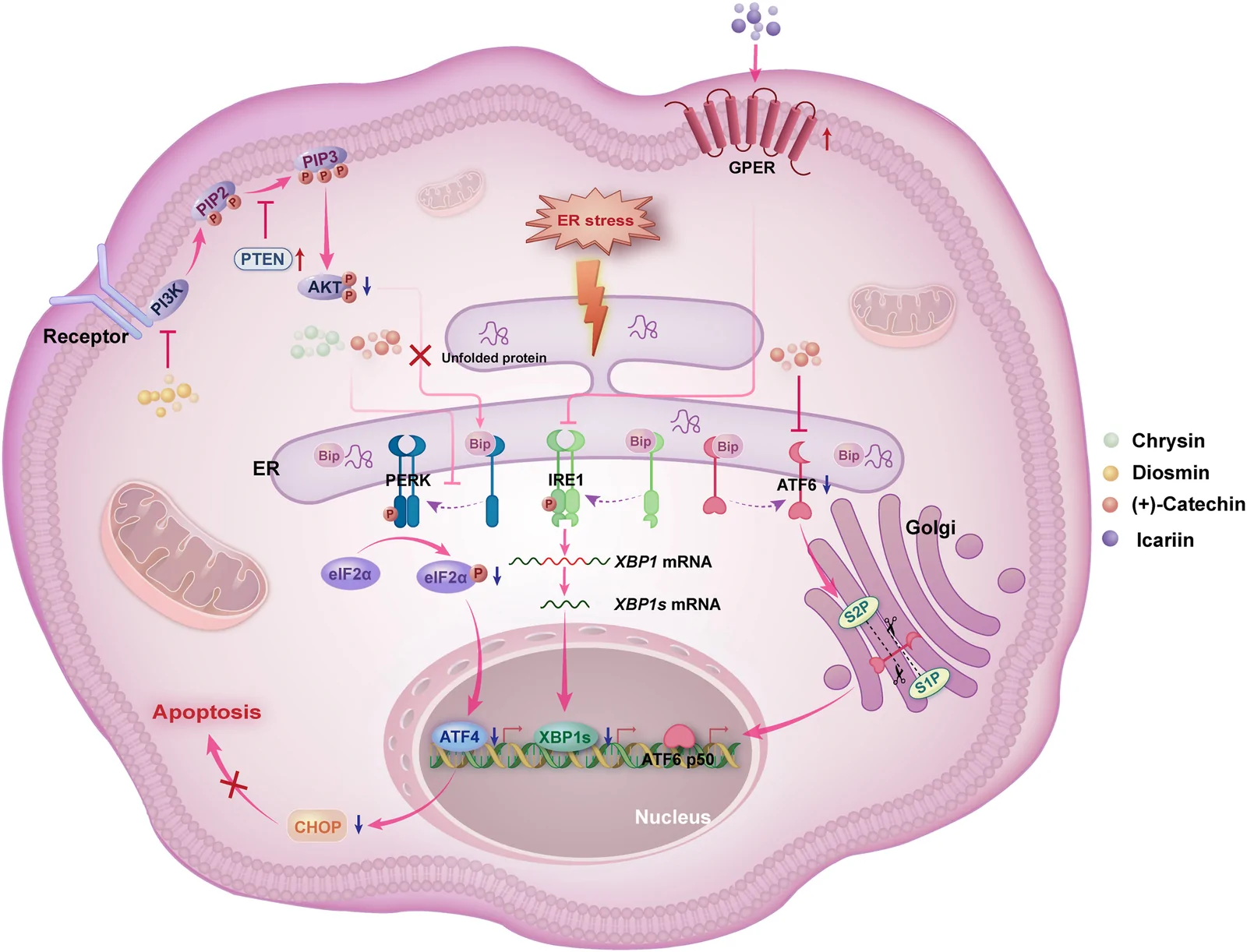
Anti-ER stress mechanisms of flavonoids in DN. Flavonoids attenuate ER stress and the UPR signaling pathways, including the PERK-eIF2α-ATF4-CHOP, ATF6, and IRE1-XBP1 branches, thereby reducing apoptosis and inflammatory responses in DN-related models and contributing to renoprotective effects. ATF4, activating transcription factor 4; ATF6, activating transcription factor 6; CHOP, C/EBP homologous protein; eIF2α, eukaryotic initiation factor 2α; ER, endoplasmic reticulum; GADD34, growth arrest and DNA damage-inducible protein 34; GPER, G protein-coupled estrogen receptor; GRP78/BiP, glucose-regulated protein 78 (binding immunoglobulin protein); IRE1, inositol-requiring enzyme 1; PERK, protein kinase RNA-like endoplasmic reticulum kinase; PIP2, phosphatidylinositol (4,5)-bisphosphate; PIP3, phosphatidylinositol (3,4,5)-trisphosphate; PTEN, phosphatase and tensin homolog; S1P, site-1 protease; S2P, site-2 protease; UPR, unfolded protein response; XBP1, X-box binding protein 1.

#### Effects of flavonoids on ferroptosis in diabetic nephropathy

Ferroptosis is a form of regulated cell death characterized by iron-dependent accumulation of lipid peroxides [103]. In recent years, ferroptosis has been increasingly implicated in the pathogenesis of DN [104,105]. Available evidence indicates that multiple flavonoids, including calycosin, puerarin, vitexin, naringenin, quercetin, and fisetin, may exert renoprotective effects by inhibiting ferroptosis in renal cells, particularly in renal tubular epithelial cells, podocytes, and mesangial cells [48,49,55,78,81,82,84]. Overall, these compounds exert their effects mainly by maintaining intracellular iron homeostasis, restoring the system Xc^-^/GSH/glutathione peroxidase 4 (GPX4) antioxidant defense system, or regulating Nrf2-related antioxidant signaling pathways, thereby suppressing ferroptosis (Figure 8). For example, calycosin has been reported to restore the GSH/GPX4 system, downregulate nuclear receptor coactivator 4 expression, and reduce lipid peroxidation levels (e.g., lipid ROS and MDA) in high glucose-stimulated HK-2 cells and *db/db* mice [82]. These protective effects can be partially reversed by erastin (a classic ferroptosis inducer), suggesting that inhibition of ferroptosis may be one of the key mechanisms underlying its renoprotective actions. Moreover, puerarin reduces Fe^2+^ accumulation, restores GSH levels, upregulates GPX4, and increases the expression of key subunits of the system Xc^-^ (cystine/glutamate antiporter), namely solute carrier family 7 member 11 (SLC7A11) and solute carrier family 3 member 2, while downregulating acyl-CoA synthetase long-chain family member 4 in high glucose-stimulated HBZY-1 cells, thereby maintaining iron homeostasis and suppressing ferroptosis [84]. Similarly, in high glucose-stimulated HK-2 cells and DN rats, vitexin suppresses ferroptosis by restoring GPX4 and SLC7A11 expression, reducing Fe^2+^ accumulation and lipid peroxidation, and increasing GSH levels [78]. Notably, GPX4 knockdown partially reverses these protective effects, supporting a GPX4-dependent anti-ferroptotic mechanism. Furthermore, naringenin, quercetin, and fisetin share a common mechanism involving the activation of the Nrf2-centered antioxidant signaling network, including the Nrf2/GPX4 axis, thereby restoring cellular redox homeostasis [48,49,55,81]. Zhou et al. [81] demonstrated that naringenin activates the SIRT1/forkhead box O3a signaling pathway and coordinates with Nrf2-mediated antioxidant responses, thus attenuating ferroptosis in high glucose-stimulated HK-2 cells. In *db*/*db* mice and high glucose-stimulated HK-2 cells, quercetin has been reported to inhibit ferroptosis in renal tubular epithelial cells via activation of the Nrf2/HO-1 signaling pathway [48]. This inhibitory effect is evidenced by reduced iron content and lipid peroxidation levels (including MDA and 4-hydroxynonenal), restored GSH levels, upregulated GPX4, SLC7A11, and ferritin heavy chain 1, as well as downregulated transferrin receptor 1, thereby attenuating renal injury. Notably, the Nrf2 inhibitor ML385 significantly weakens the anti-ferroptotic effects of quercetin, suggesting that Nrf2-mediated regulation of ferroptosis may be a key mechanism underlying its renoprotective actions [49]. Additionally, fisetin promotes nuclear translocation of Nrf2 and activates the Nrf2/HO-1/GPX4 antioxidant signaling pathway, consequently inhibiting ferroptosis in podocytes [55]. Collectively, current evidence supports that ferroptosis is a key mechanism underlying the renoprotective effects of flavonoids in DN, suggesting that targeting ferroptosis-related signaling networks may provide novel therapeutic strategies for the prevention and treatment of DN.

**FIGURE 8:**
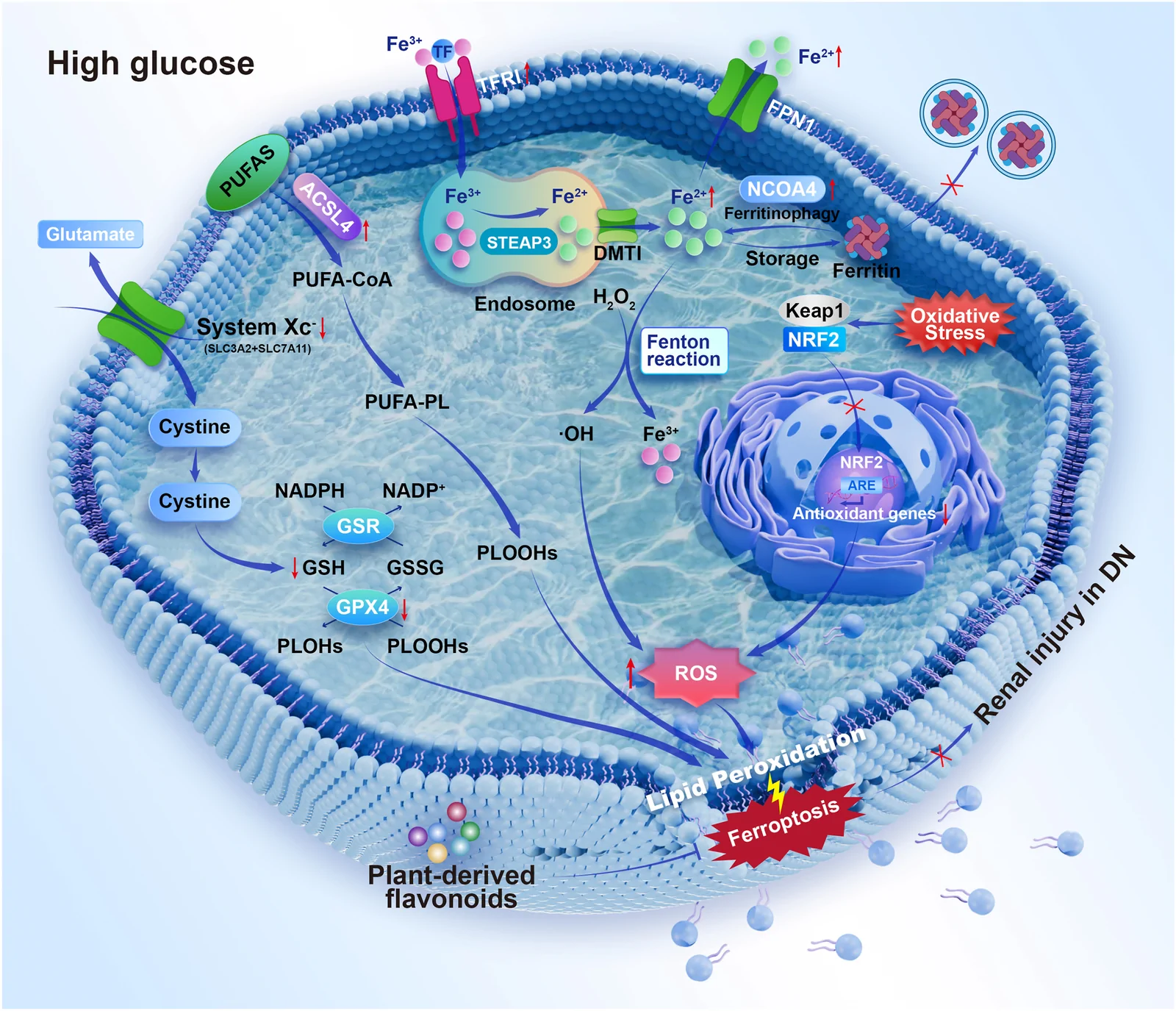
Ferroptosis-regulating mechanisms of flavonoids in DN. Flavonoids ameliorate diabetic nephropathy by inhibiting ferroptosis through modulation of iron homeostasis, restoration of the GSH/GPX4 antioxidant system, and activation of Nrf2-related signaling pathways, thereby reducing renal cellular injury in DN. ACSL4, acyl-CoA synthetase long-chain family member 4; DMT1, divalent metal transporter 1; FPN1, ferroportin 1; GSR, glutathione-disulfide reductase; GSSG, glutathione disulfide; NADP^+^, oxidized nicotinamide adenine dinucleotide phosphate; NADPH, reduced nicotinamide adenine dinucleotide phosphate; NCOA4, nuclear receptor coactivator 4; PLOH, phospholipid hydroperoxide; PLOOH, phospholipid hydroperoxide; PUFA-CoA, polyunsaturated fatty acyl-CoA; PUFA-PL, polyunsaturated phospholipids; SLC3A2, solute carrier family 3 member 2; SLC7A11, solute carrier family 7 member 11; STEAP3, six-transmembrane epithelial antigen of prostate 3; system Xc^-^, cystine/glutamate antiporter system Xc^-^; TF, transferrin; TFR1, transferrin receptor 1.

#### Effects of flavonoids on autophagy regulation in diabetic nephropathy

Autophagy is an evolutionarily conserved cellular degradation and recycling process that removes damaged cellular components via the lysosomal pathway and recycles degradation products to maintain cellular homeostasis [106]. Dysregulation of autophagy has been implicated in the development and progression of various renal diseases, including DN, and is recognized as an important pathogenic mechanism [107]. Notably, multiple flavonoids have been demonstrated to alleviate renal injury in DN, at least in part, through the modulation of autophagy (Figure 9). Formononetin has been reported to inhibit phosphorylation-mediated activation of the PI3K/AKT/mechanistic target of rapamycin (mTOR) signaling pathway, thereby relieving mTOR-mediated suppression of autophagy initiation and restoring autophagic function, ultimately alleviating renal injury in *db*/*db* mice with DN [54]. Similarly, in STZ-induced DN mice and high glucose-stimulated HK-2 cells, wogonin enhances autophagic activity by inhibiting phosphorylation of the PI3K/AKT/mTOR signaling pathway, as evidenced by an increased LC3-II/LC3-I ratio, upregulation of Beclin-1 and autophagy-related protein 7, and downregulation of p62, ultimately attenuating renal tubular epithelial cell injury [71]. Furthermore, rutin has been shown to inhibit histone deacetylase 1 via suppression of the PI3K/AKT/mTOR signaling pathway, thereby enhancing autophagic activity in *db*/*db* mice and high glucose-stimulated glomerular endothelial cells, as evidenced by upregulation of Beclin-1 and the LC3-II/LC3-I ratio [60]. This autophagic activation contributes to the attenuation of endothelial-to-mesenchymal transition. The above findings indicate that enhanced autophagic activity is associated with the alleviation of functional impairment in renal tubular epithelial cells and glomerular endothelial cells in DN, suggesting that flavonoids may confer renal protection by modulating the PI3K/AKT/mTOR–autophagy axis. Notably, kaempferol promotes AMP-activated protein kinase phosphorylation while inhibiting mTOR phosphorylation, thereby promoting autophagy [46]. This is evidenced by increased expression of autophagy-related markers and downregulation of p62, thereby potentially enhancing its renoprotective effects. In addition, kaempferol has been found to activate PTEN-induced kinase 1/E3 ubiquitin-protein ligase parkin–dependent mitophagy, facilitating the clearance of damaged mitochondria and enhancing autophagic flux, thereby alleviating renal tubular epithelial cell injury and improving renal function [47]. In summary, the amelioration of autophagy impairment may represent one of the key mechanisms by which flavonoids alleviate renal injury in DN, indicating that autophagy-related signaling pathways are potential therapeutic targets.

**FIGURE 9:**
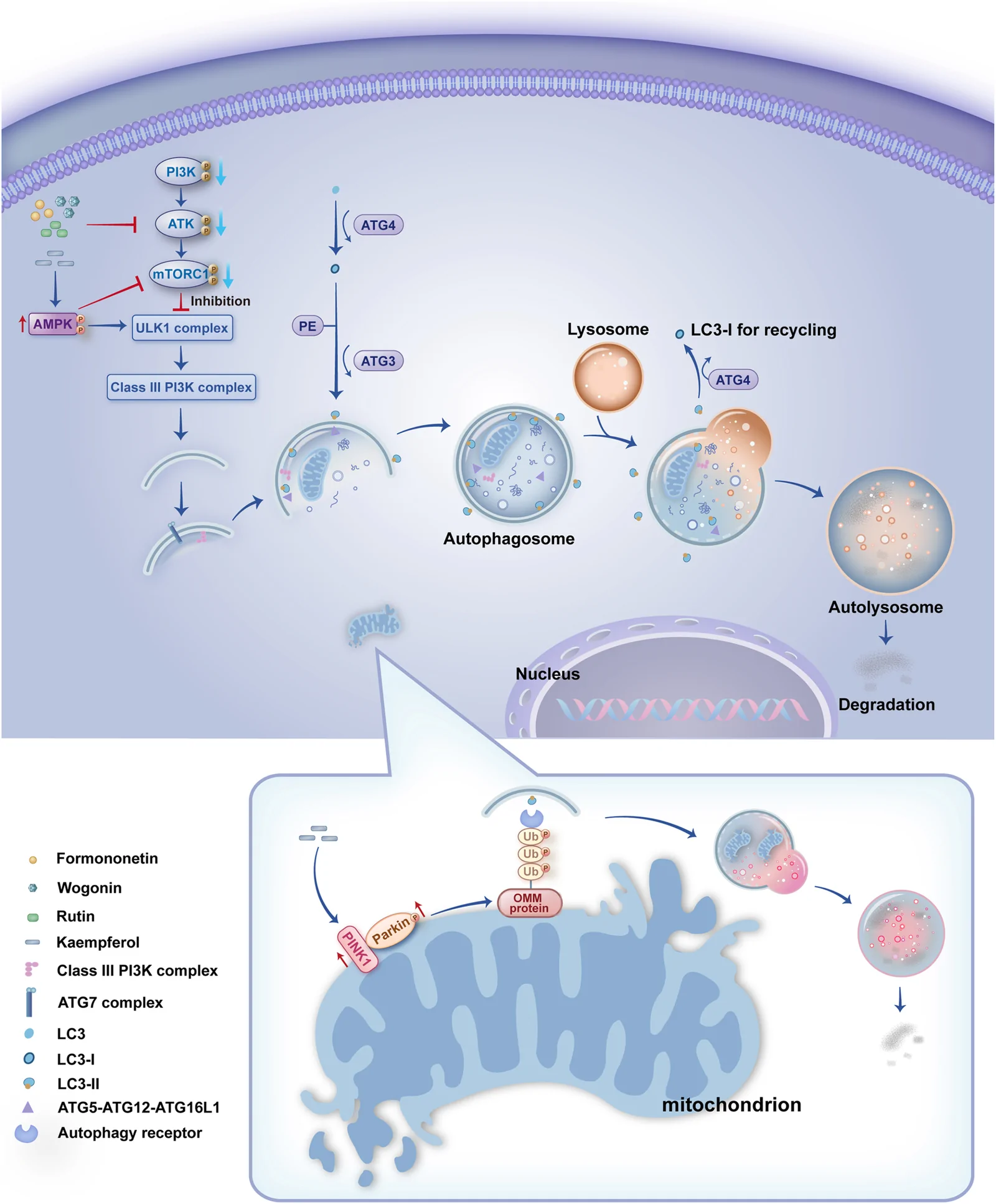
Autophagy-regulating mechanisms of flavonoids in DN. Flavonoids enhance autophagy by modulating the PI3K/AKT/mTOR axis and AMPK signaling, while also activating PINK1/Parkin-dependent mitophagy, which may contribute to attenuating renal injury in DN. AMPK, AMP-activated protein kinase; ATG3, autophagy-related protein 3; ATG4, autophagy-related protein 4; ATG5-ATG12-ATG16L1, autophagy-related protein 5-12-16-like 1 complex; mTOR, mechanistic target of rapamycin; mTORC1, mechanistic target of rapamycin complex 1; OMM protein, outer mitochondrial membrane protein; Parkin, E3 ubiquitin-protein ligase Parkin; PE, phosphatidylethanolamine; PINK1, PTEN-induced kinase 1; ULK1, Unc-51-like kinase 1.

#### Effects of flavonoids on microRNAs regulation in diabetic nephropathy

MicroRNAs (miRNAs) are a class of noncoding RNAs ∼18–22 nucleotides in length that primarily mediate post-transcriptional gene silencing by promoting target mRNA degradation or inhibiting translation, thereby regulating a wide range of biological processes [[108], [109], [110]]. A growing body of evidence suggests that dysregulated miRNA expression is critically involved in the pathogenesis of DN, making miRNAs promising therapeutic targets [[111], [112], [113]]. Recent studies have shown that multiple flavonoids exert protective effects in DN by modulating specific miRNAs and their downstream signaling pathways. For example, dihydromyricetin downregulates microRNA-155-5p, thereby upregulating its target gene PTEN, leading to enhanced autophagy and attenuation of renal tubulointerstitial fibrosis [91]. Zhou et al. [56] demonstrated that hyperoside upregulates microRNA-499e-5p (miR-499e-5p), which directly targets and suppresses adenomatous polyposis coli (APC), thereby reducing ECM deposition, inflammatory responses, and apoptosis in both *db*/*db* mice and high glucose-stimulated podocytes. Functional rescue experiments further confirmed that inhibition of miR-499e-5p or overexpression of APC attenuates or even reverses these protective effects, suggesting that the miR-499e-5p/APC axis plays a pivotal role in the renoprotective effects of hyperoside. Wan et al. [53] found that quercetin upregulates microRNA-485-5p, which directly binds to and negatively regulates Yes-associated protein 1, thereby suppressing high glucose-induced mesangial cell hyperproliferation and alleviating inflammatory responses and oxidative stress. Additionally, in HFD/STZ-induced DN rats, as well as high glucose-treated HK-2 and NRK-49F cells, icariin exerts androgen-like effects by activating the androgen receptor, leading to downregulation of microRNA-192-5p (miR-192-5p) [58]. The downregulation of miR-192-5p relieves its suppression of glucagon-like peptide-1 receptor, thereby restoring autophagy and attenuating renal tubulointerstitial fibrosis-related markers. Collectively, the above studies suggest that flavonoids may regulate specific miRNAs and their downstream signaling pathways, thereby ameliorating key pathological processes in DN. These findings highlight the potential of miRNA-based therapeutic strategies for the treatment of DN.

Overall, the mechanistic evidence varies considerably in depth across the studies summarized above. Many studies infer pathway involvement primarily from changes in protein expression, phosphorylation, or oxidative stress markers. Such findings support biologically plausible associations but do not necessarily establish causality. In contrast, several studies provide relatively stronger mechanistic evidence through pharmacologic perturbation or rescue experiments. For example, the antioxidant and renoprotective effects of luteolin were largely absent in Nrf2^-^/^-^ diabetic mice, supporting an Nrf2-dependent mechanism [68]. The protective effects of calycosin were partially reversed by erastin [82]. Similarly, GPX4 knockdown partially reversed the protective effects of vitexin against high glucose-induced ferroptosis [78], and the antiferroptotic effects of quercetin were attenuated by the Nrf2 inhibitor ML385 [49]. In addition, inhibition of miR-499-5p or overexpression of APC weakened the protective effects of hyperoside [56]. Therefore, the mechanisms discussed in this review should not be interpreted as being supported by equivalent levels of evidence, and those based primarily on marker-level changes require further causal validation.

### Crosstalk and potential synergy among flavonoid-regulated pathways in diabetic nephropathy

Although the mechanisms discussed above are presented separately for clarity, these pathways are closely interconnected in DN. Oxidative stress is a key nexus, as excessive ROS generation can promote inflammatory signaling, including activation of NF-κB, JAK2/STAT3, and NLRP3 [114,115], and also contribute to ER stress and ferroptotic or apoptotic injury [[116], [117], [118], [119], [120]]. Inflammatory and oxidative signals may further converge on profibrotic pathways, particularly TGF-β/Smad signaling, which promotes ECM accumulation and fibrotic progression [94,121]. Autophagy and mitophagy may further modulate this network by facilitating the removal of damaged organelles, particularly dysfunctional mitochondria, thereby helping to maintain cellular homeostasis and limit oxidative stress, inflammation, and cell death [[122], [123], [124]]. miRNAs add a regulatory layer by modulating key signaling pathways involved in DN pathogenesis, including autophagy, inflammation, oxidative stress, fibrosis, and apoptosis. [112,[125], [126], [127]]. Collectively, these interactions suggest that the renoprotective effects of flavonoids are more appropriately viewed as coordinated regulation of interconnected pathologic networks rather than modulation of isolated pathways. Such mechanistic overlap may partly explain the multitarget effects observed for several flavonoids; however, the relative contribution and causal hierarchy of individual pathways remain to be established.

## Optimization of Flavonoid Delivery and its Application in the Treatment of Diabetic Nephropathy

The bioavailability of flavonoids varies considerably among individual compounds and across subclasses and is influenced by factors such as chemical structure, glycosylation, intestinal metabolism, and gut microbiota [128]. For example, flavonols, flavanones, anthocyanins, and flavones differ in their absorption and metabolic profiles [129]. Human studies further demonstrate this variability. In subjects with an ileostomy, 70% of ingested green tea flavan-3-ols was recovered in ileal fluid, and urinary metabolites accounted for 6.8 ± 0.6% of total intake [130]. Urinary metabolite recovery was ∼7% for orange juice flavanones [131]. The relative bioavailability of cyanidin-3-O-glucoside was 12.38% [132]. For apigenin, urinary metabolite recovery was 0.5% after ingestion of the aglycone and increased to 11.2% and 34% after ingestion of parsley- and chamomile-derived glycosides, respectively [133]. These findings indicate that flavonoid bioavailability is highly variable rather than uniformly poor. Nevertheless, low bioavailability limits the clinical application of many flavonoids [134].

In recent years, the optimization of flavonoid delivery efficiency has become an important research focus for improving their therapeutic efficacy in DN. Huang et al. [135] prepared calycosin-loaded nanoliposomes using the thin-film hydration–ultrasonication method. As shown in Figure 10A, B, the nanoliposomes exhibited a spherical morphology with a mean size of ∼80 nm. Encapsulation of calycosin within nanoliposomes significantly enhanced its solubility, stability, and bioavailability. In a DN rat model, calycosin-loaded nanoliposomes markedly enhanced mitochondrial function in renal tissues, reduced ROS and MDA levels, and restored mitochondrial respiratory function, showing superior therapeutic efficacy compared with free calycosin. Similarly, Zhao et al. [136] developed chitosan-modified taxifolin liposomes (CTL) with a mean particle size of 286.68 nm and an encapsulation efficiency of 84.57%. Pharmacokinetic analysis in rats showed that CTL increased the AUC_0_–t of taxifolin by 5.14-fold compared with free taxifolin and prolonged its systemic exposure. In diabetic mice, CTL produced greater improvements in renal function and histopathological injury than free taxifolin and unmodified taxifolin liposomes, partly by suppressing the NF-κB/NLRP3/caspase-1/IL-1β signaling pathway. Tong et al. [137] synthesized a PEG-b-(PEG-g-PZLL) copolymer and successfully fabricated quercetin nanoparticle complexes (Figure 10C). Transmission electron microscopy image (Figure 10D) revealed that the complexes exhibited a spherical morphology with a mean size of ∼64 nm. The encapsulation efficiency of the quercetin nanoparticle complexes was determined to be 53.2%. In addition, this carrier extended the systemic circulation time of quercetin. In vivo experiments demonstrated that the quercetin nanoparticle complexes significantly alleviated renal pathological injury and improved renal function in DN rats, downregulated intercellular adhesion molecule-1 expression, and inhibited inflammatory cell infiltration, showing superior therapeutic efficacy compared with free quercetin. Moreover, Zheng et al. [138] prepared a kidney-targeting baicalin–lysozyme (BAI-LZM) conjugate via chemical synthesis. In DN rats, BAI-LZM alleviated renal inflammatory responses and fibrosis by inhibiting multiple signaling pathways, including NF-κB, insulin-like growth factor-1/p38 MAPK, and TGF-β/Smad3. Moreover, BAI-LZM exhibited superior therapeutic efficacy compared with baicalin alone.

**FIGURE 10:**
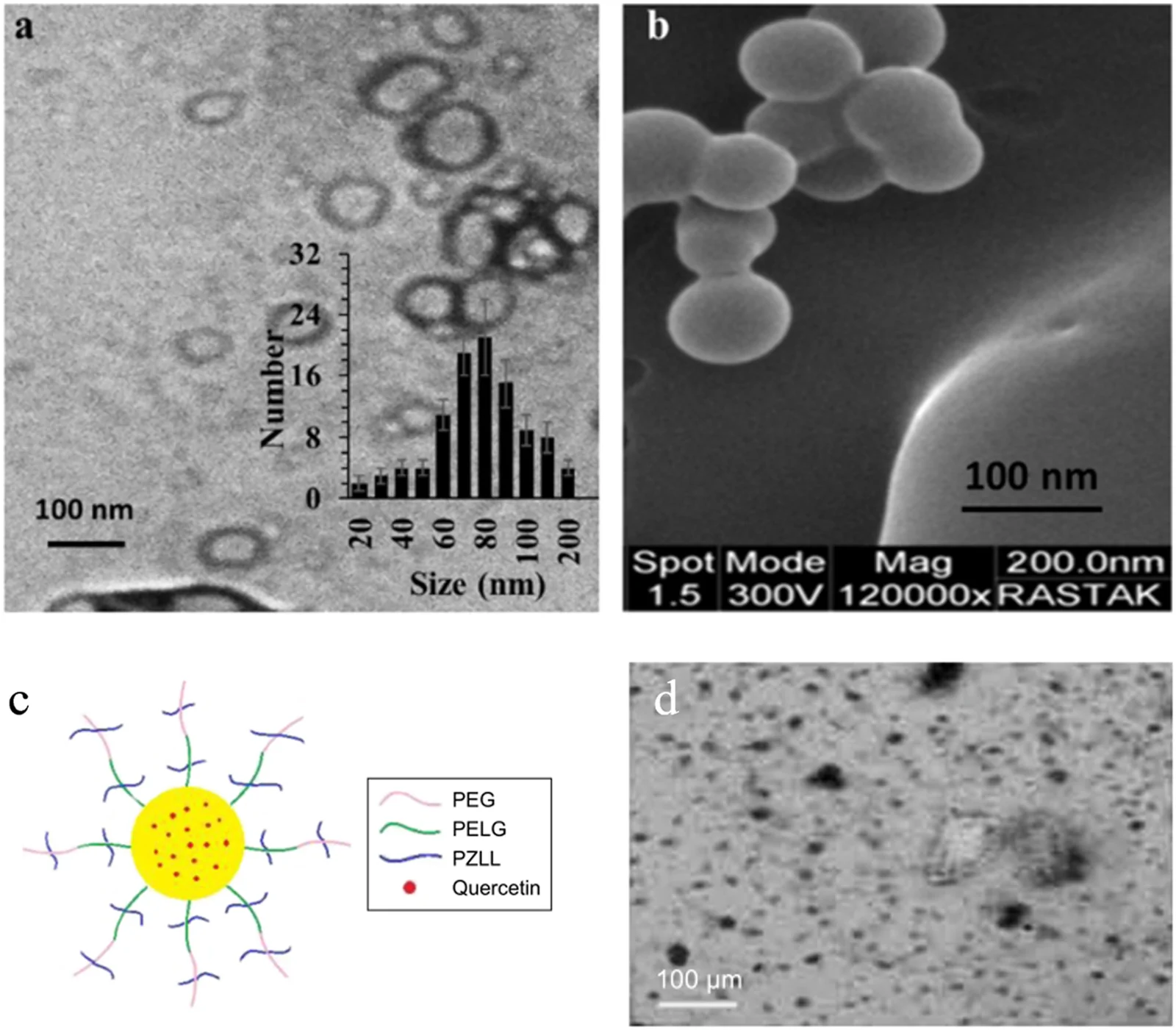
Transmission electron microscopy (TEM) image (A) and scanning electron microscopy image (B) of calycosin-loaded nanoliposomes. Schematic structure (C) and TEM image (D) of quercetin/PEG-b-(PEG-g-PZLL). (A, B) Adapted with permission from reference [138], Copyright © 2021 Springer Nature, under the terms of the Creative Commons Attribution 4.0 International License (CC BY 4.0). (C, D) Adapted with permission from reference [140], Copyright © 2017 Dove Medical Press Limited, under the terms of the Creative Commons Attribution Non-Commercial 3.0 License (CC BY-NC 3.0).

Although direct clinical evidence supporting optimized flavonoid delivery in patients with DN remains limited, human pharmacokinetic studies provide preliminary support for improving flavonoid bioavailability through formulation strategies. Riva et al. [139] evaluated a phospholipid-based quercetin formulation (Quercetin Phytosome®) in healthy volunteers and reported substantially enhanced oral absorption compared with unformulated quercetin. However, whether such pharmacokinetic improvements translate into therapeutic benefits in patients with DN remains unknown. Taken together, preclinical studies suggest that nanocarrier-based and targeted delivery strategies improve flavonoid solubility, stability, bioavailability, or renal targeting, with enhanced renoprotective effects in experimental DN models. Human pharmacokinetic evidence further indicates that a formulation-based strategy can enhance systemic exposure to quercetin. However, the clinical efficacy and long-term safety of optimized flavonoid delivery strategies in patients with DN remain to be established in well-designed clinical studies.

## Nutritional and Translational Considerations

Notably, the experimental evidence summarized in this review should be distinguished from habitual dietary flavonoid exposure. Many of the included mechanistic studies evaluated purified flavonoids in animal models at pharmacologic doses, most commonly ranging from 5 to 200 mg/kg/d (Supplemental Table 1). By comparison, the mean habitual intake of total flavonoids in a middle-aged and elderly Chinese population was ∼225 mg/d, corresponding to ∼3.75 mg/kg/d for a 60-kg adult [140]. Human randomized controlled trials have evaluated purified anthocyanins at 320 mg/d and quercetin at 730 mg/d, corresponding to 5.33 and 12.17 mg/kg/d, respectively, for a 60-kg adult [141,142]. Thus, although the lower end of the animal dose range overlaps with human supplemental doses, many animal studies, particularly those using tens to hundreds of mg/kg/d, employed doses considerably higher than habitual dietary intake and clinically evaluated supplemental doses. These simple body-weight-normalized comparisons provide only general dose context and do not establish dose equivalence between animals and humans [143]. Accordingly, these preclinical findings should not be directly extrapolated to the effects of habitual consumption of flavonoid-rich foods. After dietary intake, flavonoids undergo extensive absorption-related metabolism, and the parent compounds used experimentally may not be the major molecular forms present in the systemic circulation [144]. Consequently, bioavailability, metabolism, physiologic achievable circulating concentrations, tissue distribution, and the biologic activity of circulating metabolites are important considerations when evaluating the nutritional and translational relevance of flavonoids [[145], [146], [147]]. Future studies should further evaluate the long-term safety of sustained pharmacologic dose administration of isolated flavonoids, as well as their potential interactions with concomitant medications. Because patients with DN generally receive glucose-lowering and renoprotective therapies, including RAAS inhibitors and SGLT2 inhibitors [13,14], isolated flavonoids should currently be considered potential adjunctive candidates rather than alternatives to evidence-based therapy. Thus, dietary flavonoid intake and pharmacologic administration of isolated flavonoids should be regarded as distinct forms of exposure when interpreting the current evidence.

The gut microbiota represents an additional factor influencing flavonoid metabolism and bioavailability [148,149]. The majority of unabsorbed flavonoids can be biotransformed by gut microbiota in the large intestine, producing diverse metabolites with distinct biologic properties [150]. Naturally occurring flavonoids are predominantly present in plants as O- or C-glycosides, with O-glycosides representing the major form [151,152]. Flavonoid O-glycosides undergo hydrolysis of their glycosidic bonds by bacterial glycosidases, such as β-glucosidases and α-L-rhamnosidases, resulting in the removal of sugar moieties and the release of the corresponding aglycones [153]. In contrast, flavonoid C-glycosides are generally more resistant to hydrolysis, although certain colonic bacteria can cleave their C-C glycosidic bonds [154]. Various members of the gut microbiota belonging to Bifidobacteriaceae, Lactobacillaceae, Lachnospiraceae, and *Enterococcus* have been shown to hydrolyze flavonoid glycosides [[155], [156], [157], [158]]. The released aglycones can be further catabolized by the colon microbiota into lower-molecular-weight phenolic metabolites, including derivatives of phenylpropionic, phenylacetic, and benzoic acids with varying degrees of hydroxylation [159]. These metabolites can be more readily absorbed from the colon, thereby enhancing the overall bioavailability of dietary flavonoids [160]. Conversely, flavonoids can also modulate gut microbial composition and metabolic activity, suggesting a bidirectional interaction between flavonoids and the intestinal microbiota [[161], [162], [163]]. Specifically, flavonoids and their metabolites may exert selective antimicrobial and prebiotic-like effects, promoting beneficial and short-chain fatty acid-producing bacteria while suppressing potentially harmful taxa, with consequent changes in short-chain fatty acid and bile acid metabolism [162]. Given the emerging involvement of gut microbiota dysbiosis and the gut–kidney axis in diabetic kidney disease [164,165], this interaction may represent an additional link between flavonoid exposure and renal outcomes. However, direct evidence demonstrating that microbiota-mediated flavonoid metabolism contributes to renal protection in patients with DN remains limited, and this mechanism should currently be regarded as promising but preliminary.

To date, most evidence supporting the renoprotective effects and molecular mechanisms of individual flavonoids is derived from in vitro and animal studies. A preliminary clinical study evaluated quercetin in combination with dasatinib in individuals with DN; however, because the intervention combined both agents, the observed effects cannot be attributed specifically to quercetin [166]. Moreover, the molecular pathways identified in preclinical models have not yet been systematically validated in patients with DN. Therefore, well-designed clinical studies of individual flavonoids, incorporating pharmacokinetic and mechanistic biomarkers alongside renal outcomes, are needed to determine whether the effects observed experimentally are translatable to humans.

## Conclusion and Perspectives

Flavonoids have been shown to exert renoprotective effects in DN through multiple mechanisms, including anti-inflammatory, antioxidant, and antifibrotic activities, promotion of autophagy, inhibition of apoptosis and ferroptosis, and regulation of miRNAs. However, the mechanistic evidence should not be regarded as equally established, particularly where the proposed pathways are supported mainly by individual studies or marker-level observations. Although this review categorizes the relevant mechanisms into separate sections for clarity, these biologic processes are often interrelated and act synergistically. In addition, this review primarily summarizes the key mechanisms that have been relatively well characterized to date, although additional pathways may remain insufficiently elucidated. Several flavonoids, including quercetin, kaempferol, and puerarin, appear to be supported by a broader preclinical evidence base. Collectively, these 3 flavonoids have been reported to exert renoprotective effects in experimental DN models through diverse mechanisms involving the modulation of inflammation, oxidative stress, fibrosis, autophagy, apoptosis, and ferroptosis. Flavonoids with repeated evidence of preclinical efficacy and improved delivery strategies, such as quercetin and calycosin, may merit further investigation.

However, the clinical efficacy in DN remains unestablished, and caution is warranted when extrapolating findings obtained with purified flavonoids under experimental exposure conditions to habitual dietary consumption of flavonoid-rich foods. Several limitations should also be noted. First, this review focused on pure flavonoids and excluded flavonoid-rich plant extracts; therefore, the applicability of these findings to crude extracts or dietary sources requires further validation. Second, most of the included studies were preclinical (in vitro or animal models), and the doses and concentrations used may not reflect achievable exposures via diet or supplementation in humans. Accordingly, the reported molecular effects should be interpreted as evidence of preclinical mechanistic potential rather than established clinical efficacy. Third, no formal risk-of-bias assessment or certainty-of-evidence grading was performed, precluding systematic comparison of methodological robustness or formal grading of overall evidence certainty. Fourth, although the included compounds encompassed multiple flavonoid subclasses, subclass-specific differences in bioavailability, metabolism, and mechanisms of action were not systematically evaluated. This limits the extent to which the present conclusions can be generalized across structurally distinct flavonoids.

Future clinical research should move beyond efficacy assessment alone and incorporate mechanistic and exposure-related endpoints. Controlled intervention studies should evaluate renal outcomes together with predefined biomarkers of oxidative stress or inflammation after administration of individual flavonoids. Dose-ranging and pharmacokinetic studies are also needed to establish physiologically achievable exposures and dose–response relationships. In addition, mechanistic studies in human cohorts integrating gut microbiome profiles, circulating flavonoid metabolites, and renal or inflammatory outcomes may help clarify whether microbiota-related differences contribute to interindividual variability in flavonoid metabolism and biologic responses.

## Author contributions

The authors’ responsibilities were as follows – ET: performed the literature synthesis and wrote the paper; SH, HZ: conducted the literature search and prepared all figures and tables; WL, ZZ: designed and supervised the study; and all authors: have read and approved the final version.

## Funding

This study was supported by the National Natural Science Foundation of China (grant no. 82373559) and the Guangdong Basic and Applied Basic Research Foundation (grant no. 2024A1515012255).

## Conflict of interest

The authors report no conflicts of interest.

## References

[1] Liu J., Tan X., Fang X., Ge X., Xu L., Long Y. (2026). Deubiquitinating enzymes as therapeutic targets in diabetic nephropathy. Trends Pharmacol. Sci..

[2] Zhang L., Jiang L., Xu R., Zhang X., Zhang B., Yue R. (2025). Epidemiological research on diabetic nephropathy at global, regional, and national levels from 1990 to 2021: an analysis derived from the global burden of disease 2021 study. Front. Endocrinol (Lausanne).

[3] Wang G., Ouyang J., Li S., Wang H., Lian B., Liu Z. (2019). The analysis of risk factors for diabetic nephropathy progression and the construction of a prognostic database for chronic kidney diseases. J. Transl. Med..

[4] de Boer I.H., Rue T.C., Hall Y.N., Heagerty P.J., Weiss N.S., Himmelfarb J. (2011). Temporal trends in the prevalence of diabetic kidney disease in the United States. JAMA..

[5] Sun H., Saeedi P., Karuranga S., Pinkepank M., Ogurtsova K., Duncan B.B. (2022). IDF Diabetes Atlas: global, regional and country-level diabetes prevalence estimates for 2021 and projections for 2045. Diabetes Res. Clin. Pract..

[6] Alicic R., Nicholas S.B. (2022). Diabetic kidney disease back in focus: management field guide for health care professionals in the 21st century. Mayo Clin. Proc..

[7] Tervaert T.W., Mooyaart A.L., Amann K., Cohen A.H., Cook H.T., Drachenberg C.B. (2010). Pathologic classification of diabetic nephropathy. J. Am. Soc. Nephrol..

[8] Toyoda M., Najafian B., Kim Y., Caramori M.L., Mauer M. (2007). Podocyte detachment and reduced glomerular capillary endothelial fenestration in human type 1 diabetic nephropathy. Diabetes.

[9] Zhang J., Yang S., Li H., Chen F., Shi J. (2017). Naringin ameliorates diabetic nephropathy by inhibiting NADPH oxidase 4. Eur. J. Pharmacol..

[10] Selby N.M., Taal M.W. (2020). An updated overview of diabetic nephropathy: diagnosis, prognosis, treatment goals and latest guidelines. Diabetes Obes. Metab..

[11] Gross J.L., de Azevedo M.J., Silveiro S.P., Canani L.H., Caramori M.L., Zelmanovitz T. (2005). Diabetic nephropathy: diagnosis, prevention, and treatment. Diabetes Care.

[12] Umanath K., Lewis J.B. (2018). Update on diabetic nephropathy: core curriculum 2018. Am. J. Kidney Dis..

[13] DeFronzo R.A., Reeves W.B., Awad A.S. (2021). Pathophysiology of diabetic kidney disease: impact of SGLT2 inhibitors. Nat. Rev. Nephrol..

[14] Martinez Leon V., Hilburg R., Susztak K. (2026). Mechanisms of diabetic kidney disease and established and emerging treatments. Nat. Rev. Endocrinol..

[15] Tuttle K.R., Agarwal R., Alpers C.E., Bakris G.L., Brosius F.C., Kolkhof P. (2022). Molecular mechanisms and therapeutic targets for diabetic kidney disease. Kidney Int.

[16] Ding T., Wang S., Zhang X., Zai W., Fan J., Chen W. (2018). Kidney protection effects of dihydroquercetin on diabetic nephropathy through suppressing ROS and NLRP3 inflammasome. Phytomedicine.

[17] Feng Q., Yu X., Xie J., Liu F., Zhang X., Li S. (2025). Phillygenin improves diabetic nephropathy by inhibiting inflammation and apoptosis via regulating TLR4/MyD88/NF-κB and PI3K/AKT/GSK3β signaling pathways. Phytomedicine.

[18] Ai S., Tao J., Wang Y., Zhou J., Liu W., Li X. (2025). Paeonol ameliorates diabetic nephropathy by promoting TFEB-mediated lysosome biogenesis and lipophagy. Phytomedicine.

[19] Wu Y., Cao Y., Feng L., Yan Y., Ye H., Tang K. (2025). The natural compound stachyose targets SGLT2-mediated metabolic reprogramming to ameliorate diabetic kidney disease. Phytomedicine.

[20] Hu Q., Qu C., Xiao X., Zhang W., Jiang Y., Wu Z. (2021). Flavonoids on diabetic nephropathy: advances and therapeutic opportunities. Chin. Med..

[21] Alshehri A.S. (2023). Kaempferol attenuates diabetic nephropathy in streptozotocin-induced diabetic rats by a hypoglycaemic effect and concomitant activation of the Nrf-2/Ho-1/antioxidants axis. Arch. Physiol. Biochem..

[22] Gao F., He X., Liang S., Liu S., Liu H., He Q. (2018). Quercetin ameliorates podocyte injury via inhibition of oxidative stress and the TGF-β1/Smad pathway in DN rats. RSC Adv.

[23] Li D., Li H., Shi B., Li C. (2025). Wogonin Alleviates Streptozotocin-stimulated diabetic nephropathy through its antidiabetic, antidyslipidemic, and antioxidative effects in rats. Pharmacogn. Mag..

[24] Zhu Q.Q., Yang X.Y., Zhang X.J., Yu C.J., Pang Q.Q., Huang Y.W. (2020). EGCG targeting Notch to attenuate renal fibrosis via inhibition of TGFβ/Smad3 signaling pathway activation in streptozotocin-induced diabetic mice. Food Funct.

[25] Wang H., Li W. (2024). Puerarin alleviates the high glucose-induced oxidative stress via the RAGE/PKC/NOX4 axis in renal mesangial cells. J. Toxicol. Sci..

[26] Wu J., Li K., Zhou M., Gao H., Wang W., Xiao W. (2024). Natural compounds improve diabetic nephropathy by regulating the TLR4 signaling pathway. J. Pharm. Anal..

[27] Amanat M., Lal K., Singh T.G., Singh R. (2025). Molecular insights of diabetic nephropathy and chemical constituents-based treatment approach. Phytochem. Rev..

[28] Gao J.W., Wu Y.Z., Cai X.D., Zhao H.L., Xing J. (2025). Therapeutic potential of natural medicines in diabetic kidney disease: restoring lipid homeostasis via lipophagy modulation. Front. Pharmacol..

[29] Kanwar Y.S., Sun L., Xie P., Liu F.Y., Chen S. (2011). A glimpse of various pathogenetic mechanisms of diabetic nephropathy. Annu. Rev. Pathol..

[30] Hostetter T.H., Troy J.L., Brenner B.M. (1981). Glomerular hemodynamics in experimental diabetes mellitus. Kidney Int.

[31] Tonneijck L., Muskiet M.H., Smits M.M., van Bommel E.J., Heerspink H.J., van Raalte D.H. (2017). Glomerular hyperfiltration in diabetes: mechanisms, clinical significance, and treatment. J. Am. Soc. Nephrol..

[32] Ikenaga H., Bast J.P., Fallet R.W., Carmines P.K. (2000). Exaggerated impact of ATP-sensitive K(+) channels on afferent arteriolar diameter in diabetes mellitus. J. Am. Soc. Nephrol..

[33] Yoo T.H., Li J.J., Kim J.J., Jung D.S., Kwak S.J., Ryu D.R. (2007). Activation of the renin-angiotensin system within podocytes in diabetes. Kidney Int.

[34] Singh R., Singh A.K., Leehey D.J. (2005). A novel mechanism for angiotensin II formation in streptozotocin-diabetic rat glomeruli. Am. J. Physiol. Renal Physiol..

[35] Mohandes S., Doke T., Hu H., Mukhi D., Dhillon P., Susztak K. (2023). Molecular pathways that drive diabetic kidney disease. J. Clin. Invest..

[36] Anders H.J., Huber T.B., Isermann B., Schiffer M. (2018). CKD in diabetes: diabetic kidney disease versus nondiabetic kidney disease. Nat. Rev. Nephrol..

[37] Ilyas Z., Chaiban J.T., Krikorian A. (2017). Novel insights into the pathophysiology and clinical aspects of diabetic nephropathy. Rev. Endocr. Metab. Disord..

[38] Sankrityayan H., Kulkarni Y.A., Gaikwad A.B. (2019). Diabetic nephropathy: the regulatory interplay between epigenetics and microRNAs. Pharmacol. Res..

[39] Yang R., Hou Q., Chen R., Ma Z., Jiang S., Liu Z. (2025). Protein UHRF1-mediated dual dysregulation of molecular chaperone GRP78 induces endoplasmic reticulum stress and aggravates diabetic nephropathy. Kidney Int.

[40] Higgins G.C., Coughlan M.T. (2014). Mitochondrial dysfunction and mitophagy: the beginning and end to diabetic nephropathy?. Br. J. Pharmacol..

[41] Kotik M., Kulik N., Valentová K. (2023). Flavonoids as aglycones in retaining glycosidase-catalyzed reactions: prospects for green chemistry. J. Agric. Food Chem..

[42] Shen N., Wang T., Gan Q., Liu S., Wang L., Jin B. (2022). Plant flavonoids: classification, distribution, biosynthesis, and antioxidant activity. Food Chem.

[43] Rozmer Z., Perjési P. (2016). Naturally occurring chalcones and their biological activities. Phytochem. Rev..

[44] Carvalho M.T.B., Araújo-Filho H.G., Barreto A.S., Quintans-Júnior L.J., Quintans J.S.S., Barreto R.S.S. (2021). Wound healing properties of flavonoids: a systematic review highlighting the mechanisms of action. Phytomedicine.

[45] Luo W., Chen X., Ye L., Chen X., Jia W., Zhao Y. (2021). Kaempferol attenuates streptozotocin-induced diabetic nephropathy by downregulating TRAF6 expression: the role of TRAF6 in diabetic nephropathy. J. Ethnopharmacol..

[46] Sheng H., Zhang D., Zhang J., Zhang Y., Lu Z., Mao W. (2022). Kaempferol attenuated diabetic nephropathy by reducing apoptosis and promoting autophagy through AMPK/mTOR pathways. Front. Med. (Lausanne).

[47] Xia C., Zhang J., Chen H., Jiang W., Zhou S., Zheng H. (2025). Kaempferol improves mitochondrial homeostasis via mitochondrial dynamics and mitophagy in diabetic kidney disease. Int. Immunopharmacol..

[48] Feng Q., Yang Y., Qiao Y., Zheng Y., Yu X., Liu F. (2023). Quercetin ameliorates diabetic kidney injury by inhibiting ferroptosis via activating Nrf2/HO-1 signaling pathway. Am. J. Chin. Med..

[49] Zhang L., Wang X., Chang L., Ren Y., Sui M., Fu Y. (2024). Quercetin improves diabetic kidney disease by inhibiting ferroptosis and regulating the Nrf2 in streptozotocin-induced diabetic rats. Ren. Fail..

[50] Zhao X., Wang S., He X., Wei W., Huang K. (2024). Quercetin prevents the USP22-Snail1 signaling pathway to ameliorate diabetic tubulointerstitial fibrosis. Food Funct.

[51] Liu F., Feng Q., Yang M., Yang Y., Nie J., Wang S. (2024). Quercetin prevented diabetic nephropathy by inhibiting renal tubular epithelial cell apoptosis via the PI3K/AKT pathway. Phytother. Res..

[52] Liu Y., Li Y., Xu L., Shi J., Yu X., Wang X. (2021). Quercetin attenuates podocyte apoptosis of diabetic nephropathy through targeting EGFR signaling. Front. Pharmacol..

[53] Wan H., Wang Y., Pan Q., Chen X., Chen S., Li X. (2022). Quercetin attenuates the proliferation, inflammation, and oxidative stress of high glucose-induced human mesangial cells by regulating the miR-485–5p/YAP1 pathway. Int. J. Immunopathol. Pharmacol..

[54] Song S., Zhou X., Huang L., Yu J. (2025). Nephroprotective effects of formononetin in diabetic kidney disease: mechanistic insights and therapeutic potential. Am. J. Chin. Med..

[55] Qian X., Lin S., Li J., Jia C., Luo Y., Fan R. (2023). Fisetin ameliorates diabetic nephropathy-induced podocyte injury by modulating Nrf2/HO-1/GPX4 signaling pathway. Evid. Based Complement. Alternat. Med..

[56] Zhou J., Zhang S., Sun X., Lou Y., Bao J., Yu J. (2021). Hyperoside ameliorates diabetic nephropathy induced by STZ via targeting the miR-499–5p/APC axis. J. Pharmacol. Sci..

[57] Qiao C., Ye W., Li S., Wang H., Ding X. (2018). Icariin modulates mitochondrial function and apoptosis in high glucose-induced glomerular podocytes through G protein-coupled estrogen receptors. Mol. Cell Endocrinol..

[58] Jia Z., Wang K., Zhang Y., Duan Y., Xiao K., Liu S. (2021). Icariin ameliorates diabetic renal tubulointerstitial fibrosis by restoring autophagy via regulation of the miR-192–5p/GLP-1R pathway. Front. Pharmacol..

[59] Xuan C., Chen D., Zhang S., Li C., Fang Q., Chen D. (2025). Isoquercitrin alleviates diabetic nephropathy by inhibiting STAT3 phosphorylation and dimerization. Adv. Sci..

[60] Dong R., Zhang X., Liu Y., Zhao T., Sun Z., Liu P. (2023). Rutin alleviates EndMT by restoring autophagy through inhibiting HDAC1 via PI3K/AKT/mTOR pathway in diabetic kidney disease. Phytomedicine.

[61] Zhang X., Huo Z., Jia X., Xiong Y., Li B., Zhang L. (2024). (+)-Catechin ameliorates diabetic nephropathy injury by inhibiting endoplasmic reticulum stress-related NLRP3-mediated inflammation. Food Funct.

[62] Ni Y., Zhu D., Chen C., Wang F., Miu Y., Zhang W. (2024). (+)-Catechins play a protective role in diabetic kidney disease by alleviating EMT through multiple pathways. Mol. Nutr. Food Res..

[63] Mohan T., Narasimhan K.K.S., Ravi D.B., Velusamy P., Chandrasekar N., Chakrapani L.N. (2020). Role of Nrf2 dysfunction in the pathogenesis of diabetic nephropathy: therapeutic prospect of epigallocatechin-3-gallate. Free Radic. Biol. Med..

[64] Mohan T., Velusamy P., Chakrapani L.N., Srinivasan A.K., Singh A., Johnson T. (2017). Impact of EGCG supplementation on the progression of diabetic nephropathy in rats: an insight into fibrosis and apoptosis. J. Agric. Food Chem..

[65] Álvarez Cilleros D., López-Oliva M.E., Martín M., Ramos S. (2020). (-)-Epicatechin and the colonic metabolite 2,3-dihydroxybenzoic acid protect against high glucose and lipopolysaccharide-induced inflammation in renal proximal tubular cells through NOX-4/p38 signalling. Food Funct.

[66] Malik S., Suchal K., Khan S.I., Bhatia J., Kishore K., Dinda A.K. (2017). Apigenin ameliorates streptozotocin-induced diabetic nephropathy in rats via MAPK-NF-κB-TNF-α and TGF-β1-MAPK-fibronectin pathways. Am. J. Physiol. Renal Physiol..

[67] Kang M.K., Park S.H., Kim Y.H., Lee E.J., Antika L.D., Kim D.Y. (2017). Chrysin ameliorates podocyte injury and slit diaphragm protein loss via inhibition of the PERK-eIF2α-ATF-CHOP pathway in diabetic mice. Acta Pharmacol. Sin..

[68] Yang Y, Chen G, Cheng X, Teng Z, Cai X, Yang J (2015). Therapeutic potential of digitoflavone on diabetic nephropathy: nuclear factor erythroid 2-related factor 2-dependent anti-oxidant and anti-inflammatory effect. Sci Rep.

[69] Kang M.K., Kim S.I., Oh S.Y., Na W., Kang Y.H. (2020). Tangeretin ameliorates glucose-induced podocyte injury through blocking epithelial to mesenchymal transition caused by oxidative stress and hypoxia. Int. J. Mol. Sci..

[70] Liu Y., Zhang M., Zeng L., Lai Y., Wu S., Su X. (2024). Wogonin upregulates SOCS3 to alleviate the injury in diabetic nephropathy by inhibiting TLR4-mediated JAK/STAT/AIM2 signaling pathway. Mol. Med..

[71] Lei L., Zhao J., Liu X.Q., Chen J., Qi X.M., Xia L.L. (2021). Wogonin alleviates kidney tubular epithelial injury in diabetic nephropathy by inhibiting PI3K/Akt/NF-κB signaling pathways. Drug Des. Devel. Ther..

[72] Zheng Z.C., Zhu W., Lei L., Liu X.Q., Wu Y.G. (2020). Wogonin ameliorates renal inflammation and fibrosis by inhibiting NF-κB and TGF-β1/Smad3 signaling pathways in diabetic nephropathy. Drug Des. Devel. Ther..

[73] Liu X.Q., Jiang L., Li Y.Y., Huang Y.B., Hu X.R., Zhu W. (2022). Wogonin protects glomerular podocytes by targeting Bcl-2-mediated autophagy and apoptosis in diabetic kidney disease. Acta Pharmacol. Sin..

[74] Li M., Zhu H.Y., Zhao S.Y., Li X.D., Tong S.M., Ma J. (2025). Baicalin alleviates lipid metabolism disorders in diabetic kidney disease via targeting FKBP51. Phytomedicine.

[75] Ma L., Wu F., Shao Q., Chen G., Xu L., Lu F. (2021). Baicalin alleviates oxidative stress and inflammation in diabetic nephropathy via Nrf2 and MAPK signaling pathway. Drug Des. Dev. Ther..

[76] Deng J., Zheng C., Hua Z., Ci H., Wang G., Chen L. (2022). Diosmin mitigates high glucose-induced endoplasmic reticulum stress through PI3K/AKT pathway in HK-2 cells. BMC Complement. Med. Ther..

[77] Huang B., Han R., Tan H., Zhu W., Li Y., Jiang F. (2024). Scutellarin ameliorates diabetic nephropathy via TGF-β1 signaling pathway. Nat. Prod. Bioprospect..

[78] Zhang S., Zhang S., Wang H., Chen Y. (2023). Vitexin ameliorated diabetic nephropathy via suppressing GPX4-mediated ferroptosis. Eur. J. Pharmacol..

[79] Li X., Zhao S., Li M., Xing X., Xie J., Wang M. (2025). Wogonoside ameliorates oxidative damage in tubular epithelial cells of diabetic nephropathy by modulating the HNF4A-NRF2 axis. Int. Immunopharmacol..

[80] Bai J., Wang Y., Zhu X., Shi J. (2019). Eriodictyol inhibits high glucose-induced extracellular matrix accumulation, oxidative stress, and inflammation in human glomerular mesangial cells. Phytother. Res..

[81] Zhou Y., Hu T., Zeng H., Lin L., Xie H., Lin R. (2025). Naringenin inhibits ferroptosis in renal tubular epithelial cells of diabetic nephropathy through SIRT1/FOXO3a signaling pathway. Drug Dev. Res..

[82] Huang D., Shen P., Wang C., Gao J., Ye C., Wu F. (2022). Calycosin plays a protective role in diabetic kidney disease through the regulation of ferroptosis. Pharm. Biol..

[83] Zhuang K., Jiang X., Liu R., Ye C., Wang Y., Wang Y. (2020). Formononetin activates the Nrf2/ARE signaling pathway via Sirt1 to improve diabetic renal fibrosis. Front. Pharmacol..

[84] Hou B., Ma P., Yang X., Zhao X., Zhang L., Zhao Y. (2024). In silico prediction and experimental validation to reveal the protective mechanism of Puerarin against excessive extracellular matrix accumulation through inhibiting ferroptosis in diabetic nephropathy. J. Ethnopharmacol..

[85] Wang L., Xie X., Chen Q., Chen Y., Xu X., Liang T. (2025). Puerarin reduces diabetic nephropathy-induced podocyte pyroptosis by modulating the SIRT1/NLRP3/caspase-1 pathway. Mol. Cell Endocrinol..

[86] Wang Y., Yang J., Chang X., Xue Y., Liu G., Zhang T. (2024). Isoliquiritigenin alleviates diabetic kidney disease via oxidative stress and the TLR4/NF-κB/NLRP3 inflammasome pathway. Mol. Nutr. Food Res..

[87] Huang X., Shi Y., Chen H., Le R., Gong X., Xu K. (2020). Isoliquiritigenin prevents hyperglycemia-induced renal injuries by inhibiting inflammation and oxidative stress via SIRT1-dependent mechanism. Cell Death Dis.

[88] Li J., Kang S.W., Kim J.L., Sung H.Y., Kwun I.S., Kang Y.H. (2010). Isoliquiritigenin entails blockade of TGF-beta1-SMAD signaling for retarding high glucose-induced mesangial matrix accumulation. J. Agric. Food Chem..

[89] Chen F., Zhu X., Sun Z., Ma Y. (2018). Astilbin inhibits high glucose-induced inflammation and extracellular matrix accumulation by suppressing the TLR4/MyD88/NF-κB pathway in rat glomerular mesangial cells. Front. Pharmacol..

[90] Dong C., Wu G., Li H., Qiao Y., Gao S. (2020). Ampelopsin inhibits high glucose-induced extracellular matrix accumulation and oxidative stress in mesangial cells through activating the Nrf2/HO-1 pathway. Phytother. Res..

[91] Guo L., Tan K., Luo Q., Bai X. (2020). Dihydromyricetin promotes autophagy and attenuates renal interstitial fibrosis by regulating miR-155–5p/PTEN signaling in diabetic nephropathy. Bosn. J. Basic Med. Sci..

[92] Zheng H.X., Qi S.S., He J., Hu C.Y., Han H., Jiang H. (2020). Cyanidin-3-glucoside from black rice ameliorates diabetic nephropathy via reducing blood glucose, suppressing oxidative stress and inflammation, and regulating transforming growth factor β1/Smad expression. J. Agric. Food Chem..

[93] Tang S.C.W., Yiu W.H. (2020). Innate immunity in diabetic kidney disease. Nat. Rev. Nephrol..

[94] Wang N., Zhang C. (2024). Oxidative stress: a culprit in the progression of diabetic kidney disease. Antioxidants (Basel).

[95] Kato M., Natarajan R. (2019). Epigenetics and epigenomics in diabetic kidney disease and metabolic memory. Nat. Rev. Nephrol..

[96] Jiang C.H., Zhang S.J., Li P., Miao H., Zhao Y.Y. (2025). Natural products targeting TGF-β/Smad signaling in renal fibrosis: multiomics-based novel molecular mechanisms and therapeutic strategies. Phytomedicine.

[97] Sisto M., Ribatti D., Lisi S. (2021). Organ fibrosis and autoimmunity: the role of inflammation in TGFβ-dependent EMT. Biomolecules.

[98] Lamouille S., Xu J., Derynck R. (2014). Molecular mechanisms of epithelial-mesenchymal transition. Nat. Rev. Mol. Cell Biol..

[99] Celik C., Lee S.Y.T., Yap W.S., Thibault G. (2023). Endoplasmic reticulum stress and lipids in health and diseases. Prog. Lipid Res..

[100] Oakes S.A., Papa F.R. (2015). The role of endoplasmic reticulum stress in human pathology. Annu. Rev. Pathol..

[101] Zhang R., Bian C., Gao J., Ren H. (2023). Endoplasmic reticulum stress in diabetic kidney disease: adaptation and apoptosis after three UPR pathways. Apoptosis.

[102] Su B., Cheng D., Chen G., Zhang S., Wang L., Wu X. (2022). Icariin attenuation of diabetic kidney disease through inhibition of endoplasmic reticulum stress via G protein-coupled estrogen receptors. J. Biomed. Nanotechnol..

[103] Dixon S.J., Olzmann J.A. (2024). The cell biology of ferroptosis. Nat. Rev. Mol. Cell Biol..

[104] Wang H., Liu D., Zheng B., Yang Y., Qiao Y., Li S. (2023). Emerging role of ferroptosis in diabetic kidney disease: molecular mechanisms and therapeutic opportunities. Int. J. Biol. Sci..

[105] Wang J., He F., Wang R., Zou F., Yan W., Cheng S. (2025). Intermodulation of endoplasmic reticulum stress and ferroptosis in diabetic nephropathy: molecular mechanisms and therapeutic potentials. Apoptosis.

[106] Ortega M.A., Fraile-Martinez O., de Leon-Oliva D., Boaru D.L., Lopez-Gonzalez L., García-Montero C. (2024). Autophagy in its (proper) context: molecular basis, biological relevance, pharmacological modulation, and lifestyle medicine. Int. J. Biol. Sci..

[107] Tang C., Livingston M.J., Liu Z., Dong Z. (2020). Autophagy in kidney homeostasis and disease. Nat. Rev. Nephrol..

[108] Zhang L., Chen T., Yin Y., Zhang C.Y., Zhang Y.L. (2019). Dietary microRNA—a novel functional component of food. Adv. Nutr..

[109] Lu T.X., Rothenberg M.E. (2018). MicroRNA. J. Allergy Clin. Immunol..

[110] Ho P.T.B., Clark I.M., Le L.T.T. (2022). MicroRNA-based diagnosis and therapy. Int. J. Mol. Sci..

[111] Kato M., Natarajan R. (2014). Diabetic nephropathy-emerging epigenetic mechanisms. Nat. Rev. Nephrol..

[112] Kato M., Natarajan R. (2015). MicroRNAs in diabetic nephropathy: functions, biomarkers, and therapeutic targets. Ann. N. Y. Acad. Sci..

[113] Zhou H., Ni W.J., Meng X.M., Tang L.Q. (2020). MicroRNAs as regulators of immune and inflammatory responses: potential therapeutic targets in diabetic nephropathy. Front. Cell Dev. Biol..

[114] Su S., Ma Z., Wu H., Xu Z., Yi H. (2023). Oxidative stress as a culprit in diabetic kidney disease. Life Sci.

[115] Wang X., Li S., Ge X., Xie T., Li S., Yuan R. (2026). Advances in understanding the NLRP3 inflammasome-mediated mechanisms and therapeutic targets in diabetic nephropathy. Int. J. Mol. Med..

[116] Zeng X., Zhang Y., Tian L., Zheng Y., Zhang J., Wu Z. (2024). Mitigation of ROS-triggered endoplasmic reticulum stress by upregulating Nrf2 retards diabetic nephropathy. Biochem. Biophys. Res. Commun..

[117] Daehn I.S., Ekperikpe U.S., Stadler K. (2023). Redox regulation in diabetic kidney disease. Am. J. Physiol. Renal Physiol..

[118] Liu F., Yang Z., Li J., Wu T., Li X., Zhao L. (2024). Targeting programmed cell death in diabetic kidney disease: from molecular mechanisms to pharmacotherapy. Mol. Med..

[119] Sifuentes-Franco S., Padilla-Tejeda D.E., Carrillo-Ibarra S., Miranda-Díaz A.G. (2018). Oxidative Stress, Apoptosis, and Mitochondrial Function in Diabetic Nephropathy. Int. J. Endocrinol..

[120] Li Q., Liao J., Chen W., Zhang K., Li H., Ma F. (2022). NAC alleviative ferroptosis in diabetic nephropathy via maintaining mitochondrial redox homeostasis through activating SIRT3-SOD2/Gpx4 pathway. Free Radic. Biol. Med..

[121] Al-Masri S., Coelho J.N., Thomas L. (2026). Molecular pathways and emerging therapeutic targets in the pathogenesis of diabetic kidney disease. Front. Physiol..

[122] Lytvyn Y., Bjornstad P., van Raalte D.H., Heerspink H.L., Cherney D.Z.I. (2020). The new biology of diabetic kidney disease-mechanisms and therapeutic implications. Endocr. Rev..

[123] Bhatia D., Choi M.E. (2023). Autophagy and mitophagy: physiological implications in kidney inflammation and diseases. Am. J. Physiol. Renal Physiol..

[124] Stanigut A.M., Tuta L., Pana C., Alexandrescu L., Suceveanu A., Blebea N.M. (2025). Autophagy and mitophagy in diabetic kidney disease—a literature review. Int. J. Mol. Sci..

[125] Szostak J., Gorący A., Durys D., Dec P., Modrzejewski A., Pawlik A. (2023). The role of MicroRNA in the pathogenesis of diabetic nephropathy. Int. J. Mol. Sci..

[126] Yang Z., Guo Z., Dong J., Sheng S., Wang Y., Yu L. (2018). miR-374a regulates inflammatory response in diabetic nephropathy by targeting MCP-1 expression. Front. Pharmacol..

[127] Al-Tantawy S.M., Eraky S.M., Eissa L.A. (2023). Promising renoprotective effect of gold nanoparticles and dapagliflozin in diabetic nephropathy via targeting miR-192 and miR-21. J. Biochem. Mol. Toxicol..

[128] Chen L., Cao H., Huang Q., Xiao J., Teng H. (2022). Absorption, metabolism and bioavailability of flavonoids: a review. Crit. Rev. Food Sci. Nutr..

[129] Manach C., Scalbert A., Morand C., Rémésy C., Jiménez L. (2004). Polyphenols: food sources and bioavailability. Am. J. Clin. Nutr..

[130] Stalmach A., Mullen W., Steiling H., Williamson G., Lean M.E.J., Crozier A. (2010). Absorption, metabolism, and excretion of green tea flavan-3-ols in humans with an ileostomy. Mol. Nutr. Food Res..

[131] Mullen W., Archeveque M.-A., Edwards C.A., Matsumoto H., Crozier A. (2008). Bioavailability and metabolism of orange juice flavanones in humans: impact of a full-fat yogurt. J. Agric. Food Chem..

[132] Czank C., Cassidy A., Zhang Q., Morrison D.J., Preston T., Kroon P.A. (2013). Human metabolism and elimination of the anthocyanin, cyanidin-3-glucoside: a ^13^C-tracer study. Am. J. Clin. Nutr..

[133] Borges G., Fong R.Y., Ensunsa J.L., Kimball J., Medici V., Ottaviani J.I. (2022). Absorption, distribution, metabolism and excretion of apigenin and its glycosides in healthy male adults. Free Radic. Biol. Med..

[134] Teng H., Zheng Y., Cao H., Huang Q., Xiao J., Chen L. (2023). Enhancement of bioavailability and bioactivity of diet-derived flavonoids by application of nanotechnology: a review. Crit. Rev. Food Sci. Nutr..

[135] Huang C., Xue L.F., Hu B., Liu H.H., Huang S.B., Khan S. (2021). Calycosin-loaded nanoliposomes as potential nanoplatforms for treatment of diabetic nephropathy through regulation of mitochondrial respiratory function. J. Nanobiotechnology.

[136] Zhao Y., Ding Q., He Q., Zu T., Rong Z., Wu Y. (2025). Reno protective potential of taxifolin liposomes modified by chitosan in diabetic mice. Int. J. Biol. Macromol..

[137] Tong F., Liu S., Yan B., Li X., Ruan S., Yang S. (2017). Quercetin nanoparticle complex attenuated diabetic nephropathy via regulating the expression level of ICAM-1 on endothelium. Int. J. Nanomedicine..

[138] Zheng X.P., Nie Q., Feng J., Fan X.Y., Jin Y.L., Chen G. (2020). Kidney-targeted baicalin-lysozyme conjugate ameliorates renal fibrosis in rats with diabetic nephropathy induced by streptozotocin. BMC Nephrol.

[139] Riva A., Ronchi M., Petrangolini G., Bosisio S., Allegrini P. (2019). Improved oral absorption of Quercetin from Quercetin Phytosome®, a new delivery system based on food grade lecithin. Eur. J. Drug Metab. Pharmacokinet..

[140] Zhang Z.Q., He L.P., Liu Y.H., Liu J., Su Y.X., Chen Y.M. (2014). Association between dietary intake of flavonoid and bone mineral density in middle aged and elderly Chinese women and men. Osteoporos. Int..

[141] Li D., Zhang Y., Liu Y., Sun R., Xia M. (2015). Purified anthocyanin supplementation reduces dyslipidemia, enhances antioxidant capacity, and prevents insulin resistance in diabetic patients. J. Nutr..

[142] Edwards R.L., Lyon T., Litwin S.E., Rabovsky A., Symons J.D., Jalili T. (2007). Quercetin reduces blood pressure in hypertensive subjects. J. Nutr..

[143] Reagan-Shaw S., Nihal M., Ahmad N. (2008). Dose translation from animal to human studies revisited. FASEB J.

[144] Williamson G. (2025). Bioavailability of food polyphenols: current state of knowledge. Annu. Rev. Food Sci. Technol..

[145] Williamson G., Kay C.D., Crozier A. (2018). The bioavailability, transport, and bioactivity of dietary flavonoids: a review from a historical perspective. Compr. Rev. Food Sci. Food Saf..

[146] Carregosa D., Pinto C., Ávila-Gálvez M.Á., Bastos P., Berry D., dos Santos C.N. (2022). A look beyond dietary (poly)phenols: the low molecular weight phenolic metabolites and their concentrations in human circulation. Compr. Rev. Food Sci. Food Saf..

[147] Cassidy A., Minihane A.M. (2017). The role of metabolism (and the microbiome) in defining the clinical efficacy of dietary flavonoids. Am. J. Clin. Nutr..

[148] Favari C., Rinaldi de Alvarenga J.F., Sánchez-Martínez L., Tosi N., Mignogna C., Cremonini E. (2024). Factors driving the inter-individual variability in the metabolism and bioavailability of (poly)phenolic metabolites: a systematic review of human studies. Redox Biol.

[149] Baky M.H., Elshahed M., Wessjohann L., Farag M.A. (2022). Interactions between dietary flavonoids and the gut microbiome: a comprehensive review. Br. J. Nutr..

[150] Li X., Xie E., Sun S., Shen J., Ding Y., Wang J. (2025). Flavonoids for gastrointestinal tract local and associated systemic effects: a review of clinical trials and future perspectives. J. Adv. Res..

[151] Xiao J. (2017). Dietary flavonoid aglycones and their glycosides: Which show better biological significance?. Crit. Rev. Food Sci. Nutr..

[152] Ozdal T., Sela D.A., Xiao J., Boyacioglu D., Chen F., Capanoglu E. (2016). The reciprocal interactions between polyphenols and gut microbiota and effects on bioaccessibility. Nutrients.

[153] Braune A., Blaut M. (2016). Bacterial species involved in the conversion of dietary flavonoids in the human gut. Gut Microbes.

[154] Courts F.L., Williamson G. (2015). The occurrence, fate and biological activities of C-glycosyl flavonoids in the human diet. Crit. Rev. Food Sci. Nutr..

[155] Bang S.H., Hyun Y.J., Shim J., Hong S.W., Kim D.H. (2015). Metabolism of rutin and poncirin by human intestinal microbiota and cloning of their metabolizing α-L-rhamnosidase from Bifidobacterium dentium. J. Microbiol. Biotechnol..

[156] Ávila M., Hidalgo M., Sánchez-Moreno C., Peláez C., Requena T., de Pascual-Teresa S. (2009). Bioconversion of anthocyanin glycosides by bifidobacteria and Lactobacillus. Food Res. Int..

[157] Braune A., Blaut M. (2011). Deglycosylation of puerarin and other aromatic C-glucosides by a newly isolated human intestinal bacterium. Environ. Microbiol..

[158] Shin N.R., Moon J.S., Shin S.Y., Li L., Lee Y.B., Kim T.J., Han N.S. (2016). Isolation and characterization of human intestinal Enterococcus avium EFEL009 converting rutin to quercetin. Lett. Appl. Microbiol..

[159] Pei R., Liu X., Bolling B. (2020). Flavonoids and gut health. Curr. Opin. Biotechnol..

[160] Thilakarathna S.H., Rupasinghe H.P.V. (2013). Flavonoid bioavailability and attempts for bioavailability enhancement. Nutrients.

[161] Mansour H., Slika H., Nasser S.A., Pintus G., Khachab M., Sahebkar A. (2024). Flavonoids, gut microbiota and cardiovascular disease: dynamics and interplay. Pharmacol. Res..

[162] Li Z., Ren Z., Zhao L., Chen L., Yu Y., Wang D. (2023). Unique roles in health promotion of dietary flavonoids through gut microbiota regulation: current understanding and future perspectives. Food Chem.

[163] Xiong H.H., Lin S.Y., Chen L.L., Ouyang K.H., Wang W.J. (2023). The interaction between flavonoids and intestinal microbes: a review. Foods.

[164] Shen Z., Cui T., Liu Y., Wu S., Han C., Li J. (2023). Astragalus membranaceus and Salvia miltiorrhiza ameliorate diabetic kidney disease via the "gut-kidney axis". Phytomedicine.

[165] Mao Z.H., Gao Z.X., Liu D.W., Liu Z.S., Wu P. (2023). Gut microbiota and its metabolites—molecular mechanisms and management strategies in diabetic kidney disease. Front. Immunol..

[166] Hickson L.J., Langhi Prata L.G.P., Bobart S.A., Evans T.K., Giorgadze N., Hashmi S.K. (2019). Senolytics decrease senescent cells in humans: preliminary report from a clinical trial of Dasatinib plus Quercetin in individuals with diabetic kidney disease. EBioMedicine.

