# Supplementary material for: Plant-Derived Flavonoids Ameliorate Diabetic Nephropathy: Molecular Mechanisms and Therapeutic Potential
Source: Adv Nutr. 2026 Sep 30;17(11):100741. doi: 10.1016/j.advnut.2026.100741 (PMC13695383; doi:10.1016/j.advnut.2026.100741)
Supplement: Supplementary file 1 [file mmc1.docx]

**Plant-Derived Flavonoids Ameliorate Diabetic Nephropathy: Molecular Mechanisms and Therapeutic Potential**

Enhui Tang^1^, Shuyang Huang^1^, Huangmin Zhou^1^, Wenzhen Liao^1, *^, Zheqing Zhang^1, *^

^1^ Department of Nutrition and Food Hygiene, Guangdong Provincial Key Laboratory of Tropical Disease Research, School of Public Health, Southern Medical University, Guangzhou 510515, China.

* Corresponding authors.

Wenzhen Liao, E-mail: [wenzhenliao@163.com](mailto:wenzhenliao@163.com).

Zheqing Zhang, E-mail: [zzqaa501@smu.edu.cn](mailto:zzqaa501@smu.edu.cn).

**Table S1. Flavonoids and diabetic nephropathy**

| **Subclass** | **Compounds** | **Experimental setting** | **Model** | **Treatment methods** | **Molecular mechanisms** | **Reference** |
| --- | --- | --- | --- | --- | --- | --- |
| **Flavonol** | Kaempferol | In vivo and in vitro | STZ-induced diabetic C57BL/6J mice | 10 mg/kg/d (i.g.) for 14 weeks | Downregulated TFR6 expression and inhibited the NF-κB pathway | [1] |
|  |  |  | High glucose-induced NRK-52E cells | 2.5 µM for 1 h |  |  |
|  |  | In vivo | db/db mice | 50, 100 mg/kg/d (i.g) for 12 weeks | Inhibited apoptosis and promoted autophagy through the AMPK/mTOR pathway | [2] |
|  |  | In vivo | Unilateral nephrectomy + STZ induced DN in SD rats | 50, 100, 200 mg/kg/d (i.g.) for 12 weeks | Activated PINK1/Parkin-mediated mitophagy | [3] |
|  |  | In vivo | STZ-induced diabetic Wistar rats | 200 mg/kg/d (i.g.) for 8 weeks | Activated the Nrf2/HO-1 antioxidant axis and suppressed apoptosis | [4] |
|  |  | In vitro | High glucose-induced HK-2 cells | 10 μM for 48 h | Inhibit oxidative stress and apoptosis | [5] |
|  |  | In vitro | High glucose-induced NRK-52E and RPTEC cells | 5, 10, 50 μM for 24 h | Inhibited RhoA/Rho-kinase mediated inflammatory signalling | [6] |
|  |  | In vivo and in vitro | STZ-induced diabetic C57BL/6 mice | 50, 100, 200 mg/kg/d (i.g.) for 21 days | Enhanced GLP-1 and insulin release and inhibited RhoA/ROCK-mediated inflammatory and fibrotic signaling | [7] |
|  |  |  | GLUTag and MIN6 cells under normal- and high-glucose conditions | 1, 5, 10, 50 μM; 15 min–2 h for mechanistic assays |  |  |
|  | Quercetin | In vivo and in vitro | db/db mice | 25, 100 mg/kg/d (i.g.) for 12 weeks | Activated the Nrf2/HO-1 pathway to inhibit ferroptosis | [8] |
|  |  |  | High glucose-induced HK-2 cells | 25 µM for 48 h |  |  |

**Table S1. Flavonoids and diabetic nephropathy (continued)**

| **Subclass** | **Compounds** | **Experimental setting** | **Model** | **Treatment methods** | **Molecular mechanisms** | **Reference** |
| --- | --- | --- | --- | --- | --- | --- |
| **Flavonol** | Quercetin | In vivo and in vitro | STZ-induced diabetic SD rats | 100 mg/kg/d (i.g.) for 12 weeks | Activated the Nrf2 pathway to inhibit ferroptosis | [9] |
|  |  |  | High glucose-induced HK-2 cells | 25 µM for 48 h |  |  |
|  |  | In vitro | High glucose-induced NRK-52E cells | 10, 20, 40 µM for 24 h | Inhibited the USP22–Snail1 signaling axis to block EMT | [10] |
|  |  | In vivo and in vitro | HFD+STZ-induced diabetic C57BL/6J mice | 150 mg/kg/d (i.g.) for 4 weeks | Inhibited renal tubular epithelial cell apoptosis via the PI3K/AKT pathway | [11] |
|  |  |  | High glucose-induced HK-2 cells | 25 µM for 48 h |  |  |
|  |  | In vivo and in vitro | *db/db* mice | 50, 100, 150 mg/kg/d (i.g.) for 8 weeks | Attenuates podocyte apoptosis through targeting the EGFR pathway | [12] |
|  |  |  | High glucose-induced conditionally immortalized mouse podocytes | 10, 20, 40 µM for 24 h |  |  |
|  |  | In vivo | STZ-induced DN in SD rats | 10 mg/kg/d (p.o.) for 4 weeks | Attenuated renal oxidative stress and lipid peroxidation | [13] |
|  |  | In vitro | High glucose-induced rat glomerular mesangial cells | 5, 10, 20 μg/mL for 48 h | Inhibited TGF-β1/Smad signaling to reduce mesangial hypertrophy and ECM accumulation | [14] |
|  |  | In vivo | STZ-induced DN in SD rats | 50 mg/kg/d (i.g.) for 12 weeks | Downregulated TGF-β1 and CTGF to attenuate renal fibrosis | [15] |
|  |  | In vitro | High glucose-induced human mesangial cells | 5, 10, 20, 40 μM for 48 h | Suppressed NF-κB/MCP-1 signaling | [16] |
|  |  | In vivo | STZ-induced DN in C57BL/6J mice | 10 mg/kg/d (i.g.) for 4 weeks | Reduced renal oxidative stress and apoptosis | [17] |
|  |  |  |  |  |  |  |

**Table S1. Flavonoids and diabetic nephropathy (continued)**

| **Subclass** | **Compounds** | **Experimental setting** | **Model** | **Treatment methods** | **Molecular mechanisms** | **Reference** |
| --- | --- | --- | --- | --- | --- | --- |
| **Flavonol** | Quercetin | In vivo and in vitro | High glucose-induced HK-2 and NRK-52E cells | 10, 20, 40 μM for 72 h | Inhibited mTORC1/p70S6K signaling to suppress EMT and renal fibrosis | [18] |
|  |  |  | STZ-induced DN in SD rats | 30, 60, 90 mg/kg/d (i.g.) for 14 weeks |  |  |
|  |  | In vivo | STZ-induced DN in apoE^-/-^ mice | 10 mg/kg/d (p.o.) for 4 weeks | Attenuated metabolic dysfunction and glomerulosclerosis | [19] |
|  |  | In vivo | STZ-induced DN in Wistar rats | 25 mg/kg/d (i.p.) for 30 days | Reduced oxidative stress and TGF-β1 expression | [20] |
|  |  | In vivo | STZ-induced DN in SD rats | 50, 100 mg/kg/d (p.o.) for 12 weeks | Inhibited oxidative stress and TGF-β1/Smad signaling | [21] |
|  |  | In vivo and in vitro | High glucose-induced mouse glomerular mesangial cells | 10, 20, 40 μM for 24 h | Reactivated the Hippo pathway to inhibit YAP/TEAD-mediated mesangial cell proliferation | [22] |
|  |  |  | *db/db* mice | 50, 100, 150 mg/kg/d (i.g.) for 8 weeks |  |  |
|  |  | In vivo | HFD/STZ-induced DN in Wistar rats | 50 mg/kg/d (p.o.) for 5 weeks | Inhibited GSK-3β and attenuated oxidative stress and inflammation | [23] |
|  |  | In vivo and in vitro | High-glucose-induced HK-2 cells | 10, 20, 40 μM for 72 h | Inhibited YY1-mediated IL-6/STAT3 signaling to alleviate tubulointerstitial inflammation and fibrosis | [24] |
|  |  |  | *db/db* mice | 50, 100, 150 mg/kg/d (i.g.) for 8 weeks |  |  |
|  |  | In vivo | STZ-induced diabetic Wistar rats | 50 mg/kg/d (gavage); pre- or post-treatmen | Inhibited TGF-β/apelin signaling | [25] |
|  |  | In vivo and in vitro | High glucose-induced RAW264.7 macrophages | 8, 16, 32 μM; 30-min pretreatment before HG for 24 h | Suppressed M1 macrophage polarization via the NLRC5/NLRP3 pathway | [26] |
|  |  |  | HFD/STZ-induced DN in C57BL/6 mice | 50, 100 mg/kg/d (i.g.) for 12 weeks |  |  |

**Table S1. Flavonoids and diabetic nephropathy (continued)**

| **Subclass** | **Compounds** | **Experimental setting** | **Model** | **Treatment methods** | **Molecular mechanisms** | **Reference** |
| --- | --- | --- | --- | --- | --- | --- |
| **Flavonol** | Quercetin | In vivo and in vitro | AGE-induced podocytes | 10 μM; duration not reported | Targeting FoxO1-STX17-mediated autophagic flux | [27] |
|  |  |  | Unilateral nephrectomy/STZ-induced DN in C57BL/6J mice | 150 mg/kg/d (i.g.) for 12 weeks |  |  |
|  |  | In vivo and in vitro | High glucose-induced HK-2 cells | 50 μM for 48 h | Promoted UCP1-mediated alleviation of lipid accumulation | [28] |
|  |  |  | *db/db* mice | 25, 100 mg/kg/d (i.g.) for 12 weeks |  |  |
|  |  | In vitro | High glucose-induced HMCs | 10, 20, 40 µM for 48 h | Attenuated proliferation, inflammation, and oxidative stress by regulating the miR-485-5p/YAP1 pathway | [29] |
|  |  | In vivo | STZ-induced DN in rats | 10 mg/kg (s.c.) every 5 days for 8 weeks | Inhibited inflammatory responses through ICAM-1 regulation | [30] |
|  |  | In vivo | STZ-induced DN in SD rats | 50 mg/kg/d (i.g.) for 8 weeks | Reduced oxidative stress and inflammatory responses | [31] |
|  |  | In vivo | STZ-induced NRK-52E cells | 265.5 μg/mL for 24 h | Reduced oxidative stress, apoptosis, and inflammatory cytokine expression | [32] |
|  |  | In vivo and in vitro | db/db mice | 25 50 mg/kg (p.o.) for 10 weeks | Upregulated PPARα/ACOX1/CPT1A-mediated FAO and alleviated lipotoxicity and fibrotic injury | [33] |
|  |  |  | High glucose-induced HK-2 cells | 30 μM (formal treatment time NR) |  |  |
|  |  | In vivo and in vitro | db/db mice | 50 mg/kg/d (p.o.) for 20 weeks | Activated autophagy and alleviated podocyte dedifferentiation via the Notch pathway | [34] |
|  |  |  | High glucose-induced mouse podocytes | 50 μM |  |  |
|  |  | In vitro | High glucose-induced NRK-52E cells | 10, 50 μg/mL for 48 h | Attenuated ferroptosis-associated oxidative stress and inflammatory responses | [35] |

**Table S1. Flavonoids and diabetic nephropathy (continued) 3**

| **Subclass** | **Compounds** | **Experimental setting** | **Model** | **Treatment methods** | **Molecular mechanisms** | **Reference** |
| --- | --- | --- | --- | --- | --- | --- |
| Flavonol | Quercetin | In vivo | STZ-induced diabetic SD rats | 25, 50, 100 mg/kg/d (i.g.) for 7 weeks | Suppressed NLRP3 inflammasome activation and renal lipid accumulation | [36] |
|  |  | In vivo | Early-stage DN in db/db mice | 50, 100 mg/kg/d (p.o.) for 10 weeks | Improved renal lipid metabolism and reduced lipid accumulation by downregulating the SCAP/SREBP2/LDLr pathway and HMGCR | [37] |
|  | Fisetin | In vivo | STZ-induced eNOS^-/-^ C57BL/6J mice | 5, 10, 20 mg/kg/d (i.g.) for 8 weeks | Activated the Nrf2/HO-1 pathway to inhibit ferroptosis | [38] |
|  |  | In vivo and in vitro | High-glucose-induced mouse podocytes | 5, 10, 50 μM for 48 h | Activated CDKN1B/P70S6K-mediated autophagy and inhibited the NLRP3 inflammasome | [39] |
|  |  |  | STZ-induced DN in eNOS^-/-^ mice | 5, 10, 20 mg/kg/ every 2 days (i.g.) for 8 weeks |  |  |
|  |  | In vivo and in vitro | Palmitate-induced HK-2 cells | 10, 20, 40 μM for 24 h | **Attenuated insulin resistance and inhibited** RIP3-regulated inflammation | [40] |
|  |  |  | HFD-induced DN in C57BL/6 mice | 20, 40, 80 mg/kg/d (i.g.) for 16 weeks |  |  |
|  |  | In vivo and in vitro | TGF-β-induced HK-2 cells | 10, 20, 40 μM for 1 h | Inhibited PPARγ/CD36–TGF-β1/Smad2/3 signaling to attenuate renal fibrosis | [41] |
|  |  |  | STZ-induced diabetic atherosclerosis in LDLR^-/-^ mice | 30 mg/kg/d in HFD for 12 weeks |  |  |
|  | Morin | In vitro | High-glucose-induced rat GMCs | 25, 50 μM for 30 min | Suppressed NOX4/ROS generation and p38 MAPK/JNK signaling | [42] |
|  | Pentamethyl-  quercetin | In vivo and in vitro | Spontaneous type 2 diabetic Goto–Kakizaki rats | 5, 10, 20 mg/kg/d in chow for 16 weeks | Suppressed the TGF-β/Smads signaling | [43] |
|  |  |  | High-glucose-induced rat mesangial cells | 1, 3, 10 μM for 48 h |  |  |

**Table S1. Flavonoids and diabetic nephropathy (continued)**

| **Subclass** | **Compounds** | **Experimental setting** | **Model** | **Treatment methods** | **Molecular mechanisms** | **Reference** |
| --- | --- | --- | --- | --- | --- | --- |
| Flavonol | Isorhamnetin | In vivo | HFD/STZ-induced type 2 DN in Wistar rats | 50 mg/kg/d (i.g.) for 8 weeks | Restored autophagy through downregulation of autophagy-related miRNAs and upregulation of FYCO1, ULK1, TECPR1 and WIPI2 | [44] |
|  | Ombuin | In vivo | HSHFD-induced DN in SD rats | 10, 20, 40 mg/kg/d (p.o.) for 8 weeks | Modulated PPARγ and Notch1 signaling to suppress NF-κB-associated inflammation and TGF-β1/FN/CTGF-mediated fibrosis | [45] |
|  | Galangin | In vivo | STZ-induced DN in Wistar rats | 15 mg/kg/d (i.g.) for 8 weeks | Suppressed NF-κB p65-mediated inflammation and Bax/caspase-3-associated apoptosis while improving antioxidant defenses | [46] |
|  | Troxerutin | In vivo | STZ-induced diabetic Wistar rats | 150 mg/kg/d (p.o.) for 4 weeks | Downregulated TGF-β/miR-192 signaling and upregulated SIP1 | [47] |
|  | Myricetin | In vivo and in vitro | HFD/STZ-induced diabetic kidney disease mice | 200, 300 mg/kg/d (p.o.) for 11 weeks | Inhibited ROCK1/ERK/p38 signaling | [48] |
|  |  |  | High glucose-induced HMCs | 10 μM for 24 h |  |  |
|  |  | In vivo | STZ/Cd-induced diabetic nephrotoxic Wistar rats | 1 mg/kg/d (i.p.) for 12 weeks | Suppressed SREBP expression and ameliorated dyslipidemia, mesangial proliferation, and renal fibrosis | [49] |
|  |  | In vivo | STZ-induced diabetic Wistar rats | 6 mg/rat/d (i.p., 12 h) for 10 days | Enhanced GPx activity and inhibited XO/XDH-mediated oxidative stress, reducing lipid peroxidation and renal fibrosis | [50] |
|  |  |  |  |  |  |  |

**Table S1. Flavonoids and diabetic nephropathy (continued)**

| **Subclass** | **Compounds** | **Experimental setting** | **Model** | **Treatment methods** | **Molecular mechanisms** | **Reference** |
| --- | --- | --- | --- | --- | --- | --- |
| Flavonol | Myricetin | In vivo and in vitro | HFD/STZ-induced DN in C57BL/6J mice | 200, 300 mg/kg/d (p.o.) for 11 weeks | Inhibited the NOX4/ROS/NF-κB/Snail axis and renal tubular EMT | [51] |
|  |  |  | High glucose-induced HK-2 cells | 40, 60 μM for 24 h |  |  |
|  |  | In vivo and in vitro | db/db mice | 50, 100 mg/kg/d (p.o.) for 12 weeks | Activated PI3K/Akt signaling, promoted M2 macrophage polarization, and alleviated renal tubulointerstitial fibrosis | [52] |
|  |  |  | High glucose-induced RAW 264.7 macrophages | 25 μM for 24 h |  |  |
| **Flavonol glycoside** | Hyperoside | In vivo and in vitro | HFD+STZ-induced diabetic C57BL/6J mice | 30 mg/kg/d (i.g.) for 4 weeks | Targeted the miR-499-5p/APC axis | [53] |
|  |  |  | High glucose-induced mouse podocytes | 2 mM for 24 h |  |  |
|  |  | In vivo | STZ-induced DN in C57BL/6 mice | 10, 30 mg/kg/d (p.o.) for 4 weeks | Restored nephrin and podocin expression to ameliorate podocyte slit diaphragm injury and albuminuria | [54] |
|  |  | In vivo | db/db mice | 40, 80 mg/kg/d (i.p.) for 12 weeks | Promoted M1-to-M2 macrophage polarization and Th2/Treg differentiation to suppress renal inflammation | [55] |
|  |  | In vitro | High glucose-induced SV40-MES13 mouse mesangial cells | 50, 100, 200 μM for 48 h | Inhibited the ERK/CREB/miR-34a pathway | [56] |
|  |  | In vivo and in vitro | STZ-induced DN in C57BL/6 mice | 10, 30 mg/kg/d (p.o.) for 4 weeks | Suppressed podocyte heparanase expression and restored glomerular basement membrane heparan sulfate | [57] |
|  |  |  | High-glucose-induced mouse podocytes | 30, 100 μg/mL for 1 h |  |  |
|  |  |  |  |  |  |  |

**Table S1. Flavonoids and diabetic nephropathy (continued)**

| **Subclass** | **Compounds** | **Experimental setting** | **Model** | **Treatment methods** | **Molecular mechanisms** | **Reference** |
| --- | --- | --- | --- | --- | --- | --- |
| **Flavonol glycoside** | Hyperoside | In vivo and in vitro | db/db mice | **50 mg/kg/d (i.p.) for 12 weeks** | Suppressed ROS/ERK1/2-mediated GSDMD pyroptosis | [58] |
|  |  |  | BSA-induced HK-2 cells | 10 μM for 1 h |  |  |
|  | Kaempferitrin | In vitro | AGEs-induced rat glomerular mesangial cells (GMCs) | 10, 20, or 35 μM for 24 h | Suppressed oxidative stress and apoptosis | [59] |
|  | Icariin | In vitro | High glucose-induced conditionally immortalized mouse podocytes | 10 µM for 48 h | Activated GPER to inhibit apoptosis | [60] |
|  |  | In vivo and in vitro | HFHSD+STZ-induced diabetic SD rats | 20, 40, 80 mg/kg/d (i.g.) for 12 weeks | Restored autophagy via regulation of the miR-192-5p/GLP-1R Pathway | [61] |
|  |  |  | High glucose-induced HK-2 and NRK-49F cells | 2, 10, 50 µM for 48 h |  |  |
|  |  | In vivo | STZ-induced DN in SD rats | 80 mg/kg/d (i.g.) for 8 weeks | Downregulated TGF-β1 and type IV collagen expression and attenuated oxidative stress | [62] |
|  |  | In vitro | High-glucose-induced human and rat glomerular mesangial cells | 10⁻⁸–10⁻⁴ M for 48 h | Inhibited GPER-dependent TGF-β/Smad and ERK signaling to reduce ECM accumulation | [63] |
|  |  | In vivo | STZ-induced DN in SD rats | 5 mg/kg/d (i.g.) for 8 weeks | Inhibited TGF-β/Smad/CTGF signaling and oxidative stress and promoted endogenous renal progenitor cell recruitment | [64] |
|  |  | In vivo and in vitro | High-glucose-induced HGMCs | 1, 3, 10 μM for 48 h | Activated Nrf2 through GPER/p62-dependent Keap1 degradation to inhibit oxidative stress | [65] |
|  |  |  | STZ-induced DN in SD rats | 20, 40, 80 mg/kg/d (i.g.) for 9 weeks |  |  |
|  |  |  |  |  |  |  |

**Table S1. Flavonoids and diabetic nephropathy (continued)**

| **Subclass** | **Compounds** | **Experimental setting** | **Model** | **Treatment methods** | **Molecular mechanisms** | **Reference** |
| --- | --- | --- | --- | --- | --- | --- |
| **Flavonol glycoside** | Icariin | In vivo | STZ-induced DN in ICR mice | 150 mg/kg/d (i.g.) for 6 weeks | Inhibited the TLR4/NF-κB pathway | [66] |
|  |  | In vivo and in vitro | High-glucose-induced MPC-5 podocytes | 1, 3, 10 μM for 48 h | Enhanced Sesn2-mediated mitophagy and activated the Keap1-Nrf2/HO-1 axis to inhibit NLRP3 inflammasome activation | [67] |
|  |  |  | STZ-induced DN in SD rats | 20, 40, 80 mg/kg/d (i.g.) for 8 weeks |  |  |
|  |  | In vivo and in vitro | High-glucose-induced NRK-52E cells | 20, 40, 80; unit reported as mg/kg in the article | Regulated the miR-122-5p/FOXP2 axis to inhibit renal tubular EMT and fibrosis | [68] |
|  |  |  | STZ-induced DN in SD rats | 20, 40, 80 mg/kg/d (i.g.) for 5 weeks |  |  |
|  |  | In vitro | High glucose-induced primary rat kidney cells | 10, 25, 50 μM for 24 h | Regulated the miR-503/SIRT4 axis to inhibit ER stress and apoptosis | [69] |
|  |  | In vivo and in vitro | HFD/STZ-induced diabetic SD rats | 20, 40, 80 mg/kg/d (i.g.) for 12 weeks | Activated the AR/RKIP pathway and inhibited MEK/ERK signaling to suppress EndMT and renal fibrosis | [70] |
|  |  |  | *db/db* mice | 30, 60, 120 mg/kg/d (i.g.) for 12 weeks |  |  |
|  |  |  | High glucose-induced mouse glomerular endothelial cells | 3, 10, 30 μM for 48 h |  |  |
|  |  | In vivo and in vitro | High-glucose-induced HK-2 cells | 2 μM for 48 h | Regulated the miR-320a-3p/BMP6/Smad axis to attenuate tubulointerstitial fibrosis | [71] |
|  |  |  | STZ-induced DN in SD rats | 80 mg/kg (i.g.) for 12 weeks |  |  |
|  | Rutin | In vivo | STZ-induced diabetic Wistar rats | 100 mg/kg/d (i.g.) for 45 days | Reduce ECM | [72] |
|  |  | In vivo | STZ-induced DN in SD rats | 10, 30, 90 mg/kg/d (i.g.) for 10 weeks | Attenuated oxidative stress and inhibited the TGF-β1/Smad pathway | [73] |
|  |  | In vivo | Alloxan-induced DN in Wistar rats | 50, 100 mg/kg/d (p.o.) for 6 weeks | Reduced oxidative and ER stress, suppressed ACE/TGF-β1 signaling | [74] |

**Table S1. Flavonoids and diabetic nephropathy (continued)**

| **Subclass** | **Compounds** | **Experimental setting** | **Model** | **Treatment methods** | **Molecular mechanisms** | **Reference** |
| --- | --- | --- | --- | --- | --- | --- |
| **Flavonol glycoside** | Rutin | In vitro | High-glucose-induced human renal glomerular endothelial cells | 12.5, 25, 50 μM for 24 h | Inhibited ROS/RhoA/ROCK signaling to preserve glomerular endothelial barrier function | [75] |
|  |  | In vivo | Alloxan-induced DN in Wistar rats | 100 mg/kg/d (i.g.) for 4 weeks | Downregulated AQP2/AQP3/V2R and TGF-β1/fibronectin expression | [76] |
|  |  | In vivo | STZ-induced DN in SD rats | 100 mg/kg/d (i.g.) for 8 weeks | Upregulated the Nrf2/HO-1 pathway and downregulated Jak-2/Stat3 signaling | [77] |
|  |  | In vivo and in vitro | *db/db* mice | 30, 100 mg/kg/d (i.g.) for 8 weeks | Inhibited Src/EGFR/ERK signaling to attenuate oxidative stress and podocyte injury | [78] |
|  |  |  | High glucose-induced human podocytes | 200, 400 μM for 48 h |  |  |
|  |  | In vivo | STZ-induced diabetic C57BL/6 mice | 50, 100 mg/kg/d (i.g.) for 8 weeks | Attenuated oxidative stress and downregulated TGF-β1 and collagen I expression | [79] |
|  |  | In vivo | db/db mice | 100, 200 mg/kg/d (i.g.) for 8 weeks | Activated autophagy by inhibiting the PI3K/AKT/mTOR pathway | [80] |
|  | **Astragalin** | In vivo and in vitro | STZ-induced diabetic C57BL/6J mice | 5, 10 mg/kg/d (i.g.) for 4 weeks | Activated AMPK/PGC1α signaling to restore mitochondrial quality control | [81] |
|  |  |  | High glucose/palmitic acid-induced HK-2 cells | 20 μM; 12 h for qPCR and 24 h for protein/functional assays |  |  |
|  |  | In vivo and in vitro | STZ-induced diabetic C57BL/6J mice | 10, 20 mg/kg/d (i.g.) for 8 weeks | Promoted ubiquitin-dependent Drp1 degradation | [82] |
|  |  |  | High glucose/palmitic acid-induced HK-2 cells | 5, 20 μM for 24 h |  |  |

**Table S1. Flavonoids and diabetic nephropathy (continued)**

| **Subclass** | **Compounds** | **Experimental setting** | **Model** | **Treatment methods** | **Molecular mechanisms** | **Reference** |
| --- | --- | --- | --- | --- | --- | --- |
| **Flavonol glycoside** | Isoquercitrin | In vivo and in vitro | db/db mice | 40, 80, 120 mg/kg/d (i.g.) for 12 weeks | Inhibited STAT3 phosphorylation and dimerization | [83] |
|  |  |  | High glucose- or IL-6-induced HUVECs and IL-6-induced HK-2 cells | 5, 10, 20 μM |  |  |
|  |  | In vivo and in vitro | HFD/STZ-induced DN in C57BL/6J mice | 10, 20, 40 mg/kg/d (i.p.) for 8 weeks | Downregulated renal SGLT2 expression to improve glucose metabolism | [84] |
|  |  |  | High glucose-induced HK-2 cells | 30, 50, 80 μM for 48 h |  |  |
|  | Reynoutrin | In vivo and in vitro | HFD/STZ-induced DN in C57BL/6 mice | 12.5, 25, 50 mg/kg (i.p.) for 10 weeks | Directly inhibited Fyn and activated downstream Sirt1/Foxo3a and Nrf2 antioxidant signaling | [85] |
|  |  |  | High-glucose-induced primary rat glomerular mesangial cells | Varying concentrations for 36 h; 25 μM used in key target-validation experiments |  |  |
| **Flavanol** | (+)-Catechin | In vivo | HFD+STZ-induced diabetic C57BL/6J mice | 50, 100 mg/kg/d (i.g.) for 8 weeks | inhibited ER stress-related NLRP3 inflammasome activation | [86] |
|  |  | In vitro | High glucose-induced HK-2 cells | 5, 10, 20 µM for 48 h | Inhibited EMT | [87] |
|  |  | In vivo | db/db mice | 15, 30, 60 mg/kg/d (p.o.) for 16 weeks | Trapped methylglyoxal and inhibited AGE/RAGE–NF-κB-mediated inflammation | [88] |
|  | Catechin | In vivo | STZ-induced diabetic Wistar albino rats | 20 mg/kg/d (p.o.) for 30 days | Reduced oxidative stress and TGF-β1 expression | [89] |
|  | Grape seed procyanidin B2 | In vitro | High glucose-induced mouse podocytes (MPC5) | 2.5, 5 or 10 μg/mL for 48 h | Activated AMPK/SIRT1/PGC-1α signaling and reduced mitochondrial dysfunction and apoptosis | [90] |
|  |  |  |  |  |  |  |
|  |  |  |  |  |  |  |

**Table S1. Flavonoids and diabetic nephropathy (continued)**

| **Subclass** | **Compounds** | **Experimental setting** | **Model** | **Treatment methods** | **Molecular mechanisms** | **Reference** |
| --- | --- | --- | --- | --- | --- | --- |
| **Flavanol** | Grape seed procyanidin B2 | In vivo | db/db mice | 30 mg/kg/d (p.o.) for 10 weeks | Restored mimecan expression and inhibited NF-κB-mediated oxidative stress and renal fibrosis | [91] |
|  |  | In vivo | db/db mice | 30 mg/kg/d (p.o.) for 10 weeks | Suppressed MFG-E8/ERK1/2/Akt/GSK-3β signaling and alleviated renal injury | [92] |
|  | Proanthocyanidin | In vivo | STZ-induced diabetic rats | 250 mg/kg/d (i.g.) for 12 weeks | Reduced oxidative stress and inflammation | [93] |
|  | Procyanidin B2 | In vitro | High glucose-induced HK-2 cells | 10 μM for 48 h | Inhibited TGF-β/Smad and MAPK/p38 signaling and suppressed EMT | [94] |
|  | Persimmon peel proanthocyanidins | In vivo | STZ-induced diabetic Wistar rats | 10 mg/kg/d (p.o.) for 20 days | Suppressed oxidative stress and NF-κB-mediated inflammation | [95] |
|  | Grape seed proanthocyanidins | In vivo | HFD/STZ-induced T2DM SD rats | 125, 250 mg/kg/d (i.g.) for 8 weeks | Activated Nrf2/ARE signaling and enhanced antioxidant defenses | [96] |
|  |  | In vivo | HFD/STZ-induced diabetic SD rats | 250 mg/kg/d (i.g.) for 16 weeks | Attenuated ER stress-induced apoptosis via the GRP78/p-ERK/caspase-12 pathway | [97] |
|  |  | In vivo | STZ-induced diabetic Wistar rats | 250 mg/kg/d (p.o.) for 24 weeks | Restored oxidative stress-related protein expression and reduced glycation damage | [98] |
|  |  | In vivo | STZ-induced diabetic Wistar rats | 500 mg/kg/d (p.o.) for 24 weeks | Suppressed AGE/RAGE signaling and CTGF expression, thereby reducing extracellular matrix accumulation | [99] |
|  |  | In vivo | HFSD/CdCl₂-induced DN in Kunming mice | 5 mg/kg/d (i.p.) for 4 weeks | Inhibited p38 MAPK and activated Keap1/Nrf2 signaling, thereby reducing oxidative stress | [100] |
|  |  |  |  |  |  |  |

**Table S1. Flavonoids and diabetic nephropathy (continued)**

| **Subclass** | **Compounds** | **Experimental setting** | **Model** | **Treatment methods** | **Molecular mechanisms** | **Reference** |
| --- | --- | --- | --- | --- | --- | --- |
| **Flavanol** | EGCG | In vitro | AGE-induced HEK293 cells and HMCs | 10 μM for 30 min | Downregulated RAGE and inhibited NF-κB activation to attenuate inflammation and apoptosis | [101] |
|  |  | In vivo | Subtotal nephrectomy + STZ-induced DN in Wistar rats | 25, 50, 100 mg/kg/d (i.g.) for 50 days | Reduced AGE/RAGE-associated NF-κB activation and TGF-β1/fibronectin expression | [102] |
|  |  | In vivo and in vitro | db/db mice | 50, 100 mg/kg/d (i.g.) for 4 or 8 weeks | Suppressed Ang II/AT1R–NADPH oxidase–ERK/p38 MAPK signaling to reduce oxidative stress and renal fibrosis | [103] |
|  |  |  | Ang II induced-HK-2 cells | 15, 30 μM for 6 h |  |  |
|  |  | In vivo and in vitro | STZ-induced DN in C57BL/6 WT and Nrf2^-/-^ mice | 100 mg/kg/d (s.c.) for 24 weeks | Disabled KEAP1 to activate NRF2 and suppress oxidative stress, inflammation, and renal fibrosis | [104] |
|  |  |  | High-glucose-induced mouse mesangial cells | 25, 50, 75, 100 μM for 48 h |  |  |
|  |  | In vivo and in vitro | High-glucose-induced HEK293 cells | 20 μg/mL for 24 h | Targeted Notch1 and inhibited TGF-β/Smad3 signaling to attenuate fibrosis | [105] |
|  |  |  | STZ-induced DN in ICR mice | 100 mg/kg/d (i.p.) for 1 week |  |  |
|  |  | In vivo | HFD/STZ-induced DN in SD rats | 40, 80 mg/kg/d (i.g.) for 8 weeks | Suppressed ER stress-mediated IRE1/XBP1–NLRP3 inflammasome activation and renal inflammation | [106] |
|  |  | In vitro | High glucose-induced human embryonic kidney cells (HEK293) | 2.2 mM | Reduced RAGE expression and inhibited oxidative stress and inflammation | [107] |
|  |  | In vitro | High glucose-induced mouse podocytes | 20 μM for 72 h | Suppressed ER stress and reduced podocyte apoptosis | [108] |

**Table S1. Flavonoids and diabetic nephropathy (continued)**

| **Subclass** | **Compounds** | **Experimental setting** | **Model** | **Treatment methods** | **Molecular mechanisms** | **Reference** |
| --- | --- | --- | --- | --- | --- | --- |
| **Flavanol** | EGCG | In vivo | STZ-induced diabetic ICR mice | 50, 100, 200 mg/kg/d (s.c.) for 1 week | Suppressed osteopontin expression and alleviated proteinuria and renal injury | [109] |
|  |  | In vivo and in vitro | HFD+STZ-induced DN in Wistar rats | 100 mg/kg/d (i.g.) for 30 d | Activated the Keap-1/Nrf2/ARE pathway, inhibited NOX2 activity, and thereby alleviated oxidative stress | [110] |
|  |  |  | PA-induced HK-2 cells | 50, 75, 100 µM for 24 h |  |  |
|  |  | In vivo | HFD+STZ-induced DN in Wistar rats | 100 mg/kg/d (i.g.) for 4 weeks | Inhibited apoptosis | [111] |
|  | (−)-epicatechin | In vitro | High-glucose-induced NRK-52E cells | 10 μM for 2 h | Modulated NOX-4/SIRT-1 signaling and inhibited MAPK activation to attenuate oxidative stress | [112] |
|  |  | In vitro | High glucose + LPS‐induced NRK-52E cells | 1, 5,10 µM for 24 h | Inhibited NOX4/MAPK-mediated inflammation | [113] |
| **Flavone** | Apigenin | In vivo | STZ-induced diabetic Wistar rats | 5, 10, 20 mg/kg/d (i.g.) for 8 months | Reduced oxidative stress, alleviated renal fibrosis, and inhibited MAPK-mediated inflammation | [114] |
|  |  | In vitro | High-glucose-induced HK-2 cells | 100, 200 μM for 48 h | Activated PI3K-dependent Nrf2/HO-1 signaling to inhibit oxidative stress and inflammation | [115] |
|  |  | In vivo and in vitro | STZ-induced DN in Wistar rats | 20 mg/kg/d (p.o.) for 7 days | Regulated the miR-423-5p/USF2 axis | [116] |
|  |  |  | High-glucose-induced NRK-52E cells | Apigenin concentration not explicitly reported |  |  |
|  |  | In vivo | STZ-induced DN in Wistar rats | 40 mg/kg/d (i.p.) for 4 weeks | Downregulated GATA3 and PDGFR-β/NF-κB signaling | [117] |
|  |  |  |  |  |  |  |

**Table S1. Flavonoids and diabetic nephropathy (continued)**

| **Subclass** | **Compounds** | **Experimental setting** | **Model** | **Treatment methods** | **Molecular mechanisms** | **Reference** |
| --- | --- | --- | --- | --- | --- | --- |
| **Flavone** | Apigenin | In vivo and in vitro | Zucker diabetic fatty rats (ZDFRs) | 20 mg/kg (i.g.), 5 days/week for 4 weeks | Inhibited CD38 and restored the NAD⁺/NADH ratio and Sirt3 activity to attenuate mitochondrial oxidative stress | [118] |
|  |  |  | High-glucose-induced HK-2 cells | 10 μM for 24 h |  |  |
|  | Chrysin | In vivo | db/db mice | 10 mg/kg/d (i.g.) for 10 weeks | Inhibited the PERK-eIF2α-ATF-CHOP pathway | [119] |
|  |  | In vivo | HFD/STZ-induced DN in Wistar rats | 40 mg/kg/d (i.g.) for 16 weeks | Inhibited TNF-α/NF-κB signaling and reduced TGF-β-mediated ECM accumulation | [120] |
|  |  | In vivo and in vitro | High-glucose-induced human renal proximal tubular epithelial cells (RPTEC) | 1, 10, 20 μM for 72 h | Inhibited EMT | [121] |
|  |  |  | db/db mice | 10 mg/kg/d (p.o.) for 10 weeks |  |  |
|  |  | In vivo and in vitro | High glucose- and AGE-induced HRMCs | 1, 10, 20 μM for 72 h | Inhibited AGE/RAGE-associated TGF-β/Smad signaling | [122] |
|  |  |  | db/db mice | 10 mg/kg/d (i.g.) for 10 weeks |  |  |
|  |  | In vitro | High glucose- and AGE-induced HRMCs | 1, 10, 20 μM for 72 h | Suppressed autophagy/mTOR-associated actin cytoskeleton and focal adhesion remodeling to inhibit mesangial cell migration | [123] |
| **Flavone** | Tangeretin | In vivo and in vitro | *db*/*db* mice | 10 mg/kg/d (i.g.) for 10 weeks | Inhibited EMT | [124] |
|  |  |  | High glucose‐induced conditionally immortalized mouse podocytes | 1, 10, 20 µM for 4 d |  |  |
|  |  |  |  |  |  |  |

**Table S1. Flavonoids and diabetic nephropathy (continued)**

| **Subclass** | **Compounds** | **Experimental setting** | **Model** | **Treatment methods** | **Molecular mechanisms** | **Reference** |
| --- | --- | --- | --- | --- | --- | --- |
| **Flavone** | Tangeretin | In vitro | High glucose-induced HGMCs | 2.5, 5, 10 μM for 1h | Inhibited ECM accumulation | [125] |
|  |  | In vitro | High glucose-induced HMCs | 5, 10, 20, 30 μM for 24 h | Inhibited the STAT3/NF-κB pathway to suppress inflammation and ECM accumulation | [126] |
|  |  | In vivo | STZ-induced DN in Wistar rats | 25 mg/kg (p.o.) for 8 weeks | Attenuated oxidative stress, inflammation and dyslipidemia | [127] |
|  |  | In vivo | STZ/Cd-induced DN in Wistar rats | 10, 20, 40 mg/kg/d (i.g.) for 12 weeks | Inhibited NF-κB signaling and regulated Bax/Bcl-2 expression | [128] |
|  | Wogonin | In vivo | *db*/*db* mice | 40 mg/kg/every other day (i.g.) for 12 weeks | Upregulated SOCS3 to alleviate renal injury by inhibiting the TLR4-mediated JAK/STAT/  AIM2 pathway | [129] |
|  |  | In vivo | STZ-induced diabetic C57BL/6J mice | 10, 20, 40 mg/kg/every other day (i.g.) for 16 weeks | Activated autophagy by inhibiting the PI3K/AKT/mTOR pathway | [130] |
|  |  | In vivo and in vitro | STZ-induced diabetic C57BL/6J mice | 10, 20, 40 mg/kg/every other day (i.g.) for 12 weeks | Inhibited NF-κB and TGF-β/Smad signaling pathways to ameliorate renal inflammation and fibrosis | [131] |
|  |  |  | High glucose-induced SV-40 cells | 1.5825, 3.125, 6.25 µg/mL for 24 h |  |  |
|  |  | In vivo | STZ-induced diabetic C57BL/6J mice | 10, 20, 40 mg/kg/every other day (i.g.) for 12 weeks | Targeted Bcl-2-mediated autophagy and apoptosis | [132] |
|  |  | In vivo | HSHFD/STZ-induced DN in Wistar rats | 25, 50 mg/kg/d (p.o.) for 2 months | Attenuated oxidative stress, inflammation and dyslipidemia to improve renal dysfunction | [133] |
|  |  |  |  |  |  |  |

**Table S1. Flavonoids and diabetic nephropathy (continued)**

| **Subclass** | **Compounds** | **Experimental setting** | **Model** | **Treatment methods** | **Molecular mechanisms** | **Reference** |
| --- | --- | --- | --- | --- | --- | --- |
| **Flavone** | Baicalein | In vivo | HFD/STZ-induced DN in Wistar rats | 10, 20 mg/kg/d (i.g.) for 16 weeks | Inhibited NF-κB activation and downregulated iNOS and TGF-β1 to attenuate inflammation and renal injury | [134] |
|  | Luteolin | In vivo | STZ-induced diabetic ICR mice | 10, 20 mg/kg/d (i.g.) for 4 weeks | Inhibited RIP140/NF-κB signaling and restored IRS-1/PI3K/Akt insulin signaling | [135] |
|  |  | In vivo | STZ-induced DN in SD rats | 200 mg/kg (i.g.) for 8 weeks | Upregulated HO-1 and Akt phosphorylation | [136] |
|  |  | In vivo | db/db mice | 10 mg/kg/d (p.o.) | Inhibited STAT3 activation | [137] |
|  |  | In vitro | High glucose-induced MPC-5 podocytes | 25, 50, 100 μM for 24 h | Suppressed ROS-dependent NLRP3 inflammasome activation | [138] |
|  |  | In vivo | STZ-induced hyperglycemic renal injury in Wistar rats | 100 mg/kg (i.p.) for 2 weeks | Activated Nrf2-mediated antioxidant defense and suppressed NF-κB inflammation and PI3K/Akt-associated apoptosis/fibrosis | [139] |
|  |  | In vivo and in vitro | STZ-induced DN in Nrf2^+/+^ and Nrf2^-/-^ C57BL/6 mice | 25, 50 mg/kg/d (i.g.) for 10 weeks | Activated Nrf2-dependent antioxidant and anti-inflammatory responses to suppress NF-κB activation and renal fibrosis | [140] |
|  |  |  | High glucose-induced SV40-Mes13 mouse mesangial cells | 62.5, 125, 250 nM for 24 h |  |  |
|  | Acacetin | In vivo | HFD/STZ-induced DN in C57BL/6 mice | 15 mg/kg for 4 weeks | Upregulated AMPK/SIRT1 signaling and suppressed NF-κB/ASC/NLRP3 inflammasome-associated inflammation/pyroptosis and renal fibrosis | [141] |

**Table S1. Flavonoids and diabetic nephropathy (continued)**

| **Subclass** | **Compounds** | **Experimental setting** | **Model** | **Treatment methods** | **Molecular mechanisms** | **Reference** |
| --- | --- | --- | --- | --- | --- | --- |
| **Flavone** | Acacetin | In vivo | STZ-induced diabetic SD rats | 10 mg/kg/d (p.o.) for 8 weeks | Inhibited TLR4/NF-κB pathway and reduced inflammation and oxidative stress | [142] |
|  | Morusin | In vivo and in vitro | db/db mice | 5, 10, 25 mg/kg/d (i.p.) for 12 weeks | Activated SIRT1-mediated deacetylation and inactivation of HIF-1α and suppressed IL-16-associated inflammation | [143] |
|  |  |  | STZ-induced DN in C57BL/6J mice | 10 mg/kg/d (i.p.) for 12 weeks |  |  |
|  |  |  | Hypoxia-induced HK-2 cells | 10 μM for 24 h under 1% O₂ |  |  |
|  | Hispidulin | In vitro | High-glucose-induced MPC-5 cells | 2, 5 μM for 24 h | Regulated the Pim1/p21/mTOR axis | [144] |
|  | Breviscapine | In vivo | STZ-induced diabetic Munich-Wistar rats | 20 mg/kg/d (i.g.) for 8 weeks | Reduced oxidative stress and inhibited PKC/TGF-β1 signaling | [145] |
|  | 5,7,3′,4′,5′-Pentamethoxyflavone | In vivo and in vitro | STZ-induced DN in SD rats | 5, 25 mg/kg (i.g.) for 3 months | Inhibited Smad2/3 phosphorylation and fibronectin accumulation | [146] |
|  |  |  | High-glucose-induced mesangial cells | 5, 10 μM for 6 h |  |  |
| **Flavone glycoside** | Baicalin | In vitro | High glucose + TNF-α + AGEs‐induced HK-2 cells | 5, 10 µM for 72 h or 96h | Inhibited the NF-κB and JAK/STAT pathways | [147] |
|  |  | In vivo | db/db mice | 400 mg/kg/d (i.g.) for 8 weeks | Activated the Nrf2 pathway to reduce oxidative stress and inhibited the MAPK-mediated inflammation | [148] |
|  |  | In vitro | High glucose/AGE/TNF-α-induced HK-2 cells | 5, 10 μM for 72 or 96 h, depending on the assay | Regulated GABAR-mediated Ca²⁺ signaling to inhibit NF-κB and JAK2/STAT3 activation and renal fibrosis | [149] |
|  |  |  |  |  |  |  |

**Table S1. Flavonoids and diabetic nephropathy (continued)**

| **Subclass** | **Compounds** | **Experimental setting** | **Model** | **Treatment methods** | **Molecular mechanisms** | **Reference** |
| --- | --- | --- | --- | --- | --- | --- |
| **Flavone glycoside** | Baicalin | In vivo | HSHFD/STZ-induced DN in C57BL/6J mice | 80 mg/kg/d (i.g.) for 3 months | Inhibited TLR4/MyD88/NF-κB signaling to suppress renal inflammation | [150] |
|  |  | In vivo and in vitro | STZ-induced DN in C57BL/6 mice | 15, 30, 45 mg/kg/d (p.o.) for 8 weeks | Upregulated miR-124 to inhibit TLR4/NF-κB signaling and renal fibrosis | [151] |
|  |  |  | High-glucose-induced HK-2 cells | 100 μM; treatment duration not explicitly reported |  |  |
|  |  | In vivo and in vitro | db/db mice | 50 mg/kg/d (i.p.) for 12 weeks | Upregulated CPT1α-mediated fatty acid oxidation to reduce lipid accumulation and renal fibrosis | [152] |
|  |  |  | High glucose-induced HK-2 cells | 50 μM for 24 h |  |  |
|  |  | In vivo and in vitro | STZ-induced diabetic Kunming mice | 40 mg/kg/d (i.g.) for 7 days | Reversed DNMT3a-associated Klotho promoter hypermethylation and restored Klotho to suppress TGF-β1/Smad-mediated EMT and renal fibrosis | [153] |
|  |  |  | High-glucose-induced HK-2 cells | 6 μM; treatment duration not explicitly reported |  |  |
|  |  | In vivo | STZ-induced DN in SD rats | 160 mg/kg/d (i.g.) for 8 weeks | Inhibited inflammation and renal fibrosis through NF-κB/TGF-β/Smad3 signaling | [154] |
|  |  | In vivo | db/db mice | BA 20 mg/kg/d for 10 weeks | Inhibited TGF-β/Smad signaling and reduced renal fibrosis | [155] |
|  | Diosmin | In vitro | High glucose-induced HK-2 cells | 10 µg/mL for 48 h | Regulated the PTEN PI3K/AKT axis to attenuate ER stress | [156] |
|  |  | In vivo | Alloxan-induced DN in Wistar rats | 50, 100 mg/kg/d (p.o.) for 4 weeks | Modulated the NF-kB signaling and attenuated oxidative stress | [157] |

**Table S1. Flavonoids and diabetic nephropathy (continued)**

| **Subclass** | **Compounds** | **Experimental setting** | **Model** | **Treatment methods** | **Molecular mechanisms** | **Reference** |
| --- | --- | --- | --- | --- | --- | --- |
| **Flavone glycoside** | Diosmetin | In vivo | STZ-induced DN in male albino mice | 25, 50, 100 mg/kg/d (p.o.) for 8 weeks | Modulated Akt/NF-κB/iNOS signaling | [158] |
|  | Orientin | In vitro | High glucose-induced MPC-5 mouse podocytes | 40, 80, 120 μM for 48 h | Restored protective autophagy/mitophagy and mitochondrial membrane potential to attenuate podocyte apoptosis | [159] |
|  | Wogonoside | In vivo and in vitro | HFD+STZ-induced diabetic C57BL/6J mice | 12.5, 25, 50 mg/kg/d (i.g.) for 10 weeks | modulated the HNF4A-NRF2 axis to alleviate oxidative stress | [160] |
|  |  |  | High glucose-induced TCMK-1 cells | 10, 20, 40 µM for 24 h |  |  |
|  |  | In vivo and in vitro | HFD/STZ-inducedDN in C57BL/6J mice | 12.5, 25, 50 mg/kg/d (i.g.) for 10 weeks | Directly targeted NF-κB p65 and inhibited the p65-MMP28 axis to attenuate oxidative stress, inflammation and podocyte injury | [161] |
|  |  |  | High glucose-induced MPC-5 podocytes | 10, 20, 40 Μm for 1 h |  |  |
|  | Scutellarin | In vivo | STZ-induced diabetic C57BL/6J mice | 10, 40 mg/kg/d (i.g.) for 4 weeks | Blocked the TGF-β/Smad pathway | [162] |
|  | Vitexin | In vivo and in vitro | HFD/STZ-induced DN in SD rats | 10, 20 mg/kg/d (i.p.) for 4 weeks | Upregulated GPX4-mediated antioxidant defense | [163] |
|  |  |  | High-glucose-induced HK-2 cells | 10, 20 μM for 24 h |  |  |
|  |  | In vivo | HFD-induced DN in C57BL/6 mice | 15, 30, 60 mg/kg (p.o.) for 12 weeks | Modulated NF-κB/IκBα and AMPK/ACC signaling | [164] |
| **Flavanone** | Naringenin | In vitro | High glucose-induced HK-2 cells | 50, 100, 200 µM for 48 h | Activated the SIRT1/FOXO3a axis and Nrf2 pathway to inhibit ferroptosis | [165] |
|  |  | In vivo | STZ-induced DN in BALB/cA mice | 0.5%, 1%, 2% (w/w) in diet for 10 weeks | Suppressed PKC activity and NF-κB activation | [166] |

**Table S1. Flavonoids and diabetic nephropathy (continued)**

| **Subclass** | **Compounds** | **Experimental setting** | **Model** | **Treatment methods** | **Molecular mechanisms** | **Reference** |
| --- | --- | --- | --- | --- | --- | --- |
| **Flavanone** | Naringenin | In vivo and in vitro | HSHFD/STZ-induced DN in SD rats | 50 mg/kg/d (i.g.) for 6 weeks | Upregulated let-7a to suppress TGFBR1/TGF-β1/Smad signaling and ECM accumulation | [167] |
|  |  |  | High-glucose-induced mouse glomerular mesangial cells (MGMCs) | 100, 200, 400, 600, 1000 μM screened; 24–36 h; exact dose used for subsequent mechanistic NAR group not explicitly stated |  |  |
|  |  | In vivo and in vitro | STZ/nicotinamide-induced diabetic Wistar rats | 25, 50, 100 mg/kg/d (i.g.) for 4 weeks | Suppressed ER stress and ATF4/CHOP-mediated apoptosis | [168] |
|  |  |  | High glucose-induced NRK-52E cells | 25 μM for 48 h |  |  |
|  |  | In vivo | STZ-induced diabetic Wistar rats | 5, 10 mg/kg/d (i.g.) for 10 weeks | Attenuated oxidative stress and downregulated TGF-β1 and IL-1 to reduce renal apoptosis and injury | [169] |
|  |  | In vivo | STZ-induced diabetic rats | 40 mg/kg/d (p.o.) for 12 weeks | Reduced oxidative stress and mitochondrial dysfunction | [170] |
|  |  | In vivo and in vitro | HFD/STZ-induced DN in Mus musculus castaneus mice | 25, 75 mg/kg/d (i.g.) for 4 weeks | Activated CYP4A/20-HETE signaling and upregulated PPARα/β/γ to ameliorate renal injury | [171] |
|  |  |  | High-glucose-induced NRK-52E cells | 0.01, 0.1, 1 μM for 48 h |  |  |
|  |  |  |  |  |  |  |
|  |  |  |  |  |  |  |

**Table S1. Flavonoids and diabetic nephropathy (continued)**

| **Subclass** | **Compounds** | **Experimental setting** | **Model** | **Treatment methods** | **Molecular mechanisms** | **Reference** |
| --- | --- | --- | --- | --- | --- | --- |
| **Flavanone** | Naringenin | In vivo and in vitro | STZ-induced DN in SD rats | 20, 40, 80 mg/kg/d (i.v., tail vein) for 12 weeks | Inhibited NOX4-mediated oxidative stress | [172] |
|  |  |  | High glucose-induced mouse podocytes | 10 μM for 2 h |  |  |
|  |  | In vitro | High glucose-induced rat glomerular mesangial cells (HBZY-1) | 40 μM for 48 h | Inhibited the NLRP3/caspase-1/IL-1β/IL-18 pathway to suppress mesangial inflammation and proliferation | [173] |
|  |  | In vivo and in vitro | STZ/nicotinamide-induced diabetic Wistar rats | 25, 50, 100 mg/kg/d (i.g.) for 4 weeks | Suppressed PERK/eIF2α/ATF4/CHOP-mediated ER stress and apoptosis | [174] |
|  |  |  | High-glucose-induced NRK-52E cells | 25 μM for 48 h |  |  |
|  |  | In vivo | STZ-induced DN in Wistar rats | 50 mg/kg/d (p.o.) for 8 weeks | Reduced oxidative stress and inflammation through the Nrf2/NF-κB pathway | [175] |
|  | Hesperetin | In vivo | HFD/STZ-induced DN in ICR mice | 40, 80 mg/kg/d (i.p.) for 4 weeks | Downregulated TGF-β1/ILK/Akt signaling | [176] |
|  |  | In vivo | STZ-induced diabetic SD rats | 50, 150 mg/kg/d (p.o.) for 10 weeks | Activated Nrf2/ARE/Glo-1 signaling and inhibited the AGEs/RAGE axis and inflammation | [177] |
|  |  | In vivo | STZ-induced diabetic Wistar rats | 20 mg/kg (p.o.) every other day for 6 weeks | Reduced renal oxidative stress and enhanced antioxidant defenses | [178] |
|  | Eriodictyol | In vivo | STZ-induced DN in Wistar rats | 20 mg/kg/d (i.g.) for 12 weeks | Activated Nrf2 and suppressed NF-κB-mediated oxidative stress and inflammation | [179] |
|  |  | In vitro | High glucose-induced HMCs | 12.5, 25 μM for 24 h | Inhibited Akt/NF-κB signaling | [180] |

**Table S1. Flavonoids and diabetic nephropathy (continued)**

| **Subclass** | **Compounds** | **Experimental setting** | **Model** | **Treatment methods** | **Molecular mechanisms** | **Reference** |
| --- | --- | --- | --- | --- | --- | --- |
| **Flavanone** | Erigeroflavanone | In vitro | High glucose-induced SV40 MES 13 cells | 10 μM for 25 h | Reduced ROS generation and inhibited MAPK/caspase-3-mediated cell death | [181] |
|  | Pinocembrin | In vivo | STZ-induced diabetic Wistar rats | 10 mg/kg/d (p.o.) for 20 days | Exerted a possible pro-oxidant effect and aggravated established renal injury | [182] |
|  | Liquiritigenin | In vitro | High-glucose-induced HBZY-1 cells | 20, 40 μM for 24 h | Suppressed NF-κB/NLRP3 inflammasome activation and NOX4-associated oxidative stress, reducing inflammation and ECM accumulation | [183] |
| Flavanone glycoside | Flavanomarein | In vitro | High-glucose-induced HK-2 cells | 40, 60, 80 μM for 48 h | Directly targeted Syk and suppressed Syk/TGF-β1/Smad signaling | [184] |
| **Isoflavone** | Calycosin | In vivo and in vitro | db/db mice | 10, 20 mg/kg/d  (i.p.) for 4 weeks | Restored the GSH–GPX4 antioxidant defense, downregulated NCOA4 expression, and attenuated lipid peroxidation | [185] |
|  |  |  | High glucose-induced HK-2 cells | 10, 20, 40 µM for 24 h |  |  |
|  |  | In vivo | STZ-induced diabetic SD rats | 5, 10 mg/kg/d (p.o.) for 10 weeks | Activated Nrf2 and suppressed NF-κB/p65/NLRP3/TXNIP-mediated pyroptotic signaling | [186] |
|  |  | In vivo and in vitro | db/db mice | 10 mg/kg/d (i.p.) for 4 weeks | Inhibited IκBα/NF-κB p65 phosphorylation | [187] |
|  |  |  | AGEs-induced mouse tubular epithelial cells (mTEC) | 10 μM for 2 h |  |  |
|  |  |  |  |  |  |  |

**Table S1. Flavonoids and diabetic nephropathy (continued)**

| **Subclass** | **Compounds** | **Experimental setting** | **Model** | **Treatment methods** | **Molecular mechanisms** | **Reference** |
| --- | --- | --- | --- | --- | --- | --- |
| **Isoflavone** | Calycosin | In vivo | HFD/STZ-induced diabetic SD rats | 5 mg/kg/d (p.o.) for 8 weeks | Inhibited IL-33/ST2/NF-κB signaling and activated Nrf2, reducing inflammation, oxidative stress, and renal fibrosis | [188] |
|  |  | In vivo | STZ-induced DN in C57BL/6 mice | 30 mg/kg/d (p.o.) for 4 weeks | Reduced oxidative stress and improved mitochondrial function | [189] |
|  |  | In vitro | High glucose-induced rat mesangial cells | 10 and 100 μM; duration NR | Inhibited early mesangial cell proliferation | [190] |
|  |  |  | AGEs-induced human glomerular endothelial cells | 1, 10, and 100 μM; duration NR | Attenuated AGEs-induced apoptosis |  |
|  | Formononetin | In vivo | db/db mice | 25, 50 mg/kg/d, (i.g.) for 8 weeks | Activated the Nrf2/ARE pathway via Sirt1 | [191] |
|  |  | In vivo | HFD/STZ-induced diabetic SD rats | 10, 20, 40 mg/kg/d (p.o.) for 16 weeks | Upregulated SIRT1 and attenuated oxidative stress | [192] |
|  |  | In vivo | db/db mice | 30, 60 mg/kg/d (i.g.) for 12 weeks | Activated autophagy by inhibiting the PI3K/AKT/mTOR pathway | [193] |
|  |  | In vivo and in vitro | STZ-induced DN in SD rats | 20 mg/kg/d (i.g.) for 8 weeks | Activated the Sirt1/PGC-1α pathway to improve mitochondrial dynamics and inhibit tubular cell apoptosis | [194] |
|  |  |  | High glucose-induced HK-2 cells | 10, 20 μM for 48 h |  |  |
|  | Genistein | In vivo | Alloxan-induced diabetic ICR mice | 0.025% or 0.1% genistein-supplemented diet for 2 weeks | Enhanced Nrf2-mediated antioxidant defense and suppressed NF-κB-associated inflammation and PKCβII/TGF-β1-associated fibrotic responses | [195] |
|  |  | In vivo | HSHF-induced DN in SD rats | 30, 50 mg/kg/d (i.g.) for 6 weeks | Upregulated Mfn2 and suppressed NOX4/p38 MAPK/NF-κB signaling | [196] |

**Table S1. Flavonoids and diabetic nephropathy (continued)**

| **Subclass** | **Compounds** | **Experimental setting** | **Model** | **Treatment methods** | **Molecular mechanisms** | **Reference** |
| --- | --- | --- | --- | --- | --- | --- |
| **Isoflavone** | Genistein | In vitro | High glucose-induced immortalized mouse podocytes | 20 μM for 6 h under 30 mM glucose | Inhibited mTOR signaling to enhance autophagy | [197] |
|  |  | In vitro | High-glucose-induced rat mesangial cells | 10 μM for 12, 24, 36, 48 h | Suppressed TGF-β expression | [198] |
|  | Genistein + daidzein | In vivo and in vitro | Type 2 diabetic KKAy mice | Genistein 12 mg/kg/d + daidzein 8 mg/kg/d (p.o.) for 3 months | Suppressed ROS/ERK/AP-1 signaling and reduced NOX expression, oxidative stress, and inflammatory responses | [199] |
|  | Genistein |  | High glucose and albumin-induced immortalized mouse renal tubular cells (imTCs) | 30 μM for 24 h |  |  |
|  | Tectorigenin | In vivo and in vitro | db/db mice | 75 mg/kg/d for 12 weeks | Activated AdipoR1/2–LKB1/AMPKα-PPARα signaling | [200] |
|  |  |  | High-glucose-induced human renal glomerular endothelial cells (HRGECs) | 5, 10 μM for 24 h |  |  |
| Isoflavan | Glabridin | In vivo and in vitro | HFD/STZ-induced DN in SD rats | 50 mg/kg (i.p.) for 4 weeks | Suppressed ferroptosis and downregulated VEGF/Akt/ERK signaling | [201] |
|  |  |  | High-glucose-induced NRK-52E cells | Glabridin concentration and treatment duration not explicitly reported |  |  |
| **Isoflavone glycoside** | Puerarin | In vitro | High glucose-induced HBZY1 cells | 1, 10 µM for 24 h | Inhibited ferroptosis | [202] |
|  |  |  |  |  |  |  |

**Table S1. Flavonoids and diabetic nephropathy (continued)**

| **Subclass** | **Compounds** | **Experimental setting** | **Model** | **Treatment methods** | **Molecular mechanisms** | **Reference** |
| --- | --- | --- | --- | --- | --- | --- |
| **Isoflavone glycoside** | Puerarin | In vitro | High-glucose-induced rat glomerular mesangial cells (GMCs) | 0.1, 1, 10 μM for 24, 48, 96 h | Inhibited PKC activation and downregulated c-Fos/c-Jun to reduce type IV collagen accumulation | [203] |
|  |  | In vivo | HSHFD/STZ-induced DN in C57BL/6J mice | 40, 80 mg/kg/d (i.g.) for 8 weeks | Activated AMPK/Nrf2 signaling | [204] |
|  |  | In vivo and in vitro | STZ-induced DN in C57BL/6J mice | 20, 40, 80 mg/kg for 12 weeks | Inhibited Caspase-1/GSDMD-mediated pyroptosis and reduced IL-1β and IL-18 release | [205] |
|  |  |  | High-glucose-induced MPC5 podocytes | 0.25, 0.5, 1 mM for 24 h |  |  |
|  |  | In vivo and in vitro | STZ-induced DN in eNOS^-/-^ C57BL/6 mice | 20 mg/kg/d (i.g.) for 8 weeks | Upregulated SIRT1 to promote NF-κB deacetylation and suppress NOX4-mediated oxidative stress | [206] |
|  |  |  | High-glucose-induced mouse podocytes | 1, 10 μM for 24 h |  |  |
|  |  | In vivo | High-glucose-induced MPC5 podocytes | 0.25, 0.5, 1 mM for 24 h | Downregulated renal eNOS expression | [207] |
|  |  | In vivo and in vitro | db/db mice | 20 mg/kg/d (i.g.) for 6 weeks | Directly targeted Gnai1 to activate cAMP/PKA/CREB signaling | [208] |
|  |  |  | High glucose-induced human podocytes | 10 μM for 24 h |  |  |
|  |  | In vivo | STZ-induced DN in SD rats | 0.25, 0.5, 1.0 mg/kg/d (i.g.) | Downregulated ICAM-1 and TNF-α expression | [209] |
|  |  | In vivo | High glucose-induced SV40-MES-13 mouse mesangial cells | 2.5, 5, 10 μM for 1 h | Inhibited the AGE/RAGE/PKCβ/NOX4 axis to attenuate oxidative stress | [210] |
|  |  | In vivo | STZ-induced DN in C57BL/6 mice | 20, 40, 80 mg/kg/d (i.g.) for 8 weeks | Upregulated SIRT1/FOXO1/PGC-1α and downregulated NF-κB to attenuate oxidative stress and mitochondrial injury | [211] |

**Table S1. Flavonoids and diabetic nephropathy (continued)**

| **Subclass** | **Compounds** | **Experimental setting** | **Model** | **Treatment methods** | **Molecular mechanisms** | **Reference** |
| --- | --- | --- | --- | --- | --- | --- |
| **Isoflavone glycoside** | Puerarin | In vivo and in vitro | db/db mice | 10 mg/kg/d (p.o.) for 6 weeks | Inhibited NF-κB activation through PP2A/Sirt1 pathway | [212] |
|  |  |  | High glucose-induced human podocytes | 10 μM for 24 h |  |  |
|  |  | In vivo and in vitro | STZ-induced DN in C57BL/6 mice | 5, 10, 20, 40 mg/kg/d (i.g.) for 12 weeks | Upregulated HMOX-1/Sirt1 and activated LKB1/AMPK-dependent autophagy to inhibit podocyte apoptosis | [213] |
|  |  |  | High glucose-induced mouse podocytes | 10 Μm for 15 min |  |  |
|  |  | In vivo | STZ-induced DN in SD rats | 80 mg/kg/d by injection for 16 weeks | Reduced collagen I and restored HSPG expression in glomerular extracellular matrix | [214] |
|  |  | In vivo | STZ-induced early diabetic kidney injury in Wistar rats | 100 mg/kg/d (i.p.) for 7 days | Suppressed oxidative stress and protein S-nitrosylation-associated MMP-9 expression to protect podocytes | [215] |
|  |  | In vivo and in vitro | STZ-induced DN in C57BL/6J mice | 10, 20, 40 mg/kg/d, (i.g.) for 12 weeks | Modulated the SIRT1/NLRP3/caspase-1 pathway | [216] |
|  |  |  | High glucose-induced MPC-5 cells | 0.8 mM for 24 h |  |  |
| **Chalcone** | Isoliquiritigenin | In vivo and in vitro | HFD+STZ-induced diabetic C57BL/6J mice | 10, 30 mg/kg/d (i.g.) for 10 weeks | Reduced oxidative stress and inhibited the TLR4/NF-κB/NLRP3 pathway | [217] |
|  |  |  | High glucose-induced NRK-52E cells | 20 µM for 24 h |  |  |
|  |  | In vivo | *db/db* mice | 5, 10, 20 mg/kg/d (i.g.) for 12 weeks | Inhibited the AGE/RAGE axis | [218] |

**Table S1. Flavonoids and diabetic nephropathy (continued)**

| **Subclass** | **Compounds** | **Experimental setting** | **Model** | **Treatment methods** | **Molecular mechanisms** | **Reference** |
| --- | --- | --- | --- | --- | --- | --- |
| **Chalcone** | Isoliquiritigenin | In vitro | High glucose-induced HMCs | 1, 10, 20 µM for 72 h | Blocked the  TGF-β/Smad pathway to reduce mesangial matrix accumulation | [219] |
|  |  | In vivo and in vitro | HFD/STZ-induced DN in SD rats | 10, 20, 40 mg/kg/d for 8 weeks | Inhibited JAK2/STAT3 signaling and attenuated oxidative stress | [220] |
|  |  |  | High glucose-induced HK-2 cells | 20, 40, 80 μg/mL for 24 h |  |  |
|  |  | In vitro | AGE-induced HK-2 cells | 100, 200 nM for 48 h | Suppressed RAGE/TGF-β/STAT3-associated tubular epithelial–myofibroblast transition and collagen accumulation | [221] |
|  |  | In vivo and in vitro | STZ-induced diabetic C57BL/6J mice | 10, 20 mg/kg/every 2 days (i.g.) for 12 weeks | Reduced oxidative stress and inhibited MAPK-mediated inflammation | [222] |
|  |  |  | High glucose-induced NRK-52E cells | 10, 20 µM for 1 h |  |  |
|  | Isobavachalcone | In vivo and in vitro | STZ-induced DN in SD rats | 12.5, 25, 50 mg/kg/d (p.o.) for 10 weeks | Inhibited NF-κB signaling | [223] |
|  |  |  | High-glucose-induced human renal glomerular endothelial cells (HRGECs) | 10 μM for 48 h |  |  |
|  | Dibenzoylmethane | In vivo and in vitro | High-fat diet-induced diabetic mice | 100 mg/kg/d (p.o.) for 12 weeks | Inhibited IKK/NF-κB signaling and NOX2/NOX4-mediated oxidative stress | **[224]** |
|  |  |  | Palmitate- or high glucose/LPS-stimulated MES13 mouse mesangial cells | 20 μM for 24 h |  |  |

**Table S1. Flavonoids and diabetic nephropathy (continued)**

| **Subclass** | **Compounds** | **Experimental setting** | **Model** | **Treatment methods** | **Molecular mechanisms** | **Reference** |
| --- | --- | --- | --- | --- | --- | --- |
| Chalcone glycoside | Marein | In vivo and in vitro | db/db mice | 50 mg/kg/d for 12 weeks | Inhibited SGLT2 and activated AMPK/ACC signaling | [225] |
|  |  |  | High glucose-induced HK-2 cells | 33, 66, or 132 μM for 6 h |  |  |
| **Flavanonol** | Astilbin | In vitro | High glucose-induced HBZY-1 cells | 10, 20 µg/mL for 24 h | Suppressed the TLR4/MyD88/NF-κB pathway | [226] |
|  |  | In vivo | STZ-induced diabetic rats | 2.5 or 5 mg/kg/d (i.g.) for 8 weeks | Inhibited TGF-β1 and CTGF expression | [227] |
|  |  | In vitro | High-glucose-induced HK-2 cells | 10, 20 μg/mL for 24 h | Activated PI3K/Akt signaling to inhibit autophagy and apoptosis | [228] |
|  | Dihydromyricetin | In vitro | High glucose‐induced HMCs | 10，20 µM for 24 h | Activated the Nrf2/HO-1 pathway, inhibited NOX2/NOX4 activity, and thereby alleviated oxidative stress | [229] |
| **Flavanonol** | Dihydromyricetin | In vivo and in vitro | Uninephrectomy+STZ induced DN in SD rats | 100 mg/kg/d (s.c.) for 10 weeks | Promoted autophagy and attenuates renal interstitial fibrosis by regulating miR-155-5p/PTEN pathway | [230] |
|  |  |  | High glucose‐induced NRK-52E | 1 µM for 24 or 48 h |  |  |
|  |  | In vivo and in vitro | HFD/STZ-induced diabetic kidney disease in C57BL/6J mice | 100, 200 mg/kg/d (p.o.) for 10 weeks (6 d/week) | Directly targeted SphK1 and suppressed SphK1/S1P/NF-κB signaling | [231] |
|  |  |  | High glucose-induced primary rat glomerular mesangial cells (GMCs) | 10, 20, 40 μM for 36 h |  |  |
|  | Taxifolin | In vivo and in vitro | HFD/STZ-induced DN in SD rats | 25, 50, 100 mg/kg/d for 12 weeks | Suppressed ROS generation and NLRP3 inflammasome activation | [232] |
|  |  |  | High glucose-induced HBZY-1 cells and HK-2 cells | 5, 10, 20 μM for 72 h |  |  |

**Table S1. Flavonoids and diabetic nephropathy (continued)**

| **Subclass** | **Compounds** | **Experimental setting** | **Model** | **Treatment methods** | **Molecular mechanisms** | **Reference** |
| --- | --- | --- | --- | --- | --- | --- |
| Anthocyanin | Cyanidin-3-O-glucoside | In vivo | db/db mice | 10, 20 mg/kg/d (i.p.) for 12 weeks | Enhanced the renal glutathione pool by upregulating GCLC/GCLM | [233] |
|  |  | In vivo | STZ-induced DN in SD rats | 10, 20 mg/kg/d (i.g.) for 8 weeks | Attenuated oxidative stress and inflammation and downregulated TGF-β1/Smad2/3 signaling | [234] |
|  |  | In vitro | High glucose-induced HK-2 human proximal tubular epithelial cells | 50 μM for 1 h | Activated PPARα/LXRα/ABCA1-mediated cholesterol efflux and inhibited NF-κB-mediated inflammation | [235] |
|  |  | In vitro | High glucose-induced MPC-5 cells | 50 μM for 24 h | Activated SIRT1/AMPK signaling, promoted autophagy, and inhibited apoptosis and EMT | [236] |
|  |  | In vivo | db/db mice | 10 mg/kg/d (i.g.) for 12 weeks | Modulated amino acid metabolism, particularly renal taurine/hypotaurine metabolism | [237] |
|  | Purple corn anthocyanins | In vitro | High glucose-induced human renal mesangial cells | 20 μg/mL for 3 days | Suppressed TGF-β/SMAD–NF-κB signaling crosstalk and reduced mesangial proliferation, inflammation, and extracellular matrix accumulation | **[238]** |
|  |  |  |  |  |  |  |
|  |  |  |  |  |  |  |

**Table S1. Flavonoids and diabetic nephropathy (continued)**

| **Subclass** | **Compounds** | **Experimental setting** | **Model** | **Treatment methods** | **Molecular mechanisms** | **Reference** |
| --- | --- | --- | --- | --- | --- | --- |
| Flavonoid glycoside | Complanatoside A | In vivo and in vitro | High glucose-induced SV40-MES 13 cells | 40 μM for 24 h | Suppressed glomerular ECM accumulation, inflammation and fibrotic remodeling, with modulation of inflammation/ECM-related targets | [239] |
|  |  |  | HFD/STZ-induced DN in C57BL/6 mice | 5, 10 mg/kg/d (i.p.) for 8 weeks |  |  |
| Prenylated flavonoid | Isoangustone A | In vitro | High-glucose-induced HRMCs | 1, 10, 20 μM for 72 h | Inhibited TGF-β/SMAD and NF-κB signaling | [240] |
| Biflavone | Ginkgetin | In vitro | High-glucose-induced HBZY-1 cells | 20 μM for 24 h | Activated AMPK/mTOR-mediated autophagy | [241] |
|  | Cupressuflavone | In vivo and in vitro | STZ-induced diabetic nephropathy in rats | 25 mg/kg (p.o.) for 4 weeks | Activated Nrf2 signaling and suppressed NF-κB activation | [242] |
|  |  |  | High glucose-induced NRK-52E cells | 25, 50, 100 μM |  |  |
| Flavonolignan | Silibinin | In vivo and in vitro | HFD-induced renal fibrosis in C57BL/6 mice | Silibinin (dose NR) for 6 weeks | Enhanced valsartan-mediated inhibition of TGF-β1/Smad2/3 signaling | [243] |
|  |  |  | TGF-β1-induced HK-2 cells | Silibinin 30 μM for 72 h |  |  |
|  |  | In vivo and in vitro | High glucose-induced mouse podocytes | 10 μM overnight pretreatment, followed by 24 h cotreatment | Inhibited Nox4/NADPH oxidase-derived ROS and podocyte apoptosis | [244] |
|  |  |  | OVE26 type 1 diabetic mice | 100 mg/kg (i.p.) for 6 weeks |  |  |
|  |  |  |  |  |  |  |

**Table S1. Flavonoids and diabetic nephropathy (continued)**

| **Subclass** | **Compounds** | **Experimental setting** | **Model** | **Treatment methods** | **Molecular mechanisms** | **Reference** |
| --- | --- | --- | --- | --- | --- | --- |
| Flavonolignan | Silibinin | In vivo and in vitro | TGF-β1-induced human renal glomerular endothelial cells (HRGECs) | 50 μM for 72 h | Inhibited NF-κB signaling and reduced fibrotic marker expression | [245] |
|  |  |  | HFD-induced hyperglycemic C57BL/6J mice | 50, 100 mg/kg/d for 6 weeks |  |  |
|  | silymarin | In vitro | High glucose-induced MPC-5 cells | 16, 32, 64 μg/mL for 12 h | Restored podocin and nephrin expression and reduced inflammatory cytokine expression | [246] |
|  |  | In vivo | Alloxan-induced diabetic Wistar rats | 200 mg/kg/d (p.o.) for 9 weeks | Restored renal antioxidant enzyme activity and expression and reduced oxidative injury | [247] |
|  |  | In vivo and in vitro | HFD-fed C57BL/6 mice | 30 mg/kg/d (p.o.) for 4 weeks | Normalized renal lipid metabolism and mitochondrial biogenesis and reduced oxidative stress | [248] |
|  |  |  | Palmitic acid-induced HK-2 cells | 25 μM for 36 h |  |  |
| Prenylated chalcone | Xanthohumol | In vivo and in vitro | STZ-induced male C57BL/6 mice | 25 mg/kg/d (i.p.) for 20 weeks | Nrf2-mediated attenuation of oxidative stress | [249] |
|  |  |  | High glucose-induced human renal glomerular endothelial cells (GECs) and HK-2 cells | 50 μM for 48 h |  |  |
|  |  |  |  |  |  |  |

**Table S1. Flavonoids and diabetic nephropathy (continued)**

| **Subclass** | **Compounds** | **Experimental setting** | **Model** | **Treatment methods** | **Molecular mechanisms** | **Reference** |
| --- | --- | --- | --- | --- | --- | --- |
| Flavone glycoside + flavone | Baicalin + chrysin mixture | In vivo and in vitro | STZ-induced diabetic Wistar rats | Baicalin 75 mg/kg + chrysin 10 mg/kg (p.o.); duration NR | Suppressed AGEs/RAGE signaling and reduced oxidative stress and inflammation | **[250]** |
|  |  |  | Methylglyoxal-induced NRK-52E rat renal tubular epithelial cells | Baicalin 30 μg/mL + chrysin 15 μg/mL for 1 h |  |  |
| Flavanol | Grape seed procyanidins | In vivo and in vitro | db/db mice | 30 mg/kg/d (p.o.) for 12 weeks | Suppressed oxidative stress and renal tubular apoptosis by inhibiting TXNIP expression, p38 MAPK/ERK1/2 phosphorylation, and mitochondrial apoptotic signaling | **[251]** |
| Anthocyanin | Cyanidin-3-O-glucoside |  | High glucose-induced HK-2 human proximal tubular epithelial cells | 50 μM for 48 h |  |  |
|  |  |  |  |  |  |  |

**Reference**

[1] W. Luo, X. Chen, L. Ye, X. Chen, W. Jia, Y. Zhao, et al., Kaempferol attenuates streptozotocin-induced diabetic nephropathy by downregulating TRAF6 expression: The role of TRAF6 in diabetic nephropathy, J. Ethnopharmacol. 268 (2021) 113553.

[2] H. Sheng, D. Zhang, J. Zhang, Y. Zhang, Z. Lu, W. Mao, et al., Kaempferol attenuated diabetic nephropathy by reducing apoptosis and promoting autophagy through AMPK/mTOR pathways, Front. Med. (Lausanne) 9 (2022) 986825.

[3] C. Xia, J. Zhang, H. Chen, W. Jiang, S. Zhou, H. Zheng, et al., Kaempferol improves mitochondrial homeostasis via mitochondrial dynamics and mitophagy in diabetic kidney disease, Int. Immunopharmacol. 162 (2025) 115121.

[4] A.S. Alshehri, Kaempferol attenuates diabetic nephropathy in streptozotocin-induced diabetic rats by a hypoglycaemic effect and concomitant activation of the Nrf-2/Ho-1/antioxidants axis, Arch. Physiol. Biochem. 129 (2023) 984–997.

[5] X.C. Jiao, Y. Li, D. Wu, X.G. Zhang, F. Hou, The protective effect of kaempferol on high glucose-stimulated renal tubular epithelial cells, BMC Nephrol. 26 (2025) 477.

[6] D. Sharma, P. Gondaliya, V. Tiwari, K. Kalia, Kaempferol attenuates diabetic nephropathy by inhibiting RhoA/Rho-kinase mediated inflammatory signalling, Biomed. Pharmacother. 109 (2019) 1610–1619.

[7] D. Sharma, R. Kumar Tekade, K. Kalia, Kaempferol in ameliorating diabetes-induced fibrosis and renal damage: An in vitro and in vivo study in diabetic nephropathy mice model, Phytomedicine 76 (2020) 153235.

[8] Q. Feng, Y. Yang, Y. Qiao, Y. Zheng, X. Yu, F. Liu, et al., Quercetin Ameliorates Diabetic Kidney Injury by Inhibiting Ferroptosis via Activating Nrf2/HO-1 Signaling Pathway, Am. J. Chin. Med. 51 (2023) 997–1018.

[9] L. Zhang, X. Wang, L. Chang, Y. Ren, M. Sui, Y. Fu, et al., Quercetin improves diabetic kidney disease by inhibiting ferroptosis and regulating the Nrf2 in streptozotocin-induced diabetic rats, Ren. Fail. 46 (1) (2024) 2327495.

[10] X. Zhao, S. Wang, X. He, W. Wei, K. Huang, Quercetin prevents the USP22-Snail1 signaling pathway to ameliorate diabetic tubulointerstitial fibrosis, Food Funct. 15 (2024) 11990–12006.

[11] F. Liu, Q. Feng, M. Yang, Y. Yang, J. Nie, S. Wang, Quercetin prevented diabetic nephropathy by inhibiting renal tubular epithelial cell apoptosis via the PI3K/AKT pathway, Phytother. Res. 38 (2024) 3594–3606.

[12] Y. Liu, Y. Li, L. Xu, J. Shi, X. Yu, X. Wang, et al., Quercetin Attenuates Podocyte Apoptosis of Diabetic Nephropathy Through Targeting EGFR Signaling, Front. Pharmacol. 12 (2021) 792777.

[13] M. Anjaneyulu, K. Chopra, Quercetin, an anti-oxidant bioflavonoid, attenuates diabetic nephropathy in rats, Clin. Exp. Pharmacol. Physiol. 31 (2004) 244–248.

[14] D.Q. Tang, Y.Q. Wei, X.X. Yin, Q. Lu, H.H. Hao, Y.P. Zhai, et al., In vitro suppression of quercetin on hypertrophy and extracellular matrix accumulation in rat glomerular mesangial cells cultured by high glucose, Fitoterapia 82 (2011) 920–926.

[15] P.B. Lai, L. Zhang, L.Y. Yang, Quercetin ameliorates diabetic nephropathy by reducing the expressions of transforming growth factor-β1 and connective tissue growth factor in streptozotocin-induced diabetic rats, Ren. Fail. 34 (2012) 83–87.

[16] P. Chen, Q. Shi, X. Xu, Y. Wang, W. Chen, H. Wang, Quercetin suppresses NF-κB and MCP-1 expression in a high glucose-induced human mesangial cell proliferation model, Int. J. Mol. Med. 30 (2012) 119–125.

[17] I.B. Gomes, M.L. Porto, M.C. Santos, B.P. Campagnaro, T.M. Pereira, S.S. Meyrelles, et al., Renoprotective, anti-oxidative and anti-apoptotic effects of oral low-dose quercetin in the C57BL/6J model of diabetic nephropathy, Lipids Health Dis. 13 (2014) 184.

[18] Q. Lu, X.J. Ji, Y.X. Zhou, X.Q. Yao, Y.Q. Liu, F. Zhang, et al., Quercetin inhibits the mTORC1/p70S6K signaling-mediated renal tubular epithelial-mesenchymal transition and renal fibrosis in diabetic nephropathy, Pharmacol. Res. 99 (2015) 237–247.

[19] I.B. Gomes, M.L. Porto, M.C. Santos, B.P. Campagnaro, A.L. Gava, S.S. Meyrelles, et al., The protective effects of oral low-dose quercetin on diabetic nephropathy in hypercholesterolemic mice, Front. Physiol. 6 (2015) 247.

[20] H. Elbe, N. Vardi, M. Esrefoglu, B. Ates, S. Yologlu, C. Taskapan, Amelioration of streptozotocin-induced diabetic nephropathy by melatonin, quercetin, and resveratrol in rats, Hum. Exp. Toxicol. 34 (2015) 100–113.

[21] F. Gao, X. He, S. Liang, S. Liu, H. Liu, Q. He, et al., Quercetin ameliorates podocyte injury via inhibition of oxidative stress and the TGF-β1/Smad pathway in DN rats, RSC Adv. 8 (2018) 35413–35421.

[22] D. Lei, L. Chengcheng, Q. Xuan, C. Yibing, W. Lei, Y. Hao, et al., Quercetin inhibited mesangial cell proliferation of early diabetic nephropathy through the Hippo pathway, Pharmacol. Res. 146 (2019) 104320.

[23] H.M. Abdou, H.A.E. Abd Elkader, The potential therapeutic effects of Trifolium alexandrinum extract, hesperetin and quercetin against diabetic nephropathy via attenuation of oxidative stress, inflammation, GSK-3β and apoptosis in male rats, Chem. Biol. Interact. 352 (2022) 109781.

[24] T. Yang, Y. Hu, W. Jiang, J. Pang, Y. Zhou, H. Zhang, et al., YY1 was indispensable for the alleviation of quercetin on diabetic nephropathy-associated tubulointerstitial inflammation, Phytomedicine 111 (2023) 154659.

[25] A. Hossein, G. Firouzeh, K. Zeinab, D. Gholamreza, Quercetin prevents kidney against diabetes mellitus (type 1) in rats by inhibiting TGF-β/apelin gene expression, Mol. Biol. Rep. 51 (2024) 677.

[26] A. Abudoureyimu, C. Chen, Y. Hu, D. Nuermaimaiti, T. Liu, Quercetin alleviates diabetic nephropathy by inhibiting M1 macrophage polarization via targeting NLRC5/NLRP3 pathway, Cell. Immunol. 414 (2025) 104997.

[27] B. Hou, Y. Li, J. Tang, X. Cao, S. Zhang, Y. Cai, et al., Targeting FoxO1-STX17-mediated autophagic flux: Quercetin's therapeutic mechanism in diabetic nephropathy, Eur. J. Pharmacol. 1008 (2025) 178309.

[28] W. Yong, Z. Li, W. Xu, D. Gao, B. Sha, Y. Jin, et al., Quercetin ameliorates renal injury by promoting UCP1-mediated alleviation of lipid accumulation in diabetic kidney disease, Phytomedicine 147 (2025) 157213.

[29] H. Wan, Y. Wang, Q. Pan, X. Chen, S. Chen, X. Li, et al., Quercetin attenuates the proliferation, inflammation, and oxidative stress of high glucose-induced human mesangial cells by regulating the miR-485-5p/YAP1 pathway, Int. J. Immunopathol. Pharmacol. 36 (2022) 20587384211066440.

[30] F. Tong, S. Liu, B. Yan, X. Li, S. Ruan, S. Yang, Quercetin nanoparticle complex attenuated diabetic nephropathy via regulating the expression level of ICAM-1 on endothelium, Int. J. Nanomedicine 12 (2017) 7799–7813.

[31] L. Tang, K. Li, Y. Zhang, H. Li, A. Li, Y. Xu, et al., Quercetin liposomes ameliorate streptozotocin-induced diabetic nephropathy in diabetic rats, Sci. Rep. 10 (2020) 2440.

[32] H.S. Dh, R. Sultana, A. Prabhu, R.P. S, S. Mohanto, V. Subramaniyan, Biomedicine and pharmacotherapeutic effectiveness of combinatorial atorvastatin and quercetin on diabetic nephropathy: An in vitro study, Biomed. Pharmacother. 174 (2024) 116533.

[33] X. Guo, S. Wen, J. Wang, X. Zeng, H. Yu, Y. Chen, et al., Senolytic combination of dasatinib and quercetin attenuates renal damage in diabetic kidney disease, Phytomedicine 130 (2024) 155705.

[34] X. Zhu, C. Zhang, L. Liu, L. Xu, L. Yao, Senolytic combination of dasatinib and quercetin protects against diabetic kidney disease by activating autophagy to alleviate podocyte dedifferentiation via the Notch pathway, Int. J. Mol. Med. 53 (3) (2024) 26.

[35] M. Gan, Z. Lin, J. Ma, N. Li, B. Wu, A Network Pharmacology-Based Investigation into the Mechanism of Quercetin Combined with Rosuvastatin in Delaying Diabetic Nephropathy via Inhibiting NRK-52E Cell Ferroptosis, Diabetes Metab. Syndr. Obes. 18 (2025) 2681–2694.

[36] C. Wang, Y. Pan, Q.Y. Zhang, F.M. Wang, L.D. Kong, Quercetin and allopurinol ameliorate kidney injury in STZ-treated rats with regulation of renal NLRP3 inflammasome activation and lipid accumulation, PLoS One 7 (2012) e38285.

[37] X. Jiang, J. Yu, X. Wang, J. Ge, N. Li, Quercetin improves lipid metabolism via SCAP-SREBP2-LDLr signaling pathway in early stage diabetic nephropathy, Diabetes Metab. Syndr. Obes. 12 (2019) 827–839.

[38] X. Qian, S. Lin, J. Li, C. Jia, Y. Luo, R. Fan, et al., Fisetin Ameliorates Diabetic Nephropathy-Induced Podocyte Injury by Modulating Nrf2/HO-1/GPX4 Signaling Pathway, Evid. Based Complement. Alternat. Med. 2023 (2023) 9331546.

[39] W. Dong, C. Jia, J. Li, Y. Zhou, Y. Luo, J. Liu, et al., Fisetin Attenuates Diabetic Nephropathy-Induced Podocyte Injury by Inhibiting NLRP3 Inflammasome, Front. Pharmacol. 13 (2022) 783706.

[40] C. Ge, M. Xu, Y. Qin, T. Gu, D. Lou, Q. Li, et al., Fisetin supplementation prevents high fat diet-induced diabetic nephropathy by repressing insulin resistance and RIP3-regulated inflammation, Food Funct. 10 (2019) 2970–2985.

[41] T.F. Zou, Z.G. Liu, P.C. Cao, S.H. Zheng, W.T. Guo, T.X. Wang, et al., Fisetin treatment alleviates kidney injury in mice with diabetes-exacerbated atherosclerosis through inhibiting CD36/fibrosis pathway, Acta Pharmacol. Sin. 44 (2023) 2065–2074.

[42] Y.Q. Ke, C. Liu, J.B. Hao, L. Lu, N.N. Lu, Z.K. Wu, et al., Morin inhibits cell proliferation and fibronectin accumulation in rat glomerular mesangial cells cultured under high glucose condition, Biomed. Pharmacother. 84 (2016) 622–627.

[43] X. Xin, X.H. Li, J.Z. Wu, K.H. Chen, Y. Liu, C.J. Nie, et al., Pentamethylquercetin ameliorates fibrosis in diabetic Goto-Kakizaki rat kidneys and mesangial cells with suppression of TGF-β/Smads signaling, Eur. J. Pharmacol. 713 (2013) 6–15.

[44] M. Matboli, D. Ibrahim, A.H. Hasanin, M.K. Hassan, E.K. Habib, M.M. Bekhet, et al., Epigenetic modulation of autophagy genes linked to diabetic nephropathy by administration of isorhamnetin in Type 2 diabetes mellitus rats, Epigenomics 13 (2021) 187–202.

[45] B. Liu, C. Deng, P. Tan, Ombuin ameliorates diabetic nephropathy in rats by anti-inflammation and antifibrosis involving Notch 1 and PPAR γ signaling pathways, Drug Dev. Res. 83 (2022) 1270–1280.

[46] S.H. Aladaileh, F.K. Al-Swailmi, M.H. Abukhalil, M.H. Shalayel, Galangin protects against oxidative damage and attenuates inflammation and apoptosis via modulation of NF-κB p65 and caspase-3 signaling molecules in a rat model of diabetic nephropathy, J. Physiol. Pharmacol. 72 (1) (2021) 35–44.

[47] R. Keyhanmanesh, G. Hamidian, H. Lotfi, Z. Zavari, M. Seyfollahzadeh, A. Ghadiri, et al., Troxerutin affects nephropathy signaling events in the kidney of type-1 diabetic male rats, Avicenna J. Phytomed. 12 (2022) 109–115.

[48] N. Yuan, J. Diao, J. Dong, Y. Yan, Y. Chen, S. Yan, et al., Targeting ROCK1 in diabetic kidney disease: Unraveling mesangial fibrosis mechanisms and introducing myricetin as a novel antagonist, Biomed. Pharmacother. 171 (2024) 116208.

[49] N. Kandasamy, N. Ashokkumar, Renoprotective effect of myricetin restrains dyslipidemia and renal mesangial cell proliferation by the suppression of sterol regulatory element binding proteins in an experimental model of diabetic nephropathy, Eur. J. Pharmacol. 743 (2014) 53–62.

[50] F. Ozcan, A. Ozmen, B. Akkaya, Y. Aliciguzel, M. Aslan, Beneficial effect of myricetin on renal functions in streptozotocin-induced diabetes, Clin. Exp. Med. 12 (2012) 265–272.

[51] N. Yuan, Y. Chen, Y. Yan, F. Wang, X. Xu, M. Wang, et al., Myricetin alleviates renal tubular epithelial-mesenchymal transition via NOX4/NF-κB/snail axis in diabetic nephropathy based on network pharmacology analysis, Heliyon 10 (2024) e35234.

[52] W.L. Xu, P.P. Zhou, X. Yu, T. Tian, J.J. Bao, C.R. Ni, et al., Myricetin induces M2 macrophage polarization to alleviate renal tubulointerstitial fibrosis in diabetic nephropathy via PI3K/Akt pathway, World J. Diabetes 15 (2024) 105–125.

[53] J. Zhou, S. Zhang, X. Sun, Y. Lou, J. Bao, J. Yu, Hyperoside ameliorates diabetic nephropathy induced by STZ via targeting the miR-499-5p/APC axis, J. Pharmacol. Sci. 146 (2021) 10–20.

[54] J. Zhang, H. Fu, Y. Xu, Y. Niu, X. An, Hyperoside reduces albuminuria in diabetic nephropathy at the early stage through ameliorating renal damage and podocyte injury, J. Nat. Med. 70 (2016) 740–748.

[55] J. Liu, Y. Zhang, H. Sheng, C. Liang, H. Liu, J.A. Moran Guerrero, et al., Hyperoside Suppresses Renal Inflammation by Regulating Macrophage Polarization in Mice With Type 2 Diabetes Mellitus, Front. Immunol. 12 (2021) 733808.

[56] L. Zhang, Q. Dai, L. Hu, H. Yu, J. Qiu, J. Zhou, et al., Hyperoside Alleviates High Glucose-Induced Proliferation of Mesangial Cells through the Inhibition of the ERK/CREB/miRNA-34a Signaling Pathway, Int. J. Endocrinol. 2020 (2020) 1361924.

[57] X. An, L. Zhang, Y. Yuan, B. Wang, Q. Yao, L. Li, et al., Hyperoside pre-treatment prevents glomerular basement membrane damage in diabetic nephropathy by inhibiting podocyte heparanase expression, Sci. Rep. 7 (2017) 6413.

[58] K. Zhang, M. Li, K. Yin, M. Wang, Q. Dong, Z. Miao, et al., Hyperoside mediates protection from diabetes kidney disease by regulating ROS-ERK signaling pathway and pyroptosis, Phytother. Res. 37 (2023) 5871–5882.

[59] W. Jiang, R. Wang, D. Liu, M. Zuo, C. Zhao, T. Zhang, et al., Protective Effects of Kaempferitrin on Advanced Glycation End Products Induce Mesangial Cell Apoptosis and Oxidative Stress, Int. J. Mol. Sci. 19 (11) (2018) 3334.

[60] C. Qiao, W. Ye, S. Li, H. Wang, X. Ding, Icariin modulates mitochondrial function and apoptosis in high glucose-induced glomerular podocytes through G protein-coupled estrogen receptors, Mol. Cell. Endocrinol. 473 (2018) 146–155.

[61] Z. Jia, K. Wang, Y. Zhang, Y. Duan, K. Xiao, S. Liu, et al., Icariin Ameliorates Diabetic Renal Tubulointerstitial Fibrosis by Restoring Autophagy via Regulation of the miR-192-5p/GLP-1R Pathway, Front. Pharmacol. 12 (2021) 720387.

[62] M.Y. Qi, C. Kai, H.R. Liu, Y.H. Su, S.Q. Yu, Protective effect of Icariin on the early stage of experimental diabetic nephropathy induced by streptozotocin via modulating transforming growth factor β1 and type IV collagen expression in rats, J. Ethnopharmacol. 138 (2011) 731–736.

[63] Y.C. Li, X.S. Ding, H.M. Li, C. Zhang, Icariin attenuates high glucose-induced type IV collagen and fibronectin accumulation in glomerular mesangial cells by inhibiting transforming growth factor-β production and signalling through G protein-coupled oestrogen receptor 1, Clin. Exp. Pharmacol. Physiol. 40 (2013) 635–643.

[64] W. Tian, H. Lei, R. Guan, Y. Xu, H. Li, L. Wang, et al., Icariside II ameliorates diabetic nephropathy in streptozotocin-induced diabetic rats, Drug Des. Devel. Ther. 9 (2015) 5147–5157.

[65] K. Wang, X. Zheng, Z. Pan, W. Yao, X. Gao, X. Wang, et al., Icariin Prevents Extracellular Matrix Accumulation and Ameliorates Experimental Diabetic Kidney Disease by Inhibiting Oxidative Stress via GPER Mediated p62-Dependent Keap1 Degradation and Nrf2 Activation, Front. Cell Dev. Biol. 8 (2020) 559.

[66] M.Y. Qi, Y.H. He, Y. Cheng, Q. Fang, R.Y. Ma, S.J. Zhou, et al., Icariin ameliorates streptozocin-induced diabetic nephropathy through suppressing the TLR4/NF-κB signal pathway, Food Funct. 12 (2021) 1241–1251.

[67] X. Ding, H. Zhao, C. Qiao, Icariin protects podocytes from NLRP3 activation by Sesn2-induced mitophagy through the Keap1-Nrf2/HO-1 axis in diabetic nephropathy, Phytomedicine 99 (2022) 154005.

[68] L. Zang, F. Gao, A. Huang, Y. Zhang, Y. Luo, L. Chen, et al., Icariin inhibits epithelial mesenchymal transition of renal tubular epithelial cells via regulating the miR-122-5p/FOXP2 axis in diabetic nephropathy rats, J. Pharmacol. Sci. 148 (2022) 204–213.

[69] B.L. Su, L.L. Wang, L.Y. Zhang, S. Zhang, Q. Li, G.Y. Chen, Potential role of microRNA-503 in Icariin-mediated prevention of high glucose-induced endoplasmic reticulum stress, World J. Diabetes 14 (2023) 1234–1248.

[70] W. Yao, R. Tao, Y. Xu, Z.S. Chen, X. Ding, L. Wan, AR/RKIP pathway mediates the inhibitory effects of icariin on renal fibrosis and endothelial-to-mesenchymal transition in type 2 diabetic nephropathy, J. Ethnopharmacol. 320 (2024) 117414.

[71] K. Wang, M. Hou, C. Qiao, Y. Duan, R. Tao, X. Wang, et al., Icariin alleviates diabetic renal interstitial fibrosis aggravation by inhibiting miR-320a-3p targeting BMP6, J. Pharmacol. Sci. 154 (2024) 316–325.

[72] N. Kamalakkannan, P. Stanely Mainzen Prince, The influence of rutin on the extracellular matrix in streptozotocin-induced diabetic rat kidney, J. Pharm. Pharmacol. 58 (2006) 1091–1098.

[73] H.H. Hao, Z.M. Shao, D.Q. Tang, Q. Lu, X. Chen, X.X. Yin, et al., Preventive effects of rutin on the development of experimental diabetic nephropathy in rats, Life Sci. 91 (2012) 959–967.

[74] D. Ganesan, A. Holkar, A. Albert, E. Paul, J. Mariakuttikan, G. Sadasivam Selvam, Combination of ramipril and rutin alleviate alloxan induced diabetic nephropathy targeting multiple stress pathways in vivo, Biomed. Pharmacother. 108 (2018) 1338–1346.

[75] X. Wang, X. Zhao, T. Feng, G. Jin, Z. Li, Rutin Prevents High Glucose-Induced Renal Glomerular Endothelial Hyperpermeability by Inhibiting the ROS/Rhoa/ROCK Signaling Pathway, Planta Med. 82 (2016) 1252–1257.

[76] D. Ganesan, A. Albert, E. Paul, K. Ananthapadmanabhan, R. Andiappan, S.G. Sadasivam, Rutin ameliorates metabolic acidosis and fibrosis in alloxan induced diabetic nephropathy and cardiomyopathy in experimental rats, Mol. Cell. Biochem. 471 (2020) 41–50.

[77] R.A. Zaghloul, A.M. Abdelghany, Y.A. Samra, Rutin and selenium nanoparticles protected against STZ-induced diabetic nephropathy in rats through downregulating Jak-2/Stat3 pathway and upregulating Nrf-2/HO-1 pathway, Eur. J. Pharmacol. 933 (2022) 175289.

[78] L. Wu, L. Li, X. Wang, H. Wu, M. Li, Y. Wang, et al., The inhibition of rutin on Src kinase blocks high glucose-induced EGFR/ERK transactivation in diabetic nephropathy by integrative approach of network pharmacology and experimental verification, Phytomedicine 135 (2024) 156220.

[79] B. Gong, X. Gou, T. Han, Y. Qi, X. Ji, J. Bai, Protective effects of rutin on kidney in type 1 diabetic mice, Pak. J. Pharm. Sci. 33 (2020) 597–603.

[80] R. Dong, X. Zhang, Y. Liu, T. Zhao, Z. Sun, P. Liu, et al., Rutin alleviates EndMT by restoring autophagy through inhibiting HDAC1 via PI3K/AKT/mTOR pathway in diabetic kidney disease, Phytomedicine 112 (2023) 154700.

[81] M.Y. Sun, H.J. Ye, C. Zheng, Z.J. Jin, Y. Yuan, H.B. Weng, Astragalin ameliorates renal injury in diabetic mice by modulating mitochondrial quality control via AMPK-dependent PGC1α pathway, Acta Pharmacol. Sin. 44 (2023) 1676–1686.

[82] R. Yin, Y. Wang, P. Zhang, M. Sun, Z. Jin, S. Ma, et al., Promoting ubiquitin-dependent Drp1 degradation contributes to the protective effect of Astragalin against diabetic renal fibrosis, Biochem. Pharmacol. 241 (2025) 117158.

[83] C. Xuan, D. Chen, S. Zhang, C. Li, Q. Fang, D. Chen, et al., Isoquercitrin Alleviates Diabetic Nephropathy by Inhibiting STAT3 Phosphorylation and Dimerization, Adv. Sci. (Weinh.) 12 (2025) e2414587.

[84] W. Zhang, Y. Zhang, W. Lv, Z. Kong, F. Wang, Y. Wang, Isoquercitrin improves diabetes nephropathy by inhibiting the sodium-glucose co-transporter-2 pathway, Biochem. Biophys. Res. Commun. 744 (2025) 151142.

[85] S. Li, H. Xiao, X. Zhang, L. Pan, M. Wen, L. Liang, et al., Reynoutrin activates Sirt1/Foxo3a and Nrf2 pathway via targeting Fyn to ameliorate diabetic nephropathy, Phytomedicine 146 (2025) 157158.

[86] X. Zhang, Z. Huo, X. Jia, Y. Xiong, B. Li, L. Zhang, et al., (+)-Catechin ameliorates diabetic nephropathy injury by inhibiting endoplasmic reticulum stress-related NLRP3-mediated inflammation, Food Funct. 15 (2024) 5450–5465.

[87] Y. Ni, D. Zhu, C. Chen, F. Wang, Y. Miu, W. Zhang, (+)-Catechins Play a Protective Role in Diabetic Kidney Disease by Alleviating EMT through Multiple Pathways, Mol. Nutr. Food Res. 68 (2024) e2400387.

[88] D. Zhu, L. Wang, Q. Zhou, S. Yan, Z. Li, J. Sheng, et al., (+)-Catechin ameliorates diabetic nephropathy by trapping methylglyoxal in type 2 diabetic mice, Mol. Nutr. Food Res. 58 (2014) 2249–2260.

[89] S.P. Ertürküner, M. Başar, M. Tunçdemir, İ. Seçkin, The comparative effects of perindopril and catechin on mesangial matrix and podocytes in the streptozotocin induced diabetic rats, Pharmacol. Rep. 66 (2014) 279–287.

[90] X. Cai, L. Bao, J. Ren, Y. Li, Z. Zhang, Grape seed procyanidin B2 protects podocytes from high glucose-induced mitochondrial dysfunction and apoptosis via the AMPK-SIRT1-PGC-1α axis in vitro, Food Funct. 7 (2016) 805–815.

[91] Y. Zhou, B.Y. Li, X.L. Li, Y.J. Wang, Z. Zhang, F. Pei, et al., Restoration of Mimecan Expression by Grape Seed Procyanidin B2 Through Regulation of Nuclear Factor-kappaB in Mice With Diabetic Nephropathy, Iran. J. Kidney Dis. 10 (2016) 325–331.

[92] Z. Zhang, B.Y. Li, X.L. Li, M. Cheng, F. Yu, W.D. Lu, et al., Proteomic analysis of kidney and protective effects of grape seed procyanidin B2 in db/db mice indicate MFG-E8 as a key molecule in the development of diabetic nephropathy, Biochim. Biophys. Acta 1832 (2013) 805–816.

[93] A.A. Sayed, Thymoquinone and proanthocyanidin attenuation of diabetic nephropathy in rats, Eur. Rev. Med. Pharmacol. Sci. 16 (2012) 808–815.

[94] D. Li, T. Zhao, J. Meng, Y. Jing, F. Jia, P. He, Procyanidin B2 inhibits high glucose‑induced epithelial‑mesenchymal transition in HK‑2 human renal proximal tubular epithelial cells, Mol. Med. Rep. 12 (2015) 8148–8154.

[95] Y.A. Lee, Y.J. Kim, E.J. Cho, T. Yokozawa, Ameliorative effects of proanthocyanidin on oxidative stress and inflammation in streptozotocin-induced diabetic rats, J. Agric. Food Chem. 55 (2007) 9395–9400.

[96] Y. Ding, H. Li, Y. Li, D. Liu, L. Zhang, T. Wang, et al., Protective Effects of Grape Seed Proanthocyanidins on the Kidneys of Diabetic Rats through the Nrf2 Signalling Pathway, Evid. Based Complement. Alternat. Med. 2020 (2020) 5205903.

[97] Z. Gao, G. Liu, Z. Hu, W. Shi, B. Chen, P. Zou, et al., Grape seed proanthocyanidins protect against streptozotocin‑induced diabetic nephropathy by attenuating endoplasmic reticulum stress‑induced apoptosis, Mol. Med. Rep. 18 (2018) 1447–1454.

[98] B.Y. Li, M. Cheng, H.Q. Gao, Y.B. Ma, L. Xu, X.H. Li, et al., Back-regulation of six oxidative stress proteins with grape seed proanthocyanidin extracts in rat diabetic nephropathy, J. Cell. Biochem. 104 (2008) 668–679.

[99] X. Li, Y. Xiao, H. Gao, B. Li, L. Xu, M. Cheng, et al., Grape seed proanthocyanidins ameliorate diabetic nephropathy via modulation of levels of AGE, RAGE and CTGF, Nephron Exp. Nephrol. 111 (2009) e31–E41.

[100] P. Gong, P. Wang, S. Pi, Y. Guo, S. Pei, W. Yang, et al., Proanthocyanidins Protect Against Cadmium-Induced Diabetic Nephropathy Through p38 MAPK and Keap1/Nrf2 Signaling Pathways, Front. Pharmacol. 12 (2021) 801048.

[101] Y.J. Liang, J.H. Jian, Y.C. Liu, S.J. Juang, K.G. Shyu, L.P. Lai, et al., Advanced glycation end products-induced apoptosis attenuated by PPARdelta activation and epigallocatechin gallate through NF-kappaB pathway in human embryonic kidney cells and human mesangial cells, Diabetes Metab. Res. Rev. 26 (2010) 406–416.

[102] N. Yamabe, T. Yokozawa, T. Oya, M. Kim, Therapeutic potential of (-)-epigallocatechin 3-O-gallate on renal damage in diabetic nephropathy model rats, J. Pharmacol. Exp. Ther. 319 (2006) 228–236.

[103] X.H. Yang, Y. Pan, X.L. Zhan, B.L. Zhang, L.L. Guo, H.M. Jin, Epigallocatechin-3-gallate Attenuates Renal Damage by Suppressing Oxidative Stress in Diabetic db/db Mice, Oxid. Med. Cell. Longev. 2016 (2016) 2968462.

[104] W. Sun, X. Liu, H. Zhang, Y. Song, T. Li, X. Liu, et al., Epigallocatechin gallate upregulates NRF2 to prevent diabetic nephropathy via disabling KEAP1, Free Radic. Biol. Med. 108 (2017) 840–857.

[105] Q.Q. Zhu, X.Y. Yang, X.J. Zhang, C.J. Yu, Q.Q. Pang, Y.W. Huang, et al., EGCG targeting Notch to attenuate renal fibrosis via inhibition of TGFβ/Smad3 signaling pathway activation in streptozotocin-induced diabetic mice, Food Funct. 11 (2020) 9686–9695.

[106] R. Yang, J. Chen, Q. Jia, X. Yang, S. Mehmood, Epigallocatechin-3-gallate ameliorates renal endoplasmic reticulum stress-mediated inflammation in type 2 diabetic rats, Exp. Biol. Med. (Maywood) 247 (2022) 1410–1419.

[107] J.G. Leu, C.Y. Lin, J.H. Jian, C.Y. Shih, Y.J. Liang, Epigallocatechin-3-gallate combined with alpha lipoic acid attenuates high glucose-induced receptor for advanced glycation end products (RAGE) expression in human embryonic kidney cells, An. Acad. Bras. Cienc. 85 (2013) 745–752.

[108] C. Xiang, X. Xiao, B. Jiang, M. Zhou, Y. Zhang, H. Li, et al., Epigallocatechin‑3‑gallate protects from high glucose induced podocyte apoptosis via suppressing endoplasmic reticulum stress, Mol. Med. Rep. 16 (2017) 6142–6147.

[109] S.P. Yoon, Y.H. Maeng, R. Hong, B.R. Lee, C.G. Kim, H.L. Kim, et al., Protective effects of epigallocatechin gallate (EGCG) on streptozotocin-induced diabetic nephropathy in mice, Acta Histochem. 116 (2014) 1210–1215.

[110] T. Mohan, K.K.S. Narasimhan, D.B. Ravi, P. Velusamy, N. Chandrasekar, L.N. Chakrapani, et al., Role of Nrf2 dysfunction in the pathogenesis of diabetic nephropathy: Therapeutic prospect of epigallocatechin-3-gallate, Free Radic. Biol. Med. 160 (2020) 227–238.

[111] T. Mohan, P. Velusamy, L.N. Chakrapani, A.K. Srinivasan, A. Singh, T. Johnson, et al., Impact of EGCG Supplementation on the Progression of Diabetic Nephropathy in Rats: An Insight into Fibrosis and Apoptosis, J. Agric. Food Chem. 65 (2017) 8028–8036.

[112] D. Álvarez-Cilleros, M.Á. Martín, L. Goya, S. Ramos, (-)-Epicatechin and the colonic metabolite 3,4-dihydroxyphenylacetic acid protect renal proximal tubular cell against high glucose-induced oxidative stress by modulating NOX-4/SIRT-1 signalling, J. Funct. Foods 46 (2018) 19–28.

[113] D. Álvarez Cilleros, M.E. López-Oliva, M. Martín, S. Ramos, (-)-Epicatechin and the colonic metabolite 2,3-dihydroxybenzoic acid protect against high glucose and lipopolysaccharide-induced inflammation in renal proximal tubular cells through NOX-4/p38 signalling, Food Funct. 11 (2020) 8811–8824.

[114] S. Malik, K. Suchal, S.I. Khan, J. Bhatia, K. Kishore, A.K. Dinda, et al., Apigenin ameliorates streptozotocin-induced diabetic nephropathy in rats via MAPK-NF-κB-TNF-α and TGF-β1-MAPK-fibronectin pathways, Am. J. Physiol. Renal Physiol. 313 (2017) F414–F422.

[115] J. Zhang, X. Zhao, H. Zhu, J. Wang, J. Ma, M. Gu, Apigenin Protects Against Renal Tubular Epithelial Cell Injury and Oxidative Stress by High Glucose via Regulation of NF-E2-Related Factor 2 (Nrf2) Pathway, Med. Sci. Monit. 25 (2019) 5280–5288.

[116] Y. Hou, Y. Zhang, S. Lin, Y. Yu, L. Yang, L. Li, et al., Protective mechanism of apigenin in diabetic nephropathy is related to its regulation of miR-423-5P-USF2 axis, Am. J. Transl. Res. 13 (2021) 2006–2020.

[117] T. Sapmaz Erçakalli, G. Coskun, D. Saker, L. Sencar, G. Gönlüşen, S. Polat, Effects of apigenin on mesangial GATA3 expression and PDGFR-β/NF-κB signaling pathway in experimental diabetic nephropathy model, Eur. J. Pharmacol. 1007 (2025) 178252.

[118] Y. Ogura, M. Kitada, J. Xu, I. Monno, D. Koya, CD38 inhibition by apigenin ameliorates mitochondrial oxidative stress through restoration of the intracellular NAD(+)/NADH ratio and Sirt3 activity in renal tubular cells in diabetic rats, Aging (Albany NY) 12 (2020) 11325–11336.

[119] M.-K. Kang, S.-H. Park, Y.-H. Kim, E.-J. Lee, L.D. Antika, D.Y. Kim, et al., Chrysin ameliorates podocyte injury and slit diaphragm protein loss via inhibition of the PERK-eIF2α-ATF-CHOP pathway in diabetic mice, Acta Pharmacol. Sin. 38 (2017) 1129–1140.

[120] A. Ahad, A.A. Ganai, M. Mujeeb, W.A. Siddiqui, Chrysin, an anti-inflammatory molecule, abrogates renal dysfunction in type 2 diabetic rats, Toxicol. Appl. Pharmacol. 279 (2014) 1–7.

[121] M.K. Kang, S.H. Park, Y.J. Choi, D. Shin, Y.H. Kang, Chrysin inhibits diabetic renal tubulointerstitial fibrosis through blocking epithelial to mesenchymal transition, J. Mol. Med. (Berl.) 93 (2015) 759–772.

[122] E.J. Lee, M.K. Kang, D.Y. Kim, Y.H. Kim, H. Oh, Y.H. Kang, Chrysin Inhibits Advanced Glycation End Products-Induced Kidney Fibrosis in Renal Mesangial Cells and Diabetic Kidneys, Nutrients 10 (7) (2018) 882.

[123] E.J. Lee, M.K. Kang, Y.H. Kim, D.Y. Kim, H. Oh, S.I. Kim, et al., Dietary Chrysin Suppresses Formation of Actin Cytoskeleton and Focal Adhesion in AGE-Exposed Mesangial Cells and Diabetic Kidney: Role of Autophagy, Nutrients 11 (1) (2019) 127.

[124] M.K. Kang, S.I. Kim, S.Y. Oh, W. Na, Y.H. Kang, Tangeretin Ameliorates Glucose-Induced Podocyte Injury through Blocking Epithelial to Mesenchymal Transition Caused by Oxidative Stress and Hypoxia, Int. J. Mol. Sci. 21 (22) (2020) 8577.

[125] F. Chen, Y. Ma, Z. Sun, X. Zhu, Tangeretin inhibits high glucose-induced extracellular matrix accumulation in human glomerular mesangial cells, Biomed. Pharmacother. 102 (2018) 1077–1083.

[126] Z. Liu, Y. Han, F. Zhao, Z. Zhao, J. Tian, K. Jia, Nobiletin suppresses high-glucose-induced inflammation and ECM accumulation in human mesangial cells through STAT3/NF-κB pathway, J. Cell. Biochem. 120 (2019) 3467–3473.

[127] P. Sun, R. Huang, Z. Qin, F. Liu, Influence of Tangeretin on the Exponential Regression of Inflammation and Oxidative Stress in Streptozotocin-Induced Diabetic Nephropathy, Appl. Biochem. Biotechnol. 194 (2022) 3914–3929.

[128] M. Xu, R. Wang, H. Fan, Z. Ni, Nobiletin ameliorates streptozotocin-cadmium-induced diabetic nephropathy via NF-κB signalling pathway in rats, Arch. Physiol. Biochem. 130 (2024) 29–37.

[129] Y. Liu, M. Zhang, L. Zeng, Y. Lai, S. Wu, X. Su, Wogonin upregulates SOCS3 to alleviate the injury in Diabetic Nephropathy by inhibiting TLR4-mediated JAK/STAT/AIM2 signaling pathway, Mol. Med. 30 (2024) 78.

[130] L. Lei, J. Zhao, X.Q. Liu, J. Chen, X.M. Qi, L.L. Xia, et al., Wogonin Alleviates Kidney Tubular Epithelial Injury in Diabetic Nephropathy by Inhibiting PI3K/Akt/NF-κB Signaling Pathways, Drug Des. Devel. Ther. 15 (2021) 3131–3150.

[131] Z.C. Zheng, W. Zhu, L. Lei, X.Q. Liu, Y.G. Wu, Wogonin Ameliorates Renal Inflammation and Fibrosis by Inhibiting NF-κB and TGF-β1/Smad3 Signaling Pathways in Diabetic Nephropathy, Drug Des. Devel. Ther. 14 (2020) 4135–4148.

[132] X.Q. Liu, L. Jiang, Y.Y. Li, Y.B. Huang, X.R. Hu, W. Zhu, et al., Wogonin protects glomerular podocytes by targeting Bcl-2-mediated autophagy and apoptosis in diabetic kidney disease, Acta Pharmacol. Sin. 43 (2022) 96–110.

[133] D. Li, H. Li, B. Shi, C. Li, Wogonin Alleviates Streptozotocin-stimulated Diabetic Nephropathy through its Antidiabetic, Antidyslipidemic, and Antioxidative Effects in Rats, Pharmacogn. Mag. 21 (2025) 218–228.

[134] A. Ahad, M. Mujeeb, H. Ahsan, W.A. Siddiqui, Prophylactic effect of baicalein against renal dysfunction in type 2 diabetic rats, Biochimie 106 (2014) 101–110.

[135] L. Chen, G. Tian, W. Tang, W. Luo, P. Liu, Z. Ma, Protective effect of luteolin on streptozotocin-induced diabetic renal damage in mice via the regulation of RIP140/NF-κB pathway and insulin signalling pathway, J. Funct. Foods 22 (2016) 93–100.

[136] G.G. Wang, X.H. Lu, W. Li, X. Zhao, C. Zhang, Protective Effects of Luteolin on Diabetic Nephropathy in STZ-Induced Diabetic Rats, Evid. Based Complement. Alternat. Med. 2011 (2011) 323171.

[137] M. Zhang, L. He, J. Liu, L. Zhou, Luteolin Attenuates Diabetic Nephropathy through Suppressing Inflammatory Response and Oxidative Stress by Inhibiting STAT3 Pathway, Exp. Clin. Endocrinol. Diabetes 129 (2021) 729–739.

[138] Q. Yu, M. Zhang, L. Qian, D. Wen, G. Wu, Luteolin attenuates high glucose-induced podocyte injury via suppressing NLRP3 inflammasome pathway, Life Sci. 225 (2019) 1–7.

[139] L.Y. Chen, H.L. Cheng, C.K. Liao, Y.H. Kuan, T.J. Liang, T.J. Tseng, et al., Luteolin improves nephropathy in hyperglycemic rats through anti-oxidant, anti-inflammatory, and anti-apoptotic mechanisms, J. Funct. Foods 102 (2023) 105461.

[140] Y. Yang, G. Chen, X. Cheng, Z. Teng, X. Cai, J. Yang, et al., Therapeutic potential of digitoflavone on diabetic nephropathy: nuclear factor erythroid 2-related factor 2-dependent anti-oxidant and anti-inflammatory effect, Sci. Rep. 5 (2015) 12377.

[141] Q. Yu, H. Mao, A. Vijayalakshmi, M. Zhou, Acacetin Prevents Renal Damage Induced by Streptozotocin via Altering the NF-κB/ASC/NLRP3 and AMPK/SIRT1 Pathways in Mice, Biotechnol. Appl. Biochem. 72 (2025) 1440–1449.

[142] H. Yu, M. Guo, Renal protection by acacetin in streptozotocin-induced diabetic nephropathy via TLR4/NF-κB pathway modulation in rats, Adv. Clin. Exp. Med. 34 (2025) 1353–1363.

[143] P. Kang, L. Xiao, Y. Liu, J. Yang, S. Li, L. Wang, Morusin ameliorates tubulointerstitial damage in diabetic mice through SIRT1/HIF-1α/IL-16 signaling pathway, Phytomedicine 142 (2025) 156781.

[144] F. Wu, S. Li, N. Zhang, W. Huang, X. Li, M. Wang, et al., Hispidulin alleviates high-glucose-induced podocyte injury by regulating protective autophagy, Biomed. Pharmacother. 104 (2018) 307–314.

[145] X.X. Xu, W. Zhang, P. Zhang, X.M. Qi, Y.G. Wu, J.J. Shen, Superior renoprotective effects of the combination of breviscapine with enalapril and its mechanism in diabetic rats, Phytomedicine 20 (2013) 820–827.

[146] J. Li, X. Huang, K. He, S. Wu, The kidney antifibrotic effects of 5,7,3',4',5'-pentamethoxyflavone from Bauhinia championii in streptozotocin-induced diabetic rats: in vivo and in vitro experiments, Pharm. Biol. 61 (2023) 938–948.

[147] M. Li, H.Y. Zhu, S.Y. Zhao, X.D. Li, S.M. Tong, J. Ma, et al., Baicalin alleviates lipid metabolism disorders in diabetic kidney disease via targeting FKBP51, Phytomedicine 139 (2025) 156473.

[148] L. Ma, F. Wu, Q. Shao, G. Chen, L. Xu, F. Lu, Baicalin Alleviates Oxidative Stress and Inflammation in Diabetic Nephropathy via Nrf2 and MAPK Signaling Pathway, Drug Des. Devel. Ther. 15 (2021) 3207–3221.

[149] J.E. Nam, S.Y. Jo, C.W. Ahn, Y.S. Kim, Baicalin attenuates fibrogenic process in human renal proximal tubular cells (HK-2) exposed to diabetic milieu, Life Sci. 254 (2020) 117742.

[150] B. Wang, N. Zhao, P. Ma, Q. Xu, Q. Chen, Baicalin Inhibits Kidney Inflammation in Mice with Diabetic Nephropathy by Modulating Toll-like Receptor 4/Nuclear Factor-Kappa B Signaling Pathway, Curr. Top. Nutraceutical Res. 19 (1) (2021) 115–119.

[151] S. Zhang, L. Xu, R. Liang, C. Yang, P. Wang, Baicalin suppresses renal fibrosis through microRNA-124/TLR4/NF-κB axis in streptozotocin-induced diabetic nephropathy mice and high glucose-treated human proximal tubule epithelial cells, J. Physiol. Biochem. 76 (2020) 407–416.

[152] H. Hu, W. Li, Y. Hao, Z. Peng, Z. Zou, W. Liang, Baicalin ameliorates renal fibrosis by upregulating CPT1α-mediated fatty acid oxidation in diabetic kidney disease, Phytomedicine 122 (2024) 155162.

[153] X.T. Zhang, G. Wang, L.F. Ye, Y. Pu, R.T. Li, J. Liang, et al., Baicalin reversal of DNA hypermethylation-associated Klotho suppression ameliorates renal injury in type 1 diabetic mouse model, Cell Cycle 19 (2020) 3329–3347.

[154] X.P. Zheng, Q. Nie, J. Feng, X.Y. Fan, Y.L. Jin, G. Chen, et al., Kidney-targeted baicalin-lysozyme conjugate ameliorates renal fibrosis in rats with diabetic nephropathy induced by streptozotocin, BMC Nephrol. 21 (2020) 174.

[155] J. Li, Y. Zhuang, G. Fan, S. Wang, E. Yan, J. Guo, et al., Impact of baicalin and components of Scutellaria baicalensis on renal fibrosis of diabetic kidney disease, Front. Pharmacol. 15 (2024) 1480626.

[156] J. Deng, C. Zheng, Z. Hua, H. Ci, G. Wang, L. Chen, Diosmin mitigates high glucose-induced endoplasmic reticulum stress through PI3K/AKT pathway in HK-2 cells, BMC Complement. Med. Ther. 22 (2022) 116.

[157] S. Ahmed, N. Mundhe, M. Borgohain, L. Chowdhury, M. Kwatra, N. Bolshette, et al., Diosmin Modulates the NF-kB Signal Transduction Pathways and Downregulation of Various Oxidative Stress Markers in Alloxan-Induced Diabetic Nephropathy, Inflammation 39 (2016) 1783–1797.

[158] Y. Jiang, J. Liu, Z. Zhou, K. Liu, C. Liu, Diosmetin Attenuates Akt Signaling Pathway by Modulating Nuclear Factor Kappa-Light-Chain-Enhancer of Activated B Cells (NF-κB)/Inducible Nitric Oxide Synthase (iNOS) in Streptozotocin (STZ)-Induced Diabetic Nephropathy Mice, Med. Sci. Monit. 24 (2018) 7007–7014.

[159] Z.L. Kong, K. Che, J.X. Hu, Y. Chen, Y.Y. Wang, X. Wang, et al., Orientin Protects Podocytes from High Glucose Induced Apoptosis through Mitophagy, Chem. Biodivers. 17 (2020) e1900647.

[160] X. Li, S. Zhao, M. Li, X. Xing, J. Xie, M. Wang, et al., Wogonoside ameliorates oxidative damage in tubular epithelial cells of diabetic nephropathy by modulating the HNF4A-NRF2 axis, Int. Immunopharmacol. 152 (2025) 114481.

[161] X. Li, S. Zhao, J. Xie, M. Li, S. Tong, J. Ma, et al., Targeting the NF-κB p65-MMP28 axis: Wogonoside as a novel therapeutic agent for attenuating podocyte injury in diabetic nephropathy, Phytomedicine 138 (2025) 156406.

[162] B. Huang, R. Han, H. Tan, W. Zhu, Y. Li, F. Jiang, et al., Scutellarin ameliorates diabetic nephropathy via TGF-β1 signaling pathway, Nat. Prod. Bioprospect. 14 (1) (2024) 25.

[163] S. Zhang, S. Zhang, H. Wang, Y. Chen, Vitexin ameliorated diabetic nephropathy via suppressing GPX4-mediated ferroptosis, Eur. J. Pharmacol. 951 (2023) 175787.

[164] G. Zhou, J. Cui, S. Xie, H. Wan, Y. Luo, G. Guo, Vitexin, a fenugreek glycoside, ameliorated obesity-induced diabetic nephropathy via modulation of NF-κB/IkBα and AMPK/ACC pathways in mice, Biosci. Biotechnol. Biochem. 85 (2021) 1183–1193.

[165] Y. Zhou, T. Hu, H. Zeng, L. Lin, H. Xie, R. Lin, et al., Naringenin Inhibits Ferroptosis in Renal Tubular Epithelial Cells of Diabetic Nephropathy Through SIRT1/FOXO3a Signaling Pathway, Drug Dev. Res. 86 (1) (2025) e70044.

[166] S.J. Tsai, C.S. Huang, M.C. Mong, W.Y. Kam, H.Y. Huang, M.C. Yin, Anti-inflammatory and antifibrotic effects of naringenin in diabetic mice, J. Agric. Food Chem. 60 (1) (2012) 514–521.

[167] Y. Ning, W. Li, P. Rui, L. Hongmei, L. Handeng, P. Huimin, et al., Naringenin Ameliorated Kidney Injury through Let-7a/TGFBR1 Signaling in Diabetic Nephropathy, J. Diabetes Res. 2016 (2016) 8738760.

[168] M.F. Khan, A. Mathur, V.K. Pandey, P. Kakkar, Naringenin alleviates hyperglycemia-induced renal toxicity by regulating activating transcription factor 4-C/EBP homologous protein mediated apoptosis, J. Cell Commun. Signal. 16 (2022) 271–291.

[169] S. Roy, F. Ahmed, S. Banerjee, U. Saha, Naringenin ameliorates streptozotocin-induced diabetic rat renal impairment by downregulation of TGF-β1 and IL-1 via modulation of oxidative stress correlates with decreased apoptotic events, Pharm. Biol. 54 (9) (2016) 1616–1627.

[170] A. Pérez, J. Mukdsi, L. Valdez, I. Rukavina-Mikusic, G. Díaz de Barboza, N. Tolosa de Talamoni, et al., Naringin prevents diabetic nephropathy in rats through blockage of oxidative stress and attenuation of the mitochondrial dysfunction, Can. J. Physiol. Pharmacol. 101 (2023) 349–360.

[171] S. Ding, H. Qiu, J. Huang, R. Chen, J. Zhang, B. Huang, et al., Activation of 20-HETE/PPARs involved in reno-therapeutic effect of naringenin on diabetic nephropathy, Chem. Biol. Interact. 307 (2019) 116–124.

[172] J. Zhang, S. Yang, H. Li, F. Chen, J. Shi, Naringin ameliorates diabetic nephropathy by inhibiting NADPH oxidase 4, Eur. J. Pharmacol. 804 (2017) 1–6.

[173] F. Chen, G. Wei, J. Xu, X. Ma, Q. Wang, Naringin ameliorates the high glucose-induced rat mesangial cell inflammatory reaction by modulating the NLRP3 Inflammasome, BMC Complement. Altern. Med. 18 (2018) 192.

[174] M.F. Khan, A. Mathur, V.K. Pandey, P. Kakkar, Naringenin alleviates hyperglycemia-induced renal toxicity by regulating activating transcription factor 4–C/EBP homologous protein mediated apoptosis, J. Cell Commun. Signal. 16 (2022) 271–291.

[175] Y.A. Kulkarni, S.V. Suryavanshi, Combination of Naringenin and Lisinopril Ameliorates Nephropathy in Type-1 Diabetic Rats, Endocr. Metab. Immune Disord. Drug Targets 21 (2021) 173–182.

[176] Y. Zhang, B. Wang, F. Guo, Z. Li, G. Qin, Involvement of the TGFβ1- ILK-Akt signaling pathway in the effects of hesperidin in type 2 diabetic nephropathy, Biomed. Pharmacother. 105 (2018) 766–772.

[177] Y.J. Chen, L. Kong, Z.Z. Tang, Y.M. Zhang, Y. Liu, T.Y. Wang, et al., Hesperetin ameliorates diabetic nephropathy in rats by activating Nrf2/ARE/glyoxalase 1 pathway, Biomed. Pharmacother. 111 (2019) 1166–1175.

[178] O.M. Ahmed, M.A. Hassan, A.S. Saleh, Combinatory effect of hesperetin and mesenchymal stem cells on the deteriorated lipid profile, heart and kidney functions and antioxidant activity in STZ-induced diabetic rats, BIOCELL 44 (1) (2020) 27–29.

[179] J.Z. AlTamimi, N.A. AlFaris, G.M. Alshammari, R.I. Alagal, D.H. Aljabryn, M. Abdo Yahya, Protective effect of eriodictyol against hyperglycemia-induced diabetic nephropathy in rats entails antioxidant and anti-inflammatory effects mediated by activating Nrf2, Saudi Pharm. J. 31 (2023) 101817.

[180] J. Bai, Y. Wang, X. Zhu, J. Shi, Eriodictyol inhibits high glucose-induced extracellular matrix accumulation, oxidative stress, and inflammation in human glomerular mesangial cells, Phytother. Res. 33 (2019) 2775–2782.

[181] O.S. Kim, Y.S. Kim, D.S. Jang, N.H. Yoo, J.S. Kim, Cytoprotection against hydrogen peroxide-induced cell death in cultured mouse mesangial cells by erigeroflavanone, a novel compound from the flowers of Erigeron annuus, Chem. Biol. Interact. 180 (2009) 414–420.

[182] J. Granados-Pineda, N. Uribe-Uribe, P. García-López, M.D.P. Ramos-Godinez, J.F. Rivero-Cruz, J.M. Pérez-Rojas, Effect of Pinocembrin Isolated from Mexican Brown Propolis on Diabetic Nephropathy, Molecules 23 (4) (2018) 852.

[183] X. Zhu, J. Shi, H. Li, Liquiritigenin attenuates high glucose-induced mesangial matrix accumulation, oxidative stress, and inflammation by suppression of the NF-κB and NLRP3 inflammasome pathways, Biomed. Pharmacother. 106 (2018) 976–982.

[184] N.N. Zhang, J.S. Kang, S.S. Liu, S.M. Gu, Z.P. Song, F.X. Li, et al., Flavanomarein inhibits high glucose-stimulated epithelial-mesenchymal transition in HK-2 cells via targeting spleen tyrosine kinase, Sci. Rep. 10 (2020) 439.

[185] D. Huang, P. Shen, C. Wang, J. Gao, C. Ye, F. Wu, Calycosin plays a protective role in diabetic kidney disease through the regulation of ferroptosis, Pharm. Biol. 60 (2022) 990–996.

[186] H. Yosri, D.H. El-Kashef, M. El-Sherbiny, E. Said, H.A. Salem, Calycosin modulates NLRP3 and TXNIP-mediated pyroptotic signaling and attenuates diabetic nephropathy progression in diabetic rats; An insight, Biomed. Pharmacother. 155 (2022) 113758.

[187] Y.Y. Zhang, R.Z. Tan, X.Q. Zhang, Y. Yu, C. Yu, Calycosin Ameliorates Diabetes-Induced Renal Inflammation via the NF-κB Pathway In Vitro and In Vivo, Med. Sci. Monit. 25 (2019) 1671–1678.

[188] N.M. Elsherbiny, E. Said, H. Atef, S.A. Zaitone, Renoprotective effect of calycosin in high fat diet-fed/STZ injected rats: Effect on IL-33/ST2 signaling, oxidative stress and fibrosis suppression, Chem. Biol. Interact. 315 (2020) 108897.

[189] C. Huang, L.F. Xue, B. Hu, H.H. Liu, S.B. Huang, S. Khan, et al., Calycosin-loaded nanoliposomes as potential nanoplatforms for treatment of diabetic nephropathy through regulation of mitochondrial respiratory function, J. Nanobiotechnol. 19 (1) (2021) 178.

[190] D. Tang, B. He, Z.G. Zheng, R.S. Wang, F. Gu, T.T. Duan, et al., Inhibitory effects of two major isoflavonoids in Radix Astragali on high glucose-induced mesangial cells proliferation and AGEs-induced endothelial cells apoptosis, Planta Med. 77 (2011) 729–732.

[191] K. Zhuang, X. Jiang, R. Liu, C. Ye, Y. Wang, Y. Wang, et al., Formononetin Activates the Nrf2/ARE Signaling Pathway Via Sirt1 to Improve Diabetic Renal Fibrosis, Front. Pharmacol. 11 (2020) 616378.

[192] M.J. Oza, Y.A. Kulkarni, Formononetin attenuates kidney damage in type 2 diabetic rats, Life Sci. 219 (2019) 109–121.

[193] S. Song, X. Zhou, L. Huang, J. Yu, Nephroprotective Effects of Formononetin in Diabetic Kidney Disease: Mechanistic Insights and Therapeutic Potential, Am. J. Chin. Med. 53 (2025) 2277–2305.

[194] Q. Huang, H. Chen, K. Yin, Y. Shen, K. Lin, X. Guo, et al., Formononetin Attenuates Renal Tubular Injury and Mitochondrial Damage in Diabetic Nephropathy Partly via Regulating Sirt1/PGC-1α Pathway, Front. Pharmacol. 13 (2022) 901234.

[195] M.J. Kim, Y. Lim, Protective effect of short-term genistein supplementation on the early stage in diabetes-induced renal damage, Mediators Inflamm. 2013 (2013) 510212.

[196] Y. Li, S. Ou, Q. Liu, L. Gan, L. Zhang, Y. Wang, et al., Genistein improves mitochondrial function and inflammatory in rats with diabetic nephropathy via inhibiting MAPK/NF-κB pathway, Acta Cir. Bras. 37 (2022) e370601.

[197] Y. Wang, Y. Li, T. Zhang, Y. Chi, M. Liu, Y. Liu, Genistein and Myd88 Activate Autophagy in High Glucose-Induced Renal Podocytes In Vitro, Med. Sci. Monit. 24 (2018) 4823–4831.

[198] W.J. Yuan, F.Y. Jia, J.Z. Meng, Effects of genistein on secretion of extracellular matrix components and transforming growth factor beta in high-glucose-cultured rat mesangial cells, J. Artif. Organs 12 (2009) 242–246.

[199] H.F. Jheng, K. Hayashi, Y. Matsumura, T. Kawada, S. Seno, H. Matsuda, et al., Anti-Inflammatory and Antioxidative Properties of Isoflavones Provide Renal Protective Effects Distinct from Those of Dietary Soy Proteins against Diabetic Nephropathy, Mol. Nutr. Food Res. 64 (2020) e2000015.

[200] S. Yang, C. Ma, H. Wu, H. Zhang, F. Yuan, G. Yang, et al., Tectorigenin attenuates diabetic nephropathy by improving vascular endothelium dysfunction through activating AdipoR1/2 pathway, Pharmacol. Res. 153 (2020) 104678.

[201] H. Tan, J. Chen, Y. Li, Y. Li, Y. Zhong, G. Li, et al., Glabridin, a bioactive component of licorice, ameliorates diabetic nephropathy by regulating ferroptosis and the VEGF/Akt/ERK pathways, Mol. Med. 28 (2022) 58.

[202] B. Hou, P. Ma, X. Yang, X. Zhao, L. Zhang, Y. Zhao, et al., In silico prediction and experimental validation to reveal the protective mechanism of Puerarin against excessive extracellular matrix accumulation through inhibiting ferroptosis in diabetic nephropathy, J. Ethnopharmacol. 319 (2024) 117281.

[203] C.P. Mao, Z.L. Gu, Puerarin reduces increased c-fos, c-jun, and type IV collagen expression caused by high glucose in glomerular mesangial cells, Acta Pharmacol. Sin. 26 (2005) 982–986.

[204] S. Xue, W. Fan, Q. Li, H. Huang, Y. Tang, M. Wu, Puerarin Attenuates Podocyte Damage in Mice With Diabetic Kidney Disease by Modulating the AMPK/Nrf2 Pathway, Int. J. Endocrinol. 2025 (2025) 4473803.

[205] Q. Chen, L. Wang, X. Wei, M. Chen, X. Zhang, R. Mo, et al., Puerarin alleviates diabetic nephropathy by inhibiting Caspase-1-mediated pyroptosis, J. Pharm. Pharmacol. 76 (2024) 213–223.

[206] X. Li, W. Cai, K. Lee, B. Liu, Y. Deng, Y. Chen, et al., Puerarin attenuates diabetic kidney injury through the suppression of NOX4 expression in podocytes, Sci. Rep. 7 (2017) 14603.

[207] Y. Zhang, H. Wang, L. Yu, J. Chen, The Puerarin improves renal function in STZ-induced diabetic rats by attenuating eNOS expression, Ren. Fail. 37 (2015) 699–703.

[208] Q. Zhu, S. Yang, C. Wei, G. Lu, K. Lee, J.C. He, et al., Puerarin attenuates diabetic kidney injury through interaction with Guanidine nucleotide-binding protein Gi subunit alpha-1 (Gnai1) subunit, J. Cell. Mol. Med. 26 (2022) 3816–3827.

[209] X. Pan, J. Wang, Y. Pu, J. Yao, H. Wang, Effect of Puerarin on Expression of ICAM-1 and TNF-α in Kidneys of Diabetic Rats, Med. Sci. Monit. 21 (2015) 2134–2140.

[210] H. Wang, W. Li, Puerarin alleviates the high glucose-induced oxidative stress via the RAGE/PKC/NOX4 axis in renal mesangial cells, J. Toxicol. Sci. 49 (2024) 497–507.

[211] X. Xu, N. Zheng, Z. Chen, W. Huang, T. Liang, H. Kuang, Puerarin, isolated from Pueraria lobata (Willd.), protects against diabetic nephropathy by attenuating oxidative stress, Gene 591 (2016) 411–416.

[212] X. Li, J. Wang, J. Yan, J.C. He, Y. Li, Y. Zhong, Additive renal protective effects between arctigenin and puerarin in diabetic kidney disease, Biomed. Pharmacother. 171 (2024) 116107.

[213] X. Li, Q. Zhu, R. Zheng, J. Yan, M. Wei, Y. Fan, et al., Puerarin Attenuates Diabetic Nephropathy by Promoting Autophagy in Podocytes, Front. Physiol. 11 (2020) 73.

[214] Q. Li, Y. Xiao, H. Gong, D. Shen, F. Zhu, Q. Wu, et al., Effect of puerarin on the expression of extracellular matrix in rats with streptozotocin-induced diabetic nephropathy, Natl. Med. J. India 22 (2009) 9–12.

[215] Y. Zhong, X. Zhang, X. Cai, K. Wang, Y. Chen, Y. Deng, Puerarin attenuated early diabetic kidney injury through down-regulation of matrix metalloproteinase 9 in streptozotocin-induced diabetic rats, PLoS One 9 (2014) e85690.

[216] L. Wang, X. Xie, Q. Chen, Y. Chen, X. Xu, T. Liang, Puerarin reduces diabetic nephropathy-induced podocyte pyroptosis by modulating the SIRT1/NLRP3/caspase-1 pathway, Mol. Cell. Endocrinol. 595 (2025) 112409.

[217] Y. Wang, J. Yang, X. Chang, Y. Xue, G. Liu, T. Zhang, et al., Isoliquiritigenin Alleviates Diabetic Kidney Disease via Oxidative Stress and the TLR4/NF-κB/NLRP3 Inflammasome Pathway, Mol. Nutr. Food Res. 68 (16) (2024) e2400215.

[218] Q. Shi, T. Zhou, W. Hou, Y. Zhou, S. Deng, Y. Song, Isoliquiritigenin Protects Against Diabetic Nephropathy in db/db Mice by Inhibiting Advanced Glycation End Product-Receptor for Advanced Glycation End Product Axis, Drug Dev. Res. 86 (2025) e70051.

[219] J. Li, S.W. Kang, J.L. Kim, H.Y. Sung, I.S. Kwun, Y.H. Kang, Isoliquiritigenin entails blockade of TGF-beta1-SMAD signaling for retarding high glucose-induced mesangial matrix accumulation, J. Agric. Food Chem. 58 (2010) 3205–3212.

[220] L. Sun, Z. Yang, J. Zhang, J. Wang, Isoliquiritigenin attenuates acute renal injury through suppressing oxidative stress, fibrosis and JAK2/STAT3 pathway in streptozotocin-induced diabetic rats, Bioengineered 12 (2021) 11188–11200.

[221] C.Y. Lin, Y.C. Lin, C.R. Paul, D.J. Hsieh, C.H. Day, R.J. Chen, et al., Isoliquiritigenin ameliorates advanced glycation end-products toxicity on renal proximal tubular epithelial cells, Environ. Toxicol. 37 (2022) 2096–2102.

[222] X. Huang, Y. Shi, H. Chen, R. Le, X. Gong, K. Xu, et al., Isoliquiritigenin prevents hyperglycemia-induced renal injuries by inhibiting inflammation and oxidative stress via SIRT1-dependent mechanism, Cell Death Dis. 11 (12) (2020) 1040.

[223] W.H. Dong, Q.Q. Chu, S.Q. Liu, D.T. Deng, Q. Xu, Isobavachalcone ameliorates diabetic nephropathy in rats by inhibiting the NF-κB pathway, J. Food Biochem. 44 (2020) e13405.

[224] E.S. Lee, M.H. Kwon, H.M. Kim, N. Kim, Y.M. Kim, H.S. Kim, et al., Dibenzoylmethane ameliorates lipid-induced inflammation and oxidative injury in diabetic nephropathy, J. Endocrinol. 240 (2019) 169–179.

[225] Y. Guo, Z. Ran, Y. Zhang, Z. Song, L. Wang, L. Yao, et al., Marein ameliorates diabetic nephropathy by inhibiting renal sodium glucose transporter 2 and activating the AMPK signaling pathway in db/db mice and high glucose-treated HK-2 cells, Biomed. Pharmacother. 131 (2020) 110684.

[226] F. Chen, X. Zhu, Z. Sun, Y. Ma, Astilbin Inhibits High Glucose-Induced Inflammation and Extracellular Matrix Accumulation by Suppressing the TLR4/MyD88/NF-κB Pathway in Rat Glomerular Mesangial Cells, Front. Pharmacol. 9 (2018) 1187.

[227] G.S. Li, W.L. Jiang, X.D. Yue, G.W. Qu, J.W. Tian, J. Wu, et al., Effect of astilbin on experimental diabetic nephropathy in vivo and in vitro, Planta Med. 75 (2009) 1470–1475.

[228] F. Chen, Z. Sun, X. Zhu, Y. Ma, Astilbin inhibits high glucose-induced autophagy and apoptosis through the PI3K/Akt pathway in human proximal tubular epithelial cells, Biomed. Pharmacother. 106 (2018) 1175–1181.

[229] C. Dong, G. Wu, H. Li, Y. Qiao, S. Gao, Ampelopsin inhibits high glucose-induced extracellular matrix accumulation and oxidative stress in mesangial cells through activating the Nrf2/HO-1 pathway, Phytother. Res. 34 (2020) 2044–2052.

[230] L. Guo, K. Tan, Q. Luo, X. Bai, Dihydromyricetin promotes autophagy and attenuates renal interstitial fibrosis by regulating miR-155-5p/PTEN signaling in diabetic nephropathy, Bosn. J. Basic Med. Sci. 20 (2020) 372–380.

[231] M. Wen, X. Sun, L. Pan, S. Jing, X. Zhang, L. Liang, et al., Dihydromyricetin ameliorates diabetic renal fibrosis via regulating SphK1 to suppress the activation of NF-κB pathway, Eur. J. Pharmacol. 978 (2024) 176799.

[232] T. Ding, S. Wang, X. Zhang, W. Zai, J. Fan, W. Chen, et al., Kidney protection effects of dihydroquercetin on diabetic nephropathy through suppressing ROS and NLRP3 inflammasome, Phytomedicine 41 (2018) 45–53.

[233] Y. Qin, Q. Zhai, Y. Li, M. Cao, Y. Xu, K. Zhao, et al., Cyanidin-3-O-glucoside ameliorates diabetic nephropathy through regulation of glutathione pool, Biomed. Pharmacother. 103 (2018) 1223–1230.

[234] H.X. Zheng, S.S. Qi, J. He, C.Y. Hu, H. Han, H. Jiang, et al., Cyanidin-3-glucoside from Black Rice Ameliorates Diabetic Nephropathy via Reducing Blood Glucose, Suppressing Oxidative Stress and Inflammation, and Regulating Transforming Growth Factor β1/Smad Expression, J. Agric. Food Chem. 68 (2020) 4399–4410.

[235] C. Du, Y. Shi, Y. Ren, H. Wu, F. Yao, J. Wei, et al., Anthocyanins inhibit high-glucose-induced cholesterol accumulation and inflammation by activating LXRα pathway in HK-2 cells, Drug Des. Devel. Ther. 9 (2015) 5099–5113.

[236] S. Wang, Y. Huang, G. Luo, X. Yang, W. Huang, Cyanidin-3-O-glucoside attenuates high glucose-induced podocyte dysfunction by inhibiting apoptosis and promoting autophagy via activation of SIRT1/AMPK pathway, Can. J. Physiol. Pharmacol. 99 (2021) 589–598.

[237] Y.X. Li, Y.P. Lu, D. Tang, B. Hu, Z.Y. Zhang, H.W. Wu, et al., Anthocyanin improves kidney function in diabetic kidney disease by regulating amino acid metabolism, J. Transl. Med. 20 (2022) 510.

[238] J. Li, S.S. Lim, J.Y. Lee, J.K. Kim, S.W. Kang, J.L. Kim, et al., Purple corn anthocyanins dampened high-glucose-induced mesangial fibrosis and inflammation: possible renoprotective role in diabetic nephropathy, J. Nutr. Biochem. 23 (2012) 320–331.

[239] W. Han, Z. Deng, J. Li, B. Ma, Z. Lu, Y. Zhang, et al., Mechanisms of Complanatoside A against extracellular matrix accumulation, inflammation and proliferation of mesangial cells in diabetic nephropathy through network pharmacology and experimental validation, Tissue Cell 96 (2025) 103031.

[240] J. Li, S.S. Lim, E.S. Lee, J.H. Gong, D. Shin, I.J. Kang, et al., Isoangustone A suppresses mesangial fibrosis and inflammation in human renal mesangial cells, Exp. Biol. Med. (Maywood) 236 (2011) 435–444.

[241] L. Wei, P. Jian, H. Erjiong, Z. Qihan, Ginkgetin alleviates high glucose-evoked mesangial cell oxidative stress injury, inflammation, and extracellular matrix (ECM) deposition in an AMPK/mTOR-mediated autophagy axis, Chem. Biol. Drug Des. 98 (2021) 620–630.

[242] K. Singh, K.S. Yadav, A. Shukla, V.K. Singh, N.P. Yadav, M.N. Mugale, et al., Cupressuflavone Isolated from Cupressus torulosa Ameliorates Diabetic Nephropathy by Inhibiting Oxidative Stress and Inflammation Through Nrf-2/NF-κB Signalling Axis, Planta Med. 91 (2025) 913–922.

[243] R. Liu, Q. Wang, Z. Ding, X. Zhang, Y. Li, Y. Zang, et al., Silibinin Augments the Antifibrotic Effect of Valsartan Through Inactivation of TGF-β1 Signaling in Kidney, Drug Des. Devel. Ther. 14 (2020) 603–611.

[244] K. Khazim, Y. Gorin, R.C. Cavaglieri, H.E. Abboud, P. Fanti, The antioxidant silybin prevents high glucose-induced oxidative stress and podocyte injury in vitro and in vivo, Am. J. Physiol. Renal Physiol. 305 (2013) F691–F700.

[245] K. Liu, S. Zhou, J. Liu, Y. Wang, F. Zhu, M. Liu, Silibinin attenuates high-fat diet-induced renal fibrosis of diabetic nephropathy, Drug Des. Devel. Ther. 13 (2019) 3117–3126.

[246] Y. Chen, L. Chen, T. Yang, Silymarin nanoliposomes attenuate renal injury on diabetic nephropathy rats via co-suppressing TGF-β/Smad and JAK2/STAT3/SOCS1 pathway, Life Sci. 271 (2021) 119197.

[247] C. Soto, J. Pérez, V. García, E. Uría, M. Vadillo, L. Raya, Effect of silymarin on kidneys of rats suffering from alloxan-induced diabetes mellitus, Phytomedicine 17 (2010) 1090–1094.

[248] F. Bin, R. Meng, H. Bin, Y. Bi, S. Shen, D. Zhu, Silymarin protects against renal injury through normalization of lipid metabolism and mitochondrial biogenesis in high fat-fed mice, Free Radic. Biol. Med. 110 (2017) 240–249.

[249] F. Li, J. Zhang, L. Luo, J. Hu, Protective Effects of Xanthohumol against Diabetic Nephropathy in a Mouse Model, Kidney Blood Press. Res. 48 (2023) 92–101.

[250] J. Singh, B.P. Chaudhari, P. Kakkar, Baicalin and chrysin mixture imparts cyto-protection against methylglyoxal induced cytotoxicity and diabetic tubular injury by modulating RAGE, oxidative stress and inflammation, Environ. Toxicol. Pharmacol. 50 (2017) 67–75.

[251] J. Wei, H. Wu, H. Zhang, F. Li, S. Chen, B. Hou, et al., Anthocyanins inhibit high glucose-induced renal tubular cell apoptosis caused by oxidative stress in db/db mice, Int. J. Mol. Med. 41 (2018) 1608–1618.rm Biol. 2022;60:990-6.

[186] Yosri H, El-Kashef DH, El-Sherbiny M, Said E, Salem HA. Calycosin modulates NLRP3 and TXNIP-mediated pyroptotic signaling and attenuates diabetic nephropathy progression in diabetic rats; An insight. Biomed Pharmacother. 2022;155:113758.

[187] Zhang YY, Tan RZ, Zhang XQ, Yu Y, Yu C. Calycosin Ameliorates Diabetes-Induced Renal Inflammation via the NF-κB Pathway In Vitro and In Vivo. Med Sci Monit. 2019;25:1671-8.

[188] Elsherbiny NM, Said E, Atef H, Zaitone SA. Renoprotective effect of calycosin in high fat diet-fed/STZ injected rats: Effect on IL-33/ST2 signaling, oxidative stress and fibrosis suppression. Chem Biol Interact. 2020;315:108897.

[189] Huang C, Xue LF, Hu B, Liu HH, Meng Y. Calycosin-loaded nanoliposomes as potential nanoplatforms for treatment of diabetic nephropathy through regulation of mitochondrial respiratory function. Journal of nanobiotechnology.19:178.

[190] Tang D, He B, Zheng ZG, Wang RS, Gu F, Duan TT, et al. Inhibitory effects of two major isoflavonoids in Radix Astragali on high glucose-induced mesangial cells proliferation and AGEs-induced endothelial cells apoptosis. Planta Med. 2011;77:729-32.

[191] Zhuang K, Jiang X, Liu R, Ye C, Wang Y, Wang Y, et al. Formononetin Activates the Nrf2/ARE Signaling Pathway Via Sirt1 to Improve Diabetic Renal Fibrosis. Front Pharmacol. 2020;11:616378.

[192] Oza MJ, Kulkarni YA. Formononetin attenuates kidney damage in type 2 diabetic rats. Life Sci. 2019;219:109-21.

[193] Song S, Zhou X, Huang L, Yu J. Nephroprotective Effects of Formononetin in Diabetic Kidney Disease: Mechanistic Insights and Therapeutic Potential. Am J Chin Med. 2025;53:2277-305.

[194] Huang Q, Chen H, Yin K, Shen Y, Lin K, Guo X, et al. Formononetin Attenuates Renal Tubular Injury and Mitochondrial Damage in Diabetic Nephropathy Partly via Regulating Sirt1/PGC-1α Pathway. Front Pharmacol. 2022;13:901234.

[195] Kim MJ, Lim Y. Protective effect of short-term genistein supplementation on the early stage in diabetes-induced renal damage. Mediators Inflamm. 2013;2013:510212.

[196] Li Y, Ou S, Liu Q, Gan L, Zhang L, Wang Y, et al. Genistein improves mitochondrial function and inflammatory in rats with diabetic nephropathy via inhibiting MAPK/NF-κB pathway. Acta Cir Bras. 2022;37:e370601.

[197] Wang Y, Li Y, Zhang T, Chi Y, Liu M, Liu Y. Genistein and Myd88 Activate Autophagy in High Glucose-Induced Renal Podocytes In Vitro. Med Sci Monit. 2018;24:4823-31.

[198] Yuan WJ, Jia FY, Meng JZ. Effects of genistein on secretion of extracellular matrix components and transforming growth factor beta in high-glucose-cultured rat mesangial cells. J Artif Organs. 2009;12:242-6.

[199] Jheng HF, Hayashi K, Matsumura Y, Kawada T, Seno S, Matsuda H, et al. Anti-Inflammatory and Antioxidative Properties of Isoflavones Provide Renal Protective Effects Distinct from Those of Dietary Soy Proteins against Diabetic Nephropathy. Mol Nutr Food Res. 2020;64:e2000015.

[200] Yang S, Ma C, Wu H, Zhang H, Yuan F, Yang G, et al. Tectorigenin attenuates diabetic nephropathy by improving vascular endothelium dysfunction through activating AdipoR1/2 pathway. Pharmacol Res. 2020;153:104678.

[201] Tan H, Chen J, Li Y, Li Y, Zhong Y, Li G, et al. Glabridin, a bioactive component of licorice, ameliorates diabetic nephropathy by regulating ferroptosis and the VEGF/Akt/ERK pathways. Mol Med. 2022;28:58.

[202] Hou B, Ma P, Yang X, Zhao X, Zhang L, Zhao Y, et al. In silico prediction and experimental validation to reveal the protective mechanism of Puerarin against excessive extracellular matrix accumulation through inhibiting ferroptosis in diabetic nephropathy. J Ethnopharmacol. 2024;319:117281.

[203] Mao CP, Gu ZL. Puerarin reduces increased c-fos, c-jun, and type IV collagen expression caused by high glucose in glomerular mesangial cells. Acta Pharmacol Sin. 2005;26:982-6.

[204] Xue S, Fan W, Li Q, Huang H, Tang Y, Wu M. Puerarin Attenuates Podocyte Damage in Mice With Diabetic Kidney Disease by Modulating the AMPK/Nrf2 Pathway. Int J Endocrinol. 2025;2025:4473803.

[205] Chen Q, Wang L, Wei X, Chen M, Zhang X, Mo R, et al. Puerarin alleviates diabetic nephropathy by inhibiting Caspase-1-mediated pyroptosis. J Pharm Pharmacol. 2024;76:213-23.

[206] Li X, Cai W, Lee K, Liu B, Deng Y, Chen Y, et al. Puerarin attenuates diabetic kidney injury through the suppression of NOX4 expression in podocytes. Sci Rep. 2017;7:14603.

[207] Zhang Y, Wang H, Yu L, Chen J. The Puerarin improves renal function in STZ-induced diabetic rats by attenuating eNOS expression. Ren Fail. 2015;37:699-703.

[208] Zhu Q, Yang S, Wei C, Lu G, Lee K, He JC, et al. Puerarin attenuates diabetic kidney injury through interaction with Guanidine nucleotide-binding protein Gi subunit alpha-1 (Gnai1) subunit. J Cell Mol Med. 2022;26:3816-27.

[209] Pan X, Wang J, Pu Y, Yao J, Wang H. Effect of Puerarin on Expression of ICAM-1 and TNF-α in Kidneys of Diabetic Rats. Med Sci Monit. 2015;21:2134-40.

[210] Wang H, Li W. Puerarin alleviates the high glucose-induced oxidative stress via the RAGE/PKC/NOX4 axis in renal mesangial cells. J Toxicol Sci. 2024;49:497-507.

[211] Xu X, Zheng N, Chen Z, Huang W, Liang T, Kuang H. Puerarin, isolated from Pueraria lobata (Willd.), protects against diabetic nephropathy by attenuating oxidative stress. Gene. 2016;591:411-6.

[212] Li X, Wang J, Yan J, He JC, Li Y, Zhong Y. Additive renal protective effects between arctigenin and puerarin in diabetic kidney disease. Biomed Pharmacother. 2024;171:116107.

[213] Li X, Zhu Q, Zheng R, Yan J, Wei M, Fan Y, et al. Puerarin Attenuates Diabetic Nephropathy by Promoting Autophagy in Podocytes. Front Physiol. 2020;11:73.

[214] Li Q, Xiao Y, Gong H, Shen D, Zhu F, Wu Q, et al. Effect of puerarin on the expression of extracellular matrix in rats with streptozotocin-induced diabetic nephropathy. Natl Med J India. 2009;22:9-12.

[215] Zhong Y, Zhang X, Cai X, Wang K, Chen Y, Deng Y. Puerarin attenuated early diabetic kidney injury through down-regulation of matrix metalloproteinase 9 in streptozotocin-induced diabetic rats. PLoS One. 2014;9:e85690.

[216] Wang L, Xie X, Chen Q, Chen Y, Xu X, Liang T. Puerarin reduces diabetic nephropathy-induced podocyte pyroptosis by modulating the SIRT1/NLRP3/caspase-1 pathway. Mol Cell Endocrinol. 2025;595:112409.

[217] Wang Y, Yang J, Chang X, Xue Y, Liu G, Zhang T, et al. Isoliquiritigenin Alleviates Diabetic Kidney Disease via Oxidative Stress and the TLR4/NF-κB/NLRP3 Inflammasome Pathway. Molecular Nutrition & Food Research. 2024;68.

[218] Shi Q, Zhou T, Hou W, Zhou Y, Deng S, Song Y. Isoliquiritigenin Protects Against Diabetic Nephropathy in db/db Mice by Inhibiting Advanced Glycation End Product-Receptor for Advanced Glycation End Product Axis. Drug Dev Res. 2025;86:e70051.

[219] Li J, Kang SW, Kim JL, Sung HY, Kwun IS, Kang YH. Isoliquiritigenin entails blockade of TGF-beta1-SMAD signaling for retarding high glucose-induced mesangial matrix accumulation. J Agric Food Chem. 2010;58:3205-12.

[220] Sun L, Yang Z, Zhang J, Wang J. Isoliquiritigenin attenuates acute renal injury through suppressing oxidative stress, fibrosis and JAK2/STAT3 pathway in streptozotocin-induced diabetic rats. Bioengineered. 2021;12:11188-200.

[221] Lin CY, Lin YC, Paul CR, Hsieh DJ, Day CH, Chen RJ, et al. Isoliquiritigenin ameliorates advanced glycation end-products toxicity on renal proximal tubular epithelial cells. Environ Toxicol. 2022;37:2096-102.

[222] Huang X, Shi Y, Chen H, Le R, Gong X, Xu K, et al. Isoliquiritigenin prevents hyperglycemia-induced renal injuries by inhibiting inflammation and oxidative stress via SIRT1-dependent mechanism. Cell Death & Disease. 2020;11.

[223] Dong WH, Chu QQ, Liu SQ, Deng DT, Xu Q. Isobavachalcone ameliorates diabetic nephropathy in rats by inhibiting the NF-κB pathway. J Food Biochem. 2020;44:e13405.

[224] Lee ES, Kwon MH, Kim HM, Kim N, Kim YM, Kim HS, et al. Dibenzoylmethane ameliorates lipid-induced inflammation and oxidative injury in diabetic nephropathy. J Endocrinol. 2019;240:169-79.

[225] Guo Y, Ran Z, Zhang Y, Song Z, Wang L, Yao L, et al. Marein ameliorates diabetic nephropathy by inhibiting renal sodium glucose transporter 2 and activating the AMPK signaling pathway in db/db mice and high glucose-treated HK-2 cells. Biomed Pharmacother. 2020;131:110684.

[226] Chen F, Zhu X, Sun Z, Ma Y. Astilbin Inhibits High Glucose-Induced Inflammation and Extracellular Matrix Accumulation by Suppressing the TLR4/MyD88/NF-κB Pathway in Rat Glomerular Mesangial Cells. Frontiers in Pharmacology. 2018;9.

[227] Li GS, Jiang WL, Yue XD, Qu GW, Tian JW, Wu J, et al. Effect of astilbin on experimental diabetic nephropathy in vivo and in vitro. Planta Med. 2009;75:1470-5.

[228] Chen F, Sun Z, Zhu X, Ma Y. Astilbin inhibits high glucose-induced autophagy and apoptosis through the PI3K/Akt pathway in human proximal tubular epithelial cells. Biomed Pharmacother. 2018;106:1175-81.

[229] Dong C, Wu G, Li H, Qiao Y, Gao S. Ampelopsin inhibits high glucose-induced extracellular matrix accumulation and oxidative stress in mesangial cells through activating the Nrf2/HO-1 pathway. Phytotherapy Research. 2020;34:2044-52.

[230] Guo L, Tan K, Luo Q, Bai X. Dihydromyricetin promotes autophagy and attenuates renal interstitial fibrosis by regulating miR-155-5p/PTEN signaling in diabetic nephropathy. Bosn J Basic Med Sci. 2020;20:372-80.

[231] Wen M, Sun X, Pan L, Jing S, Zhang X, Liang L, et al. Dihydromyricetin ameliorates diabetic renal fibrosis via regulating SphK1 to suppress the activation of NF-κB pathway. Eur J Pharmacol. 2024;978:176799.

[232] Ding T, Wang S, Zhang X, Zai W, Fan J, Chen W, et al. Kidney protection effects of dihydroquercetin on diabetic nephropathy through suppressing ROS and NLRP3 inflammasome. Phytomedicine. 2018;41:45-53.

[233] Qin Y, Zhai Q, Li Y, Cao M, Xu Y, Zhao K, et al. Cyanidin-3-O-glucoside ameliorates diabetic nephropathy through regulation of glutathione pool. Biomed Pharmacother. 2018;103:1223-30.

[234] Zheng HX, Qi SS, He J, Hu CY, Han H, Jiang H, et al. Cyanidin-3-glucoside from Black Rice Ameliorates Diabetic Nephropathy via Reducing Blood Glucose, Suppressing Oxidative Stress and Inflammation, and Regulating Transforming Growth Factor β1/Smad Expression. J Agric Food Chem. 2020;68:4399-410.

[235] Du C, Shi Y, Ren Y, Wu H, Yao F, Wei J, et al. Anthocyanins inhibit high-glucose-induced cholesterol accumulation and inflammation by activating LXRα pathway in HK-2 cells. Drug Des Devel Ther. 2015;9:5099-113.

[236] Wang S, Huang Y, Luo G, Yang X, Huang W. Cyanidin-3-O-glucoside attenuates high glucose-induced podocyte dysfunction by inhibiting apoptosis and promoting autophagy via activation of SIRT1/AMPK pathway. Can J Physiol Pharmacol. 2021;99:589-98.

[237] Li YX, Lu YP, Tang D, Hu B, Zhang ZY, Wu HW, et al. Anthocyanin improves kidney function in diabetic kidney disease by regulating amino acid metabolism. J Transl Med. 2022;20:510.

[238] Li J, Lim SS, Lee JY, Kim JK, Kang SW, Kim JL, et al. Purple corn anthocyanins dampened high-glucose-induced mesangial fibrosis and inflammation: possible renoprotective role in diabetic nephropathy. J Nutr Biochem. 2012;23:320-31.

[239] Han W, Deng Z, Li J, Ma B, Lu Z, Zhang Y, et al. Mechanisms of Complanatoside A against extracellular matrix accumulation, inflammation and proliferation of mesangial cells in diabetic nephropathy through network pharmacology and experimental validation. Tissue Cell. 2025;96:103031.

[240] Li J, Lim SS, Lee ES, Gong JH, Shin D, Kang IJ, et al. Isoangustone A suppresses mesangial fibrosis and inflammation in human renal mesangial cells. Exp Biol Med (Maywood). 2011;236:435-44.

[241] Wei L, Jian P, Erjiong H, Qihan Z. Ginkgetin alleviates high glucose-evoked mesangial cell oxidative stress injury, inflammation, and extracellular matrix (ECM) deposition in an AMPK/mTOR-mediated autophagy axis. Chem Biol Drug Des. 2021;98:620-30.

[242] Singh K, Yadav KS, Shukla A, Singh VK, Yadav NP, Mugale MN, et al. Cupressuflavone Isolated from Cupressus torulosa Ameliorates Diabetic Nephropathy by Inhibiting Oxidative Stress and Inflammation Through Nrf-2/NF-κB Signalling Axis. Planta Med. 2025;91:913-22.

[243] Liu R, Wang Q, Ding Z, Zhang X, Li Y, Zang Y, et al. Silibinin Augments the Antifibrotic Effect of Valsartan Through Inactivation of TGF-β1 Signaling in Kidney. Drug Des Devel Ther. 2020;14:603-11.

[244] Khazim K, Gorin Y, Cavaglieri RC, Abboud HE, Fanti P. The antioxidant silybin prevents high glucose-induced oxidative stress and podocyte injury in vitro and in vivo. Am J Physiol Renal Physiol. 2013;305:F691-700.

[245] Liu K, Zhou S, Liu J, Wang Y, Zhu F, Liu M. Silibinin attenuates high-fat diet-induced renal fibrosis of diabetic nephropathy. Drug Des Devel Ther. 2019;13:3117-26.

[246] Chen Y, Chen L, Yang T. Silymarin nanoliposomes attenuate renal injury on diabetic nephropathy rats via co-suppressing TGF-β/Smad and JAK2/STAT3/SOCS1 pathway. Life Sci. 2021;271:119197.

[247] Soto C, Pérez J, García V, Uría E, Vadillo M, Raya L. Effect of silymarin on kidneys of rats suffering from alloxan-induced diabetes mellitus. Phytomedicine. 2010;17:1090-4.

[248] Bin F, Meng R, Bin H, Bi Y, Shen S, Zhu D. Silymarin protects against renal injury through normalization of lipid metabolism and mitochondrial biogenesis in high fat-fed mice. Free Radic Biol Med. 2017;110:240-9.

[249] Li F, Zhang J, Luo L, Hu J. Protective Effects of Xanthohumol against Diabetic Nephropathy in a Mouse Model. Kidney Blood Press Res. 2023;48:92-101.

[250] Singh J, Chaudhari BP, Kakkar P. Baicalin and chrysin mixture imparts cyto-protection against methylglyoxal induced cytotoxicity and diabetic tubular injury by modulating RAGE, oxidative stress and inflammation. Environ Toxicol Pharmacol. 2017;50:67-75.

[251] Wei J, Wu H, Zhang H, Li F, Chen S, Hou B, et al. Anthocyanins inhibit high glucose-induced renal tubular cell apoptosis caused by oxidative stress in db/db mice. Int J Mol Med. 2018;41:1608-18.
